# Pyoderma gangrenosum caused by the molecular uncoupling of OTULIN catalytic activity and LUBAC binding

**DOI:** 10.1038/s41590-026-02568-6

**Published:** 2026-06-15

**Authors:** Barathram Swaminathan, Hwi M. Gil, Sagar Bhattad, Jyothi Janardhanan, Gerardo Mejía Baltodano, Christine Mariskanish, Shamel Basaria, Joseph M. Choi, Qi Liu, Lisette M. Scheepmaker, Mohamud Mohamed, Bertrand Boisson, Jean-Laurent Casanova, Ivona Aksentijevich, András N. Spaan, Janet G. Markle

**Affiliations:** 1 Department of Medical Microbiology, University Medical Center Utrecht, Utrecht University, Utrecht, The Netherlands; 2 Division of Molecular Pathogenesis, Department of Pathology, Microbiology & Immunology, Vanderbilt University Medical Center, Nashville, TN USA; 3 Division of Pediatric Immunology and Rheumatology, Department of Pediatrics, Aster CMI Hospital, Bengaluru, India; 4 National Head of Genetics Specialty, Ministerio de Salud, Managua, Nicaragua; 5 Division of Genetic Medicine, Department of Medicine, Vanderbilt University Medical Center, Nashville, TN USA; 6 St. Giles Laboratory of Human Genetics of Infectious Diseases, The Rockefeller University, New York, NY USA; 7 Laboratory of Human Genetics of Infectious Diseases, INSERM UMR1163, Paris, France; 8 Imagine Institute, University of Paris Cité, Paris, France; 9 Department of Pediatrics, Necker Hospital for Sick Children, Paris, France; 10 Howard Hughes Medical Institute, New York, NY USA; 11 Inflammatory Disease Section, National Human Genome Research Institute, National Institutes of Health, Bethesda, MD USA; 12 Present Address: Pediatric Immunology, Rheumatology and BMT, Manipal Hospital – Yelahanka, Bengaluru, India

**Keywords:** Immunogenetics, Inflammation, Immunological deficiency syndromes, Ubiquitylation, Immunopathogenesis

## Abstract

The pathogenic mechanisms underlying pyoderma gangrenosum (PG) remain unclear. Here we report three patients with PG from two unrelated kindreds with homozygous R57C mutation of the linear deubiquitinase OTULIN. The patients have isolated, pediatric-onset, OTULIN-related PG (ORP). In contrast to OTULIN-related autoinflammatory syndrome (ORAS), caused by mutations affecting the catalytic domain, R57C affects the PUB-interacting motif with distinct biochemical, immunological and clinical consequences. OTULIN-R57C is catalytically active but is unable to bind the linear ubiquitin assembly complex (LUBAC). Patient monocytes show heightened expression of *IL1B*, and OTULIN-R57C fails to suppress inflammasome activity. Patients have elevated levels of TNF, and their dermal fibroblasts show heightened susceptibility to TNF-dependent cell death. Homozygosity for OTULIN-R57C leads to accumulation of linear ubiquitin and LUBAC autoubiquitination in patients’ dermal fibroblasts, consistent with pathogenic LUBAC activity. These findings identify a genetic etiology of isolated PG of childhood. We propose a multifactorial mechanism of ORP, including myeloid IL-1β production and TNF-driven death of skin-resident cells, suggesting that blockade of IL-1β or TNF are therapeutic options in PG.

## Abstract (web summary)

Some patients with pyoderma gangrenosum harbor a mutation (R57C) in OTULIN that disrupts the interaction between OTULIN and LUBAC leading to hyperactivation in monocytes and enhanced susceptibility of keratinocytes to TNF-induced, caspase-dependent cell death. One patient treated with anti-TNF therapy showed clinical remission of skin ulceration.

## Main

Inborn errors of immunity (IEIs) are a group of monogenic diseases that lead to dysregulation of immune development and/or function^1,2^. Identifying and characterizing new IEIs creates opportunities to dissect molecular and cellular mechanisms of human diseases and may point to potential therapeutic targets. Pyoderma gangrenosum (PG) is a rare, severe and poorly understood inflammatory skin disease with a population prevalence estimated between 0.03% and 0.05% (refs. ^3,4^) characterized by painful ulcerative lesions, a high burden of morbidity and, rarely, mortality^5–8^. While PG primarily affects adults, approximately 4% of patients are infants and children^9,10^. Currently, no PG-specific therapies are available, and conventional treatments, including corticosteroids, immunosuppressants and antimicrobials, yield variable results^11–20^. The absence of broadly effective therapies underscores an incomplete understanding of the pathophysiological mechanisms of PG, and the inter-individual variability in treatment response implies potential heterogeneity in disease mechanisms.

PG can present in isolation or in conjunction with other inflammatory diseases^21–23^. Certain IEIs, including pyogenic arthritis, PG and acne (PAPA) syndrome, and PG, acne and suppurative hidradenitis (PASH) syndrome include PG as a clinical feature alongside other inflammatory phenotypes and are caused by gain-of-function mutations in *PSTPIP1*, a positive regulator of pyrin inflammasomes^24–27^. Specific mutations affecting the N terminus of pyrin (encoded by *MEFV*) disrupt pyrin inhibition and also cause PG alongside systemic autoinflammatory symptoms in a disease termed pyrin-associated autoinflammation with neutrophilic dermatosis (PAAND)^28,29^; however, *PSTPIP1* and *MEFV* mutations explain only a small percentage of PG cases and are not known to cause isolated PG without systemic inflammation. In cases of isolated PG without other, syndromic features, there is evidence of familial clustering^30^, but its genetic basis is poorly understood. These findings prompt a search for as-yet unknown genetic etiologies of isolated PG in humans.

## Results

### A homozygous R57C mutation in *OTULIN* in patients with isolated PG

**Fig. 1:**
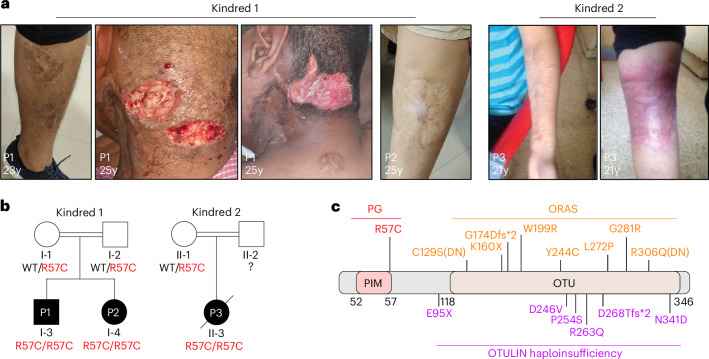
Bi-allelic OTULIN mutation R57C is identified in three patients from two unrelated kindreds with recurrent, isolated pyoderma gangrenosum of childhood. **a**, Images of PG skin lesions and resultant scarring on three patients (P1, P2 and P3) at the indicated ages. **b**, Pedigrees of two unrelated consanguineous kindreds (Kindred 1 and Kindred 2) with a total of three individuals affected by PG (P1, P2 and P3 – filled symbols) showing familial segregation of the OTULIN-R57C allele. **c**, Schematic diagram of OTULIN indicating the PIM and OTU-domain. All characterized IEI-causal OTULIN mutations are notated. Mutations that cause ORAS in the bi-allelic or mono-allelic (DN) state are shown in orange. Heterozygous mutations that cause OTULIN haploinsufficiency are shown in purple.

**Extended Data Fig. 1:**
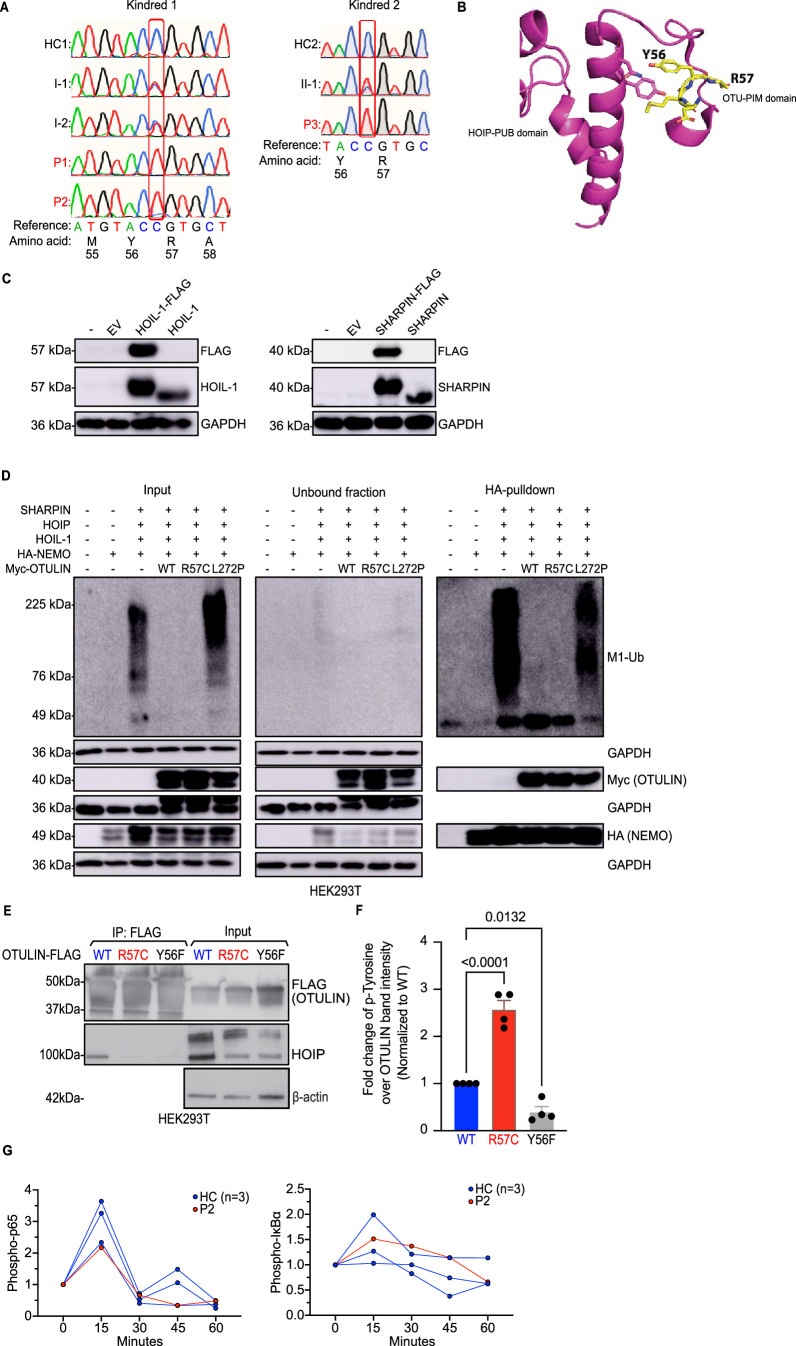
Confirmation of bi-allelic OTULIN mutations in three patients. **A**) Sanger sequencing of gDNA from all affected individuals and a total of three parents in Kindreds 1 and 2, and healthy controls (HC1-2) surrounding the R57C mutation in OTULIN. **B**) Modeling of interaction sites of the OTULIN PIM domain and the HOIP PUB domain, based on PDB entry 4OYK. **C**) Left panel: Whole cell lysates from HEK293T cells left untransfected, or transfected with empty vector (EV), HOIL1-FLAG or HOIL1 (untagged) plasmids were used to confirm ablation of the FLAG tag. Right panel: Whole cell lysates from HEK293T cells left untransfected, or transfected with empty vector (EV), SHARPIN-FLAG or SHARPIN (untagged) plasmids were used to confirm ablation of the FLAG tag. **D**) Assessment of linear ubiquitin (M1-Ub) accumulation in cells ectopically expressing HA-tagged NEMO, LUBAC components, and Myc-tagged OTULIN in WT or mutant forms, as indicated, in whole cell lysates (Input), an HA-immunoprecipitated fraction (HA-pulldown), or in the unbound protein fraction. Data are representative of one experiment from three independent replicates. **E**) Co-immunoprecipitation of endogenous HOIP in HEK293T cells transiently transfected with plasmids encoding FLAG-tagged OTULIN in WT or mutant forms, as indicated. **F**) Phospho-tyrosine band intensities relative to OTULIN intensities following FLAG-immunoprecipitation of cells ectopically expressing FLAG-tagged OTULIN in WT or mutant forms. Data from three independent replicates, mean and SEM per group, statistical analysis by one-way ANOVA with Dunnett multiple comparison correction. **G**) Quantitation of normalized signal for phosphorylated p65 and phosphorylated IκBα from experiments performed as in B-LCL from healthy controls (n = 3) and P2 stimulated with TNF for the indicated times. Signals at times 15, 30, 45 and 60 min were normalized to the signal at time 0 min for each individual and plotted. Source data

To test the hypothesis that isolated PG of childhood may result from an underlying yet unrecognized IEI, we studied patients with severe and spontaneous recurrent PG and without mutations in *PSTPIP1*. We identified three patients from two unrelated families who shared a homozygous mutation in *OTULIN*. Patient 1 and Patient 2 (P1, P2) from Kindred 1, a third-degree consanguineous family of South Asian ancestry, experienced recurrent PG since childhood without symptoms of systemic autoinflammation or heightened susceptibility to infections (Fig. 1a and Supplementary Materials). Whole-exome sequencing (WES) of genomic DNA from the two patients and healthy parents of Kindred 1 revealed a mutation in *OTULIN* (NM_138348.6) resulting in a missense p.Arg57Cys (R57C) substitution (Fig. 1b). Patient 3 (P3) of Kindred 2 was born to third-degree consanguineous parents of admixed American ancestry (unrelated to Kindred 1) and presented with a severe neutrophilic dermatosis consistent with PG since childhood (Fig. 1a and Supplementary Materials). At the age of 27 years and following a disease flare, P3 died as a consequence of postoperative complications and iatrogenic kidney failure. WES revealed that P3 also had a homozygous R57C mutation in *OTULIN* (Fig. 1b). WES results were confirmed by Sanger sequencing (Extended Data Fig. 1A). The patients did not carry variants fitting the known modes of inheritance for any of the 507 other genes already implicated in IEIs^31^. The OTULIN-R57C allele is extremely rare, with a minor allele frequency (MAF) of 1.55 × 10^−5^ (the maximal MAF across different genetic ancestries being 3.7 × 10^−4^ in admixed American populations), and no homozygous carriers have been reported in public databases^32^. This variant has a combined annotation-dependent depletion-score of 27.5, predicting it within the top 0.2% of the most deleterious substitutions in the human genome^33^. Protein impact prediction analyses yielded a Polymorphism Phenotyping v2 (PolyPhen-2) maximal score of 1.0 and an AlphaMissense score of 0.615, both consistent with a predicted damaging effect on protein function^34,35^. These metrics, together with the allele’s absence in homozygosity in public databases, support the likely pathogenicity of this variant under recessive inheritance. Indeed, the OTULIN-R57C allele co-segregated with PG as an autosomal recessive trait in the two kindreds of distant ancestries, suggesting that it underlies a fully penetrant IEI that predisposes to pediatric-onset PG (Fig. 1b).

### The R57C mutation is located in the PIM of OTULIN

OTULIN is a deubiquitinase (DUB) specific for linear ubiquitin (M1-Ub) chains^36–39^. M1-Ub chains are assembled by the linear ubiquitination assembly complex (LUBAC), consisting of HOIP, HOIL1 and SHARPIN^38,40,41^. OTULIN harbors two major structural domains: the C-terminal ovarian tumor (OTU) domain, responsible for its catalytic function, and the N-terminal domain encompassing the PNGase/ubiquitin-associated (PUB)-interacting motif (PIM)^38^. By binding to the PUB domain of HOIP, the PIM is essential for recruitment of OTULIN to LUBAC^38,42^. Currently, three rare, molecularly distinct forms of OTULIN-related disorders have been reported in humans^43^, underscoring the critical role of M1-Ub in human immunity^44^. Bi-allelic OTULIN deficiency manifests as the systemic, neonatal-onset OTULIN-related autoinflammatory syndrome (ORAS)^45–49^. OTULIN haploinsufficiency is a dominant disorder predisposing to necrosis triggered by staphylococcal infections^50,51^. OTULIN deficiency resulting from dominant-negative mutations affecting specific catalytic residues exhibits partial phenotypic overlaps with ORAS and OTULIN haploinsufficiency^52,53^. All three forms of OTULIN deficiency are caused by de novo or ultra rare mutations in the OTU-domain, affecting protein expression or catalytic activity of OTULIN. In contrast, the OTULIN-R57C allele, found in patients with PG, locates in the PIM (Fig. 1c and Extended Data Fig. 1B). Given the distinct clinical phenotype of our patients relative to other IEI caused by OTULIN, and their mutation affecting the PIM instead of the OTU-domain, we set out to characterize functionally the OTULIN-R57C allele.

### OTULIN-R57C does not directly impact its capacity to regulate NF-κB signaling

**Fig. 2:**
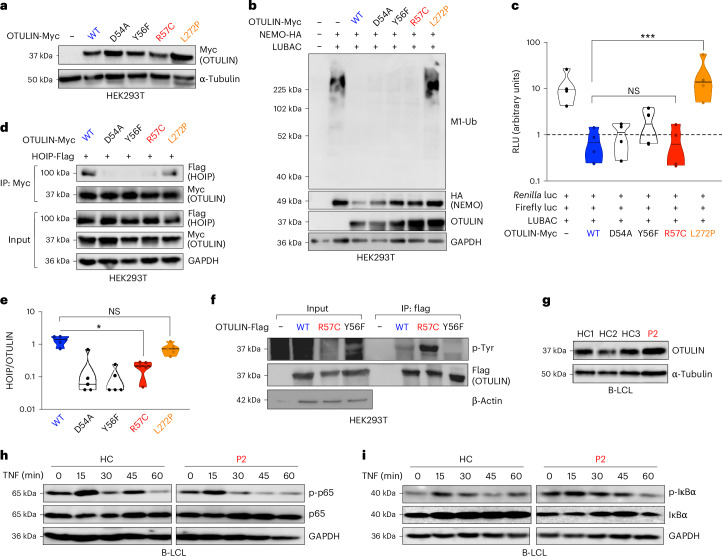
OTULIN-R57C abrogates binding to HOIP but retains deubiquitinase activity. **a**, Expression of OTULIN in HEK293T cells transiently transfected with plasmids encoding Myc-tagged OTULIN in WT or mutant forms, as indicated. **b**, Assessment of linear ubiquitin (M1-Ub) accumulation in cells ectopically expressing HA-tagged NEMO, LUBAC components and Myc-tagged OTULIN in WT or mutant forms, as indicated. **c**, NF-κB promotor activity as measured by dual luciferase assay in HEK293T cells expressing LUBAC components and Myc-tagged OTULIN in WT or mutant forms, as indicated. ***P < 0.0001, two-tailed student’s *t*-test. **d**, Co-immunoprecipitation of OTULIN-Myc in WT and mutant forms, as indicated, with HOIP–Flag. **e**, Quantification of HOIP/OTULIN binding ratios from five independent experiments performed as in **d**, *P = 0.0079, two-tailed student’s *t*-test. Horizontal line across each violin indicates median. **f**, Assessment of phospho-tyrosines (p-Tyr) following stimulation with TNF and MG-132 by means of Flag-immunoprecipitation in cells ectopically expressing Flag-tagged OTULIN in WT or mutant forms, as indicated. Data are representative of one experiment from three independent replicates. **g**, Endogenous expression of OTULIN in protein lysates of B-lymphocytic cell lines (B-LCL) from three healthy controls (HCs) (HC1–HC3) and P2. **h**, Assessment of phosphorylated (p-p65) and total p65 in whole-cell lysates of B-LCL from HCs and P2 B-LCL following stimulation with TNF for the indicated times (in minutes). Data are representative of one experiment from three independent replicates. **i**, Phosphorylated (p-IκBα) and total IκBα in whole-cell lysates of B-LCL following TNF stimulation, as in **h**. Data are representative of one experiment from three independent replicates. Source data

M1-Ub chains are post-translational modifications that regulate the activity of several signaling pathways and are generated exclusively by LUBAC^40,41^. Via binding to HOIP, OTULIN regulates LUBAC function by preventing its autoubiquitination^54^. Therefore, maintaining a balance between OTULIN’s DUB and LUBAC-associated activities is critical for regulating M1-Ub-dependent signaling^55^. Canonical NF-κB signaling is a well-characterized pathway that is controlled by OTULIN and LUBAC^42,56–60^. Unlike patients with ORAS, the patients reported here did not present with clinically overt systemic inflammation. Thus, we hypothesized that ectopic expression of wild-type (WT) or R57C OTULIN would efficiently repress NF-κB signaling, while the catalytically inactive L272P allele would not^45,46^. We tested this by first assessing M1-Ub levels and NF-κB-dependent transcription in cells ectopically expressing LUBAC, NEMO and either WT or mutant OTULIN. The R57C mutation did not alter the abundance of OTULIN protein in ectopic expression experiments (Fig. 2a). In contrast to the ORAS-causing mutation L272P^45,46^, and like WT OTULIN, the R57C allele and control mutants in the PIM all suppressed M1-Ub accumulation (Fig. 2b) and removed M1-Ub from NEMO (Extended Data Fig. 1C,D). OTULIN-R57C also maintained NF-κB regulating activity when ectopically expressed (Fig. 2c). Thus, unlike OTU-domain mutations, ectopically expressed OTULIN-R57C retains its catalytic activity and its capacity to regulate inflammatory NF-κB signaling.

### Mutations within the OTULIN PIM inhibit binding to HOIP

The divergent functional consequences of mutations in the PIM versus the OTU-domain of OTULIN suggest a molecular basis for the distinct clinical phenotypes caused by bi-allelic OTULIN mutations in patients with PG versus ORAS. To differentiate the impact of PIM versus OTU-domain mutations, we studied the impact of these mutations on OTULIN-HOIP binding. We included PIM mutants D54A and Y56F, previously shown to disrupt OTULIN-HOIP binding^38^, as biochemical controls, and the catalytically inactive L272P allele in the OTU-domain^45,46^. By co-immunoprecipitation, we found that HOIP was bound to both WT and L272P OTULIN, as expected. In contrast, all OTULIN PIM mutations, including R57C, showed impaired binding to HOIP (Fig. 2d, e). This impaired binding was also observed for HOIP endogenously, rather than ectopically expressed (Extended Data Fig. 1E). When OTULIN is phosphorylated at residue Y56, it cannot bind HOIP^37,38,42,44^. We hypothesized that the patients’ allele, affecting the residue directly C-terminal of Y56, impacts the phosphorylation status of OTULIN. Immunoprecipitation of ectopically expressed OTULIN-R57C yielded an increased signal for phosphorylated tyrosines at the molecular weight (MW) corresponding with OTULIN, when compared to cells expressing the WT allele or the phosphorylation-deficient mutant Y56F (Fig. 2f and Extended Data Fig. 1F)^37,38,43,44^. These data suggest that increased phosphorylation of OTULIN-R57C is one molecular mechanism for its incapacity to bind HOIP. Thus, despite displaying conserved DUB activity, the OTULIN-R57C allele impairs binding to LUBAC. As all currently known disease-causing mutations cluster within the OTU-domain, while the PG-causing R57C mutation localizes to the PIM (Fig. 1c), our results indicate that mutations in different OTULIN domains can lead to clinically distinct IEI with molecularly uncoupled mechanisms of disease. Therefore, we next sought to better understand the molecular and cellular drivers of these mutation-specific disease states.

### Immunological characterization of the patients’ PBMCs

**Extended Data Fig. 2:**
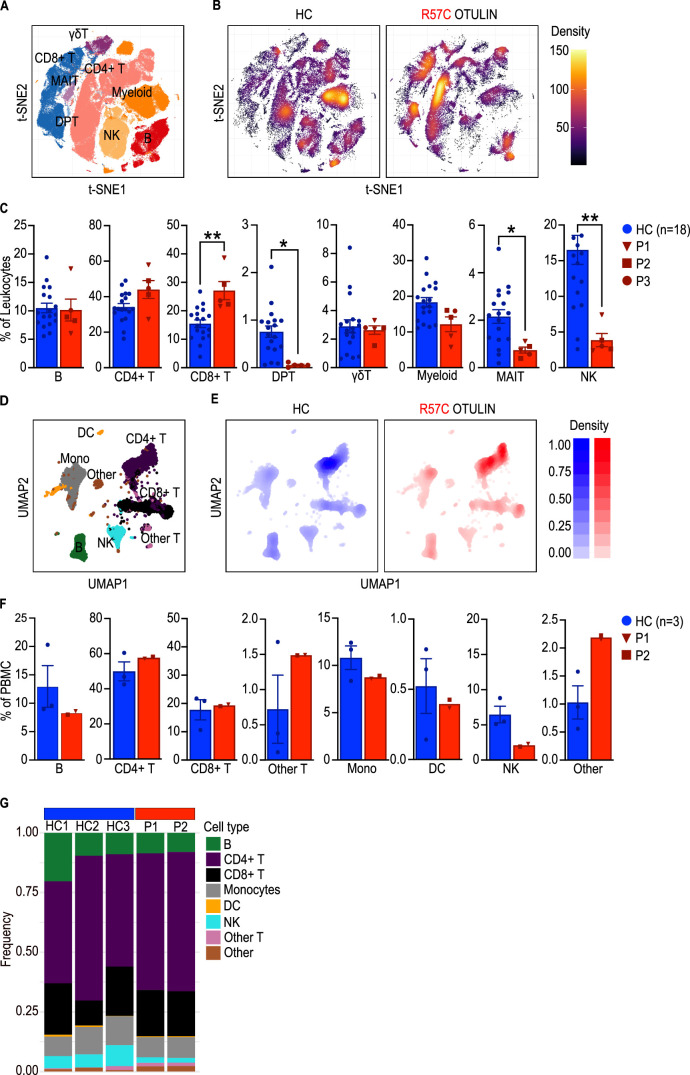
Immune subset frequencies are largely normal in PG patients with OTULIN-R57C homozygosity. **A**) T-distributed stochastic neighbor embedding (t-SNE) plots of data generating using of 33 metal-conjugated antibodies and cytometry by time of flight (CyTOF) using healthy control and patient PBMC samples. A total of 9,500 cells from each healthy control (HC, n = 18 total) and OTULIN-R57C patient (n = 5 samples, generated from two blood samples each from P1 and P2 drawn at different times, and one sample from P3) are included in the t-SNE. **B**) Density t-SNE showing the distribution of leukocytes in healthy controls (HC) and patients in CyTOF analysis. A total of 19,000 cells are represented in each t-SNE plot. **C**) Immune subset frequencies were calculated using PBMC samples and CyTOF analysis of HC (n = 18) and OTULIN-R57C (n = 5 samples, generated from two blood samples each from P1 and P2 drawn at different times, and one sample from P3). **D**) Single-cell RNA sequencing data from HC (n = 3), P1, and P2 were mapped to CITE-Seq reference dataset of healthy human PBMCs (see Materials and Methods for details). Each color represents a cell type cluster. **E**) Density UMAP plots depicting ccRNA-seq data from HCs (blue) and OTULIN-R57C patients (red). Each UMAP plot depicts 31,885 cells. **F-G**) Immune subset frequencies of healthy controls (HC, n = 3) and OTULIN-R57C patients (P1, P2) determined by scRNA-seq. Data in **F**) are shown as mean ± SEM. Source data

Overt immunological disturbances of the development and function of leukocytes are absent in patients with OTULIN haploinsufficiency^50^. The autoinflammatory features seen in patients with ORAS and patients with dominant-negative OTULIN deficiency are attributed, in large part, to abnormally high levels of NF-κB activation in leukocytic cells^46,52^. OTULIN was normally expressed in OTULIN-R57C patient B-lymphocytic cells (B-LCL) (Fig. 2g), and these cells showed no marked differences in the TNF-dependent activation of p65 and IκBα, signaling components of the canonical NF-κB pathway (Fig. 2h,i and Extended Data Fig. 1G). These data indicate that, in addition to contrasting in terms of impact on DUB activity, ORAS-causal mutations in *OTULIN* and OTULIN-R57C differ in terms of their impacts on NF-κB signaling in leukocytes. To detect other potential impacts of OTULIN-R57C on leukocyte frequencies and functions, we employed multiple unbiased approaches. Clinical immunophenotyping from P1 and P2 showed no major abnormalities in total T, B or natural killer (NK) cell frequencies (Extended Data Table 1). To more comprehensively investigate potential effects of OTULIN-R57C on immune cell subsets, we performed cytometry by time of flight (CyTOF) using peripheral blood mononuclear cells (PBMCs) from patients and healthy controls, identifying eight major leukocyte subsets (Extended Data Fig. 2A). T-distributed stochastic neighbor embedding (*t*-SNE) visualization suggested subtly altered frequencies of some subsets in the patients (Extended Data Fig. 2B). Indeed, while many subsets were unchanged, frequencies of double-positive T (DPT), mucosal-associated invariant T (MAIT) and NK cells were modestly lower, but within the range of healthy controls, in patients (Extended Data Fig. 2C). While many IEIs are associated with reduced frequencies of specific immune subsets including MAIT and NK cells such as loss-of-function mutations in *DOCK8* and *BCL10* (refs. ^61,62^), these conditions present with a variety of clinical manifestations that do not include PG. Further, higher proportions of DPT cells, rather than reduced, are associated with inflammation and IEIs^63–65^. Thus, we did not identify any changes in leukocyte subset frequencies that pointed to specific mechanisms of PG pathogenesis in these patients. In addition, analysis of single-cell RNA sequencing (scRNA-seq) data identified eight major leukocyte subsets with no overt frequency differences between patients and controls (Extended Data Fig. 2D–G). These results suggest that the OTULIN-R57C mutation may drive PG pathogenesis due, in part, to altered leukocyte functions, but not frequencies.

### Overlapping and distinct transcriptional patterns in patients with PG and ORAS

**Extended Data Fig. 3:**
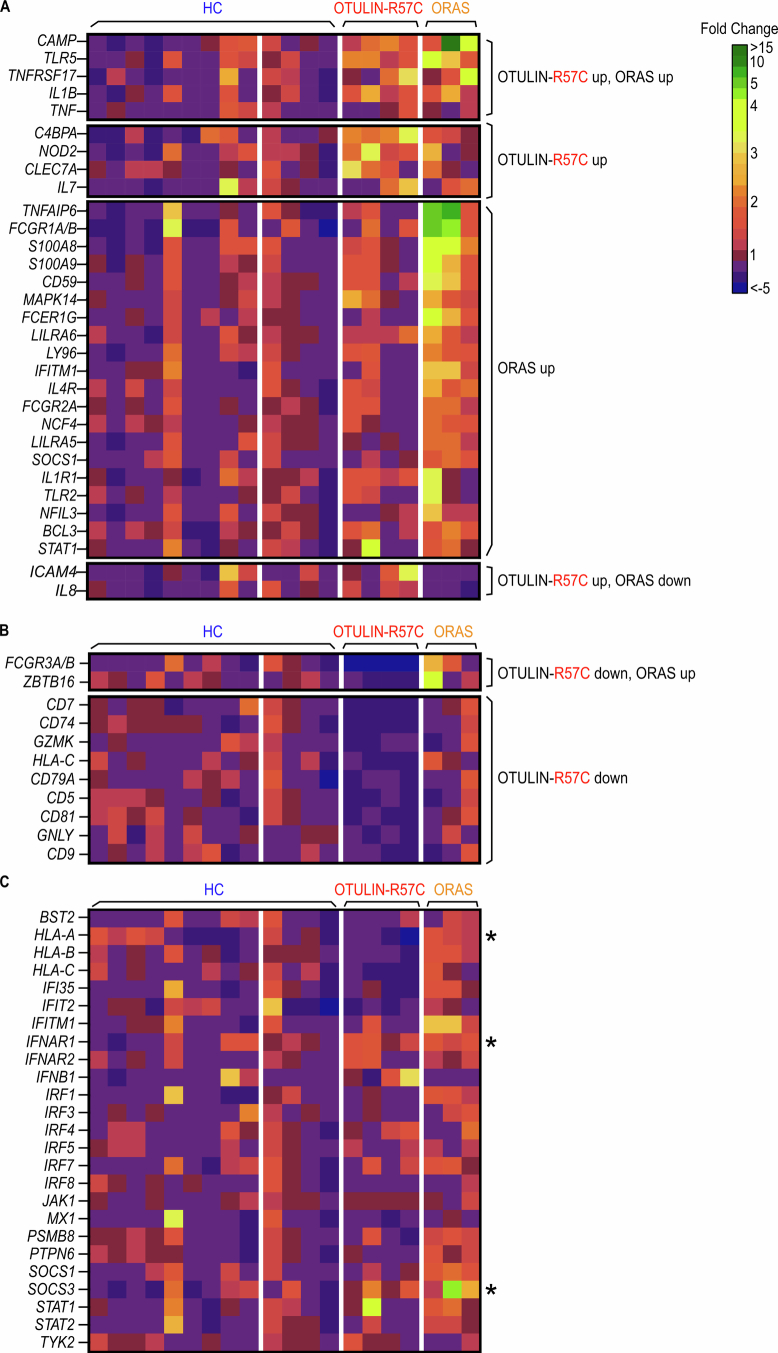
Gene expression analysis reveals both overlapping and distinct inflammatory signatures in whole blood samples from ORAS and OTULIN-R57C patients. **A**) Heatmap showing relative expression of genes comparing P1 and P2 (two blood draws for each patient) with healthy controls, and three ORAS patients. Gene expression was quantified by nCounter Immunology Panel as described in the Materials and Methods. Genes that showed differential expression (p < 0.05, two-tailed student’s t-test comparing means of patient vs. control groups) and fold change |>1.9| were included in the heatmap. **B**) Heatmap showing relative expression of genes comparing P1 and P2 (two blood draws for each patient) with healthy controls, and three ORAS patients. Gene expression was quantified by nCounter Immunology Panel as described in the Materials and Methods. Genes that showed differential expression (p < 0.05, two-tailed student’s t-test comparing means of patient vs. control groups) and fold change |>1.9| were included in the heatmap. **C**) Heatmap showing relative expression of interferon-stimulated genes (ISGs) comparing P1 and P2 (two blood draws for each patient) with healthy controls, and three ORAS patients. Gene expression was quantified by nCounter Immunology Panel as described in the Materials and Methods. *p < 0.05, two-tailed student’s t-test comparing ORAS to HC samples.

As a first step to understand how *OTULIN* mutations may perturb leukocyte functions, we used a probe-based messenger RNA expression platform to broadly assess the expression of immune-related genes in whole blood from OTULIN-R57C patients and compared them to healthy controls. In parallel, we obtained comparable gene expression data comparing whole blood from patients with ORAS to a second set of healthy controls^46^. We identified shared upregulated cytokine-encoding genes in OTULIN-R57C patients and patients with ORAS such as *IL1B*, which recapitulates others’ findings of IL-1β hyperproduction in both PG and ORAS (Extended Data Fig. 3A)^45,46,66,67^, and *TNF*, a major contributor to ORAS pathogenesis^45,47,49,52,53^ which is also elevated in blood and lesions from some patients with PG^12,68–70^. In line with the greater severity of ORAS compared to PG, patients with ORAS showed extensive transcriptional changes, whereas patients with PG displayed a more restricted inflammatory signature. Of note, some genes such as *IL8* (a neutrophil chemoattractant elevated in PG patient skin lesions^68,71^) were upregulated in PG but downregulated in ORAS, or vice versa (Extended Data Fig. 3A, B). OTULIN has been implicated in the negative regulation of type I interferon (IFN) responses due to proteasome dysregulation, demonstrated by catalytically inactive OTULIN mutations leading to high expression of IFN-stimulated genes (ISGs) in PBMCs and serum samples from patients with ORAS^72^. Expression of various truncated versions of OTULIN demonstrated that regulation of ISG expression relied on the OTU-domain rather than the PIM^72^. In agreement with these data, patients with ORAS showed elevated expression of several ISGs relative to healthy controls, but P1 and P2 did not (Extended Data Fig. 3C). Consistent with similar TNF-dependent activation of p65 and IκBα in patient and healthy control B-LCL cells (Fig. 2h,i and Extended Data Fig. 1G), OTULIN-R57C patient samples did not display elevated expression of several canonical NF-κB signaling molecules or target genes (Extended Data Table 2), further underscoring the unique pathophysiology of the R57C mutation. Collectively, these data suggest both overlapping and distinct disease mechanisms that control OTULIN mutation-driven ORAS versus PG.

### Elevated expression of cytokines in monocytes from OTULIN-R57C patients

**Fig. 3:**
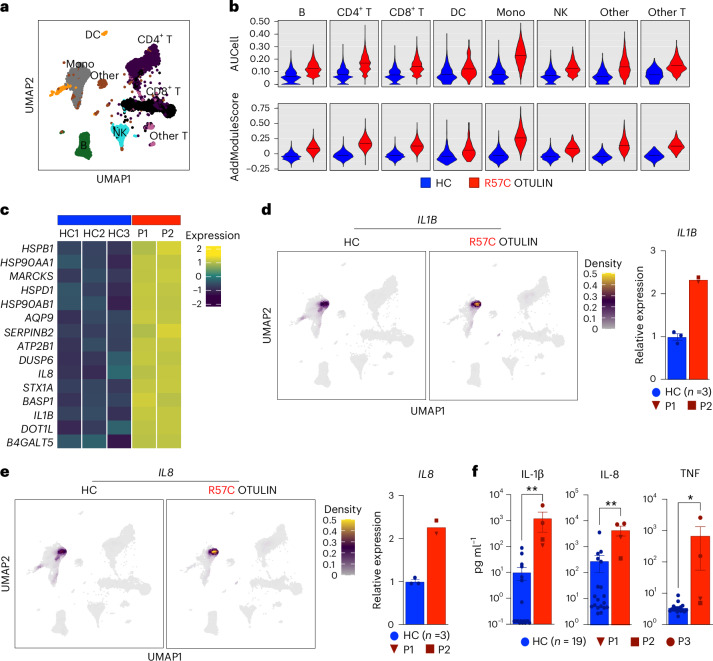
OTULIN-R57C patients’ monocytes show elevated expression of inflammatory cytokines. **a**, scRNA-seq was performed on PBMC samples from HCs (*n* = 3, HC1–HC3), P1 and P2. Uniform Manifold Approximation and Projection (UMAP) was generated by mapping HC and patient data to a reference CITE-seq dataset of healthy human PBMCs (Methods provide details). Cell clusters are annotated by color. **b**, DEGs were identified, comparing samples from OTULIN-R57C patients to HCs. The top 15 DEGs genes were used as the input for scGSA. AUCell and AddModuleScore values were calculated for all eight immune subsets identified in **a**, identifying monocytes as the greatest contributors to the top DEGs (Methods provide details). Horizontal lines across each violin indicates median. **c**, Within monocytes, differential expression analysis was performed to identify DEGs comparing OTULIN-R57C patients’ samples to HC samples. Relative expression for the top 15 DEGs are represented by *z*-scores. **d**,**e**, Feature density UMAP plots showing expression of *IL1B* (**d**) and *IL8* (**e**); 31,885 cells are represented in each UMAP. Density is represented as a weighted kernel density estimation in UMAP and relative expression values for *IL1B* (**d**) and *IL8* (**e**) in monocytes are shown in bar graphs. **f**, Concentration of IL-1β, IL-8 and TNF in plasma samples from HCs (*n* = 19) and OTULIN-R57C patients (one blood draw from each of P1 and P2, two independent blood draws from P3; total *n* = 4 datapoints) were determined by multiplex electrochemiluminescent immunoassays. **P = 0.0031 for IL-1β, **P = 0.0001 for IL-8, *P = 0.0168, two-tailed student’s *t*-test. Data are shown as mean ± s.e.m. Source data

**Extended Data Fig. 4:**
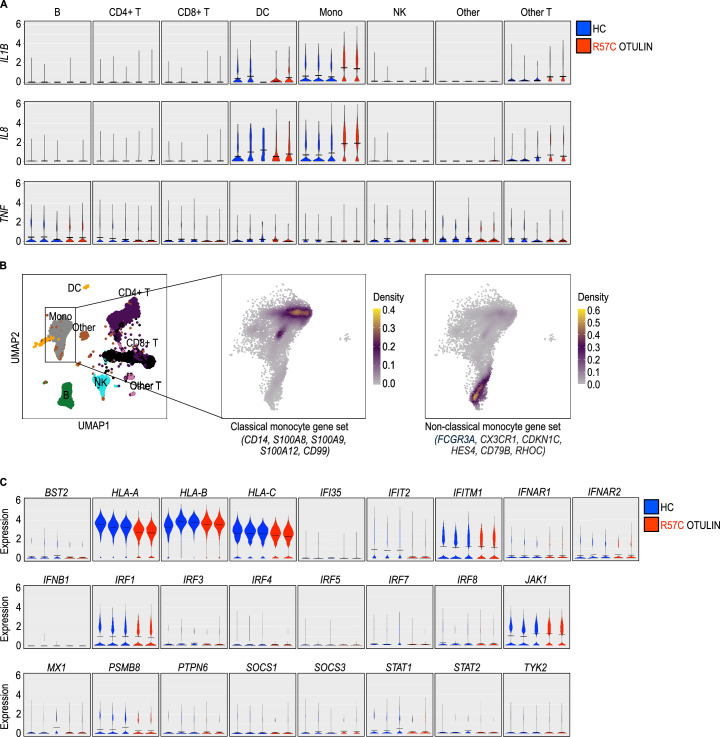
scRNA-seq analysis identifies high expression of inflammatory cytokines in monocytes of patients with OTULIN-R57C homozygosity. **A**) Normalized expression of IL1B, IL8, and TNF in healthy control (HC) and OTULIN-R57C patients’ samples for each indicated cell population identified in scRNA-seq dataset. **B**) Single-cell RNA sequencing was performed on PBMC samples from healthy controls (n = 3, HC1-3), P1, and P2. UMAPs were generated by mapping healthy control and patient data to a reference CITE-seq dataset of healthy human PBMCs (refer to Materials and Methods for details). Cell clusters are annotated by color, as in Fig. 4a. Indicated marker gene sets were used to identify classical and non-classical monocytes and were visualized by feature density UMAP plots. **C**) Normalized expression of indicated interferon-stimulated genes (ISGs) in healthy control (HC) and OTULIN-R57C patients’ samples by scRNA-seq. Horizontal line across each violin indicates median.

Next, we aimed to elucidate which cell type(s) were driving the transcriptomic changes identified in the whole blood of OTULIN-R57C patients. Using scRNA-seq, we classified eight major leukocyte subsets (B, CD4^+^ T, CD8^+^ T, DC, monocytes, NK, other and other T) in the PBMC samples of patients and healthy controls (Fig. 3a). We identified 985 differentially expressed genes (DEGs) by comparing all eight cell subsets from OTULIN-R57C patients to those of healthy controls. The top 15 DEGs were used in two independent single-cell gene set analysis (scGSA) methods, AUCell^73^ and AddModuleScore^74^. Both approaches identified monocytes as the primary contributors to OTULIN-R57C-associated transcriptomic changes (Fig. 3b). We found that five of the top DEGs from PBMCs were monocyte-specific, reinforcing that monocytes play a central role in the OTULIN-R57C transcriptome (Fig. 3c and Extended Data Table 3). Among these DEGs were the inflammatory cytokines *IL1B* and *IL8* (Fig. 3d,e), which were selectively upregulated in patients’ monocytes (Extended Data Fig. 4A) and overlapped with genes enriched in classical monocytes (Extended Data Fig. 4B)^75^. Plasma levels of IL-1β and IL-8 were significantly elevated in OTULIN-R57C patients, consistent with both scRNA-seq and probe-based transcriptional findings (Fig. 3f). We also found that plasma from OTULIN-R57C patients with PG contained high levels of TNF (Fig. 3f) in agreement with its elevated mRNA expression in whole blood (Extended Data Fig. 3A), but the source of its hyperproduction could not be traced back to any cell type detected in our PBMC-based scRNA-seq data (Extended Data Fig. 4A). In agreement with our findings in whole blood, we did not observe heightened expression of ISGs in OTULIN-R57C patients’ PBMCs by scRNA-seq (Extended Data Fig. 4C). Taken together, these data suggest that OTULIN-R57C patients have hyperinflammatory monocytes, which may contribute to PG pathogenesis in these patients.

### OTULIN-R57C fails to suppress inflammasome activity

**Fig. 4:**
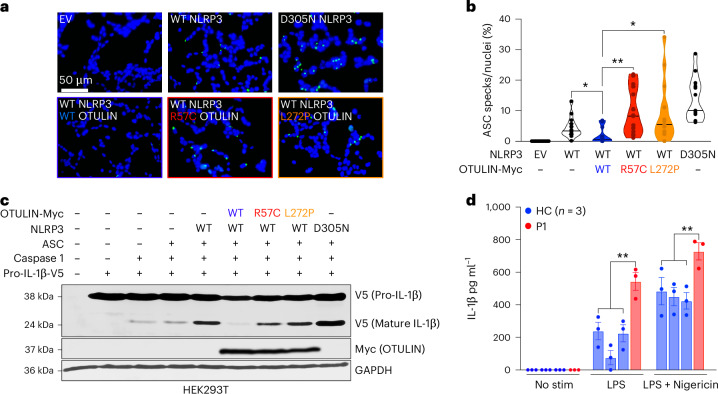
OTULIN-R57C fails to suppress inflammasome activity. **a**, ASC specks visualized in HEK293ASC-YFP cells ectopically expressing empty vector (EV), WT or D305N mutant forms of NLRP3, with or without co-transfection of WT or mutant OTULIN-Myc constructs, as indicated. Cells were imaged at ×40 magnification by fluorescence microscopy. Blue, 4,6-diamidino-2-phenylindole (DAPI); green, ASC specks. Data are representative of one experiment from three independent replicates. **b**, ASC specks per nuclei were calculated for each image captured (*n* = 15 images per condition) using ImageJ software. Data are representative of one experiment from three independent replicates. Horizontal lines across each violin indicates median. *P = 0.039 for comparison of WT NLRP3 with versus without WT OTULIN conditions, *P = 0.019 for comparison of conditions with WT versus L272P OTULIN, **P = 0.0008 for comparison of conditions with WT versus R57C OTULIN, two-tailed student’s *t*-tests. **c**, IL-1β processing was assessed in HEK293T cells ectopically expressing NLRP3 inflammasome components (NLRP3, ASC, caspase-1, V5-tagged pro-IL-1β) and Myc-tagged WT or mutant forms of OTULIN, as indicated. Data are representative of one experiment from three independent replicates. **d**, IL-1β secretion was measured by ELISA in the supernatants of PBMCs from HCs (*n* = 3) or P1 that were either left unstimulated (No Stim) or following stimulation with LPS or LPS and nigericin, as indicated. Data are shown as mean ± s.e.m., with *n* = 3 technical replicates. **P = 0.0004 for LPS, **P = 0.0019 for lipopolysaccharide (LPS) + nigericin, two-tailed student’s *t*-test comparing P1 to three HCs. Source data

**Extended Data Fig. 5:**
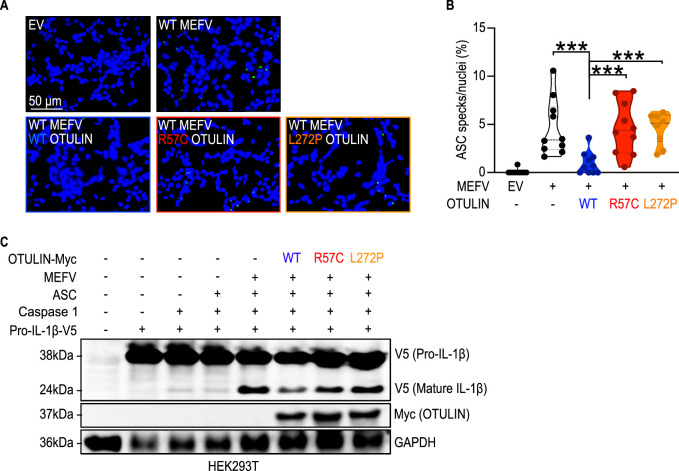
OTULIN-R57C fails to suppress inflammasome activity. **A**) ASC specks visualized in HEK293ASC-YFP cells ectopically expressing empty vector (EV), MEFV (encoding pyrin), with or without co-transfection of WT or mutant OTULIN-Myc constructs, as indicated. Cells were imaged at 40X magnification by fluorescence microscopy. Blue: DAPI, green: ASC specks. Data are representative of one experiment from 3 independent replicates. **B**) ASC specks per nuclei were calculated for each image captured (n = 8 or greater images per condition) using ImageJ software. Data are representative of one experiment from 3 independent replicates. Horizontal lines across each violin indicates median. ***p < 0.001 two-tailed stuent’s t-test. **C**) IL-1β processing was assessed in HEK293T cells ectopically expressing pyrin inflammasome components (MEFV, ASC, caspase-1, V5-tagged pro-IL-1β) and Myc-tagged WT or mutant forms of OTULIN, as indicated. Data are representative of one experiment from 3 independent replicates. Source data

We then sought to identify the mechanistic consequences of the OTULIN-R57C mutation that drives the hyperinflammatory state of patients’ monocytes and the hyperproduction of inflammatory cytokines. The NLRP3 and pyrin inflammasomes are protein complexes that rely on M1-Ub to mediate the cleavage of pro-IL-1β to mature IL-1β by caspase-1 (refs. ^76,77^). Murine models have demonstrated that LUBAC is essential for ASC-dependent NLRP3 inflammasome activation independently of NF-κB^76^, and that Otulin suppresses IL-1β production^78^. High IL-1β secretion is seen in patients with ORAS or gain-of-function (GOF) mutations in pyrin inflammasome adaptor *PSTPIP1* (refs. ^25,46,79^). Patients with PG (with no known underlying genetic basis) often also exhibit high IL-1β production in both blood and lesional tissue^20,66,67,80–82^. Given that OTULIN-R57C patients’ monocytes express high levels of IL-1β (Fig. 3c,d and Extended Data Fig. 4A), we hypothesized that excessive inflammasome activity is involved in the pathogenesis of these patients’ PG. To test this, we assessed NLRP3 inflammasome-dependent ASC speck formation in cells ectopically expressing ASC, WT NLRP3 and WT or mutant OTULIN (Fig. 4a,b). As a positive control, we included the GOF NLRP3-D305N variant^83^. We found that WT or NLRP3-D305N induced ASC speck formation as expected, and WT OTULIN reduced NLRP3-dependent specks. In contrast, the PG-causing R57C and ORAS-causing L272P variants failed to negatively regulate ASC speck formation (Fig. 4a,b). As an orthogonal approach, we tested whether the OTULIN variants could suppress NLRP3-dependent IL-1β cleavage. As expected, WT or NLRP3-D305N led to cleavage of pro-IL-1β to mature IL-1β, and this was abrogated by WT OTULIN (Fig. 4c). Consistent with our ASC speck results, R57C and L272P OTULIN variants failed to suppress IL-1β cleavage (Fig. 4c). Similar experiments indicated that R57C and L272P mutant forms of OTULIN were also unable to suppress pyrin inflammasome activation (Extended Data Fig. 5). Consistent with our data in ectopic expression systems, we observed elevated IL-1β secretion by PBMCs from P1, relative to healthy controls, following exposure to NLRP3 inflammasome triggers (Fig. 4d). These data implicate dysregulated inflammasome activity as a contributor to the elevated IL-1β levels in our patients with PG, and possibly also in patients with ORAS^46,53^. Scattered case reports show that some but not all patients with PG who receive IL-1 inhibitors responded positively to treatment^19,20,81,84^ suggesting a potential contribution of IL-1β in PG, while also implying additional pathogenic mechanisms. The existence of other IEIs characterized by high IL-1β but without PG-like lesions, such as NOMID caused by GOF mutations in NLRP3 (refs. ^85,86^), indicate that elevated IL-1β alone is not sufficient to explain the unique skin pathology observed in PG. Thus, we explored how OTULIN-dependent molecular mechanisms relevant to skin homeostasis may be perturbed in the OTULIN-R57C patients.

### OTULIN-R57C mediates caspase-dependent TNF-driven dermal cell death

**Fig. 5:**
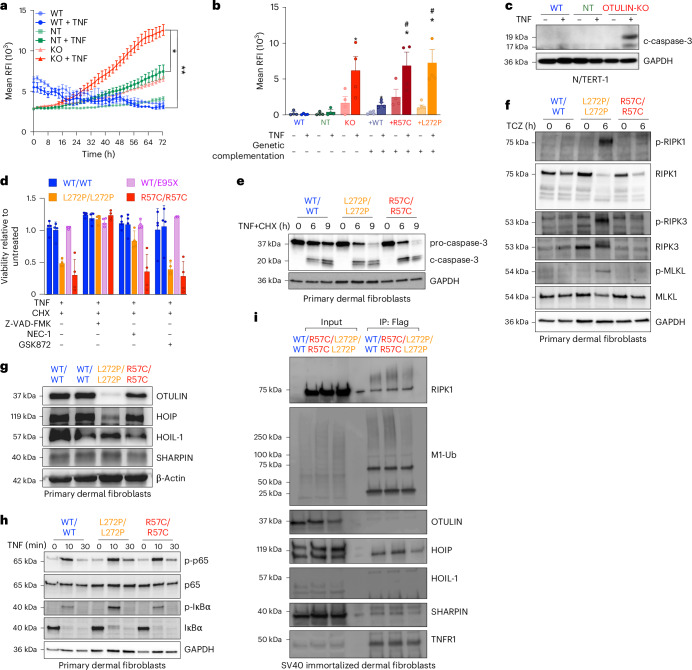
Genotype-specific consequences of OTULIN mutations on mechanisms of TNF-dependent of death in patient skin cells. **a**, WT, NT and KO N/TERT-1 keratinocyte cells were unstimulated or stimulated with 100 ng ml^−1^ TNF for 72 h in the presence of propidium iodide (PI). Fluorescence microscopy images were captured every 2 h and PI uptake, quantified as mean red fluorescence intensity (RFI), was used to measure cell death. Data are representative of one experiment from three independent replicates. Data are shown as mean ± s.e.m. **P < 0.005, two-tailed student’s *t*-tests comparing TNF-treated KO to TNF-treated NT or TNF-treated WT cells, respectively. **b**, OTULIN-KO N/TERT-1 keratinocytes were genetically complemented with WT, R57C or L272P forms of OTULIN resistant to CRISPR-Cas9-dependent cleavage. These cells were either left unstimulated or stimulated with 100 ng ml^−1^ TNF in the presence of PI and assessed for cell death, as in **a**. Mean RFI at for 72 h is shown for each cell line, with and without TNF, from *n* = 4 independent experiments. Data are shown as mean ± s.e.m. *P < 0.018, two-tailed student’s *t*-test relative to TNF-treated WT cells, ^#^*P* < 0.025, two-tailed student’s *t*-test relative to TNF-treated cells genetically complemented with WT OTULIN. **c**, Cleaved caspase-3 signal in whole-cell lysates of N/TERT-1 cells of the indicated genotypes, either left untreated or stimulated with TNF for 24 h. Data are representative of one experiment from three independent replicates. **d**, Cell viability of dermal fibroblasts from two HCs (WT/WT), a patient with ORAS (L272P/L272P), an OTULIN haploinsufficient patient (WT/E95X) and P3 (R57C/R57C) after stimulation with TNF and cycloheximide, with and without specific inhibitors. Bars indicate s.d., with *n* = 5 replicates per condition. Z-VAD-FMK, pan-caspase inhibitor; necrostatin-1 (NEC-1), RIPK1 inhibitor; GSK872, RIPK3 inhibitor. Data are shown as mean ± s.d. **e**, Caspase-3 processing was assessed in whole-cell lysates of primary dermal fibroblasts cells following stimulation with TNF and cycloheximide. Data are representative of one experiment from three independent replicates. **f**, Phosphorylation of RIPK1, RIPK3 and MLKL in whole-cell lysates of primary dermal fibroblasts cells following stimulation with TNF, cycloheximide and Z-VAD-FMK. Data are representative of one experiment from three independent replicates. **g**, Endogenous expression levels of OTULIN, HOIP, HOIL1 and SHARPIN in primary dermal fibroblasts from two HCs (WT/WT), a patient with ORAS (L272P/L272P) and P3 (R57C/R57C). Data are representative of one experiment from three independent replicates. **h**, Phosphorylation and degradation of p65 and IκBα in whole-cell lysates of primary dermal fibroblasts following stimulation with TNF. Data are representative of one experiment from three independent replicates. **i**, Analysis of the TNF receptor signaling complex by means of Flag-immunoprecipitation in SV40-immortalized dermal fibroblasts following stimulation with Flag–TNF. Data are representative of one experiment from two independent replicates. Source data

**Extended Data Fig. 6:**
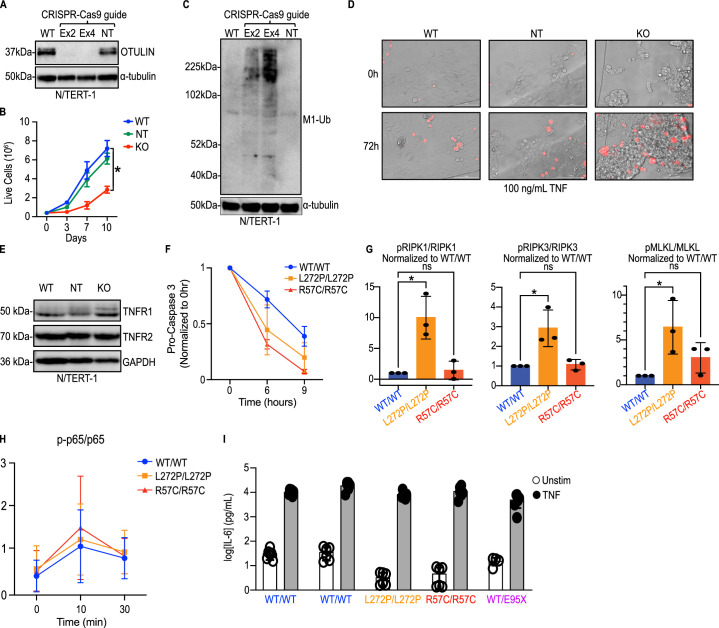
Genotype-specific consequences of OTULIN mutations on mechanisms of TNF-dependent of death in patient skin cells. **A,B**) Nontarget control (NT) and OTULIN-knockout (KO) N/TERT-1 human keratinocytes were generated via CRISPR-Cas9 (refer to Materials and Methods for details). Two different OTULIN-targeting gRNA were used, targeting OTULIN exon 2 (Ex2) and exon 4 (Ex4), respectively. Cells were lysed and subjected to immunoblotting against OTULIN (**A**) and linear ubiquitin (M1-Ub) (**C**). In subsequent panels, KO refers to cells generated using the OTULIN Ex4-targeting gRNA. **B**) Live keratinocytes were quantified over time using trypan blue exclusion and an automated cell counter. *p < 0.05, two-tailed student’s t-test at Day 10. **D**) Representative images of TNF-treated WT, NT, and KO cells were taken at 20X magnification, overlaying phase contrast and RFI signals, at indicating timepoints. **E**) The expression of TNF receptors 1 and 2 (TNFR1, TNFR2) were assessed by western blotting in WT, NT, and KO N/TERT-1 cells. **F**) Pro-caspase-3 band intensities relative to timepoint 0 in whole cell lysates of primary dermal fibroblasts cells following stimulation with TNF and cycloheximide. Data from three independent replicates, mean and SD per group. **G**) Phospho-RIPK1, -RIPK3, and -MLKL over total RIPK1, RIPK3, and MLKL band intensities, respectively, normalized against the mean value of healthy controls in whole cell lysates of primary dermal fibroblasts cells following stimulation with TNF, cycloheximide, and Z-VAD-FMK. Data from three independent replicates, mean and SD per group. *p < 0.05, one-way ANOVA with Sidak multiple comparison correction. **H**) Phospho-p65 over total p65 band intensities in whole cell lysates of primary dermal fibroblasts cells following stimulation with TNF. Data from three independent replicates, mean and SD per group. **I**) IL-6 secretion at baseline and following stimulation with 20 ng/mL TNF was measured in primary dermal fibroblasts from two healthy controls (WT/WT), an ORAS patient (L272P/L272P), an OTULIN haploinsufficient patient (WT/E95X) and P3 (R57C/R57C). Data are shown as mean ± SD with n = 6 replicates. Source data

Given our finding of elevated TNF in OTULIN-R57C patients’ blood (Fig. 3f and Extended Data Fig. 3A), the critical role of OTULIN in controlling TNF-dependent cell death^47,87^ and our patients’ severe yet skin-limited clinical phenotypes, we hypothesized that OTULIN is required to suppress TNF-driven cell death in their skin. Mice with keratinocyte-specific *Otulin* deficiency spontaneously develop skin lesions, dependent on signaling through the TNF receptor 1 (Tnfr1)^87,88^, but the exigency for OTULIN in human keratinocytes has not been demonstrated. To study the impact of OTULIN-R57C on dermal cell death, we generated OTULIN-knockout (KO) human keratinocytes and interrogated their responses to TNF. OTULIN-KO keratinocytes (Extended Data Fig. 6A) showed impaired growth relative to WT or nontarget (NT) control keratinocytes (Extended Data Fig. 6B) and accumulated M1-Ub (Extended Data Fig. 6C). OTULIN-KO keratinocytes were highly susceptible to TNF-induced cell death, relative to WT and NT cells (Fig. 5a, Extended Data Fig. 6D). Heightened susceptibility to TNF-dependent cell death was rescued by genetic complementation of OTULIN-KO keratinocytes with WT, but not the R57C or L272P mutant forms of OTULIN (Fig. 5b). We did not observe differential expression of either TNFR1 or TNFR2 when comparing WT, NT and OTULIN-KO cells (Extended Data Fig. 6E), indicating that downstream TNFR-signaling rather than TNFR expression was perturbed by OTULIN deficiency. Accordingly, activation of caspase-3, a marker of TNF-dependent and M1-Ub mediated keratinocyte death^87,89^, was increased in OTULIN-KO cells (Fig. 5c). These data point to an *OTULIN* genotype-dependent, TNF-driven and caspase-mediated form of skin cell death contributing to the pathogenesis in OTULIN-R57C patients with PG. A limitation of these experiments, however, is the use of complete genetic KO rather than endogenous OTULIN mutations. Thus, we sought to validate these findings using primary patient-derived cells, and to further probe the signaling events driving OTULIN-mediated susceptibility to TNF-induced cell death.

To test the impact of OTULIN-R57C on TNF-dependent responses in patients’ dermal cells, we exposed primary dermal fibroblasts from WT healthy controls, a patient with ORAS, an OTULIN haploinsufficient patient and P3 to TNF and cycloheximide (CHX). Like the OTULIN-KO keratinocytes (Fig. 5a,b) and the cells from the patient with ORAS, and in contrast to the OTULIN haploinsufficient patient’s cells^50^, cells from P3 were susceptible to TNF-CHX-induced cell death (Fig. 5d). The susceptibility of cells from P3 to TNF-CHX-induced cell death was mediated by the activation of caspases, as demonstrated by the accelerated cleavage of caspase-3 in cells from P3 when compared to cells from a healthy donor (Fig. 5e and Extended Data Fig. 6F) and the abrogation of TNF-CHX-induced cell death following pretreatment with the pan-caspase inhibitor Z-VAD-FMK (Fig. 5d). Pretreatment of cells with the RIPK3 inhibitor GSK872 did not protect cells from the ORAS patient or P3 (Fig. 5d). In contrast, pretreatment with the RIPK1 inhibitor necrostatin-1 rescued ORAS cells but not P3’s cells, indicating a differential involvement of RIPK1 in the mode of cell death (Fig. 5d). Indeed, upon stimulation with TNF-CHX and in the presence of Z-VAD-FMK, RIPK1, RIPK3 and MLKL were abundantly phosphorylated in the cells of the patient with ORAS but not in cells from P3 (Fig. 5f and Extended Data Fig. 6G). These observations suggest that OTULIN-R57C patient dermal fibroblasts die primarily of caspase-dependent apoptosis in response to TNF-CHX, while RIPK3- and MLKL-driven necroptosis also contributes to cell death in cells from patients with ORAS. Collectively, these data show that OTULIN is a critical regulator of TNF-dependent death in dermal cells, and indicate a pathophysiological role for TNF in OTULIN-R57C patients with PG.

### TNF receptor signaling in dermal cells from OTULIN-R57C patients

The molecular impacts of OTULIN deficiencies are cell-type and genotype dependent^45–47,50,52,53^. In patients with ORAS, bi-allelic OTULIN deficiency results in a compensatory loss of LUBAC expression in nonmyeloid cells^46,47,50^. In contrast, LUBAC is normally expressed in non-myeloid cells of patients with OTULIN haploinsufficiency^50^ and dominant-negative OTULIN deficiency^52,53^. As a first step to understand the mechanistic basis of differential TNF-dependent cell death in dermal fibroblasts from patients with ORAS versus OTULIN-R57C, we tested these cells for expression of OTULIN and LUBAC. Consistent with the results obtained in B-LCL cells from P2 (Fig. 2g), and differing from ORAS, the expression of OTULIN and LUBAC components were apparently normal in dermal fibroblasts from P3 (Fig. 5g). Next, we tested activation of p65 and IκBα in primary dermal fibroblasts at baseline and following stimulation with TNF. Relative to cells from a healthy control, a mild defect in NF-κB signaling was observed in cells from a patient with ORAS following stimulation with TNF, and no defect could be observed in cells from P3 (Fig. 5h and Extended Data Fig. 6H). Following exposure to TNF and as reported elsewhere in nonmyeloid cells of patients with ORAS^47,50^, dermal fibroblasts from P3 secreted IL-6 at comparable levels to healthy controls (Extended Data Fig. 6I). Thus, OTULIN-R57C retains its capacity to regulate inflammatory NF-κB signaling in response to TNF in patient’s skin cells. Collectively, our data demonstrate differential triggering of RIPK1- and NF-κB-dependent signaling in response to TNF in ORAS versus OTULIN-R57C patients’ cells, suggesting that events proximal to the TNF receptor may diverge in ORAS and OTULIN-R57C patients’ skin cells.

To further interrogate mechanisms of TNF-induced dermal cell death in OTULIN-R57C patients, we characterized the TNF receptor signaling complex (TNFR-SC) in patient skin-derived samples. We performed a Flag–TNF pulldown in dermal fibroblasts from a healthy donor, a patient with ORAS and P3. Consistent with findings in other patients with ORAS^47^ and with reduced presence of LUBAC at the TNFR-SC in ORAS, Flag–TNF stimulation yielded a decreased modification of the TNFR-SC with M1-Ub in the cells from the patient with ORAS when compared to cells from a healthy donor (Fig. 5i). In contrast, M1-Ub modification of the TNFR-SC in cells from P3 was highly abundant (Fig. 5i). Indeed, normal levels of LUBAC were recruited to the TNFR-SC in P3, relative to a healthy control (Fig. 5i). Irrespective of the genotype, OTULIN was not recruited to the TNFR-SC^50,51^ but RIPK1 was (Fig. 5i)^52^. Collectively, these data highlight OTULIN- and LUBAC-mediated differences of the TNFR-SC between bi-allelic OTULIN mutations affecting the OTU-domain and the PIM. Since M1-Ub is implicated in the regulation of TNFR-SC function^52,53^, the enhanced modification of the TNFR-SC with M1-Ub in OTULIN-R57C patients’ cells may impact susceptibility to TNF-dependent cell death in their skin.

### LUBAC is autoubiquitinated in dermal cells from OTULIN-R57C patients

**Fig. 6:**
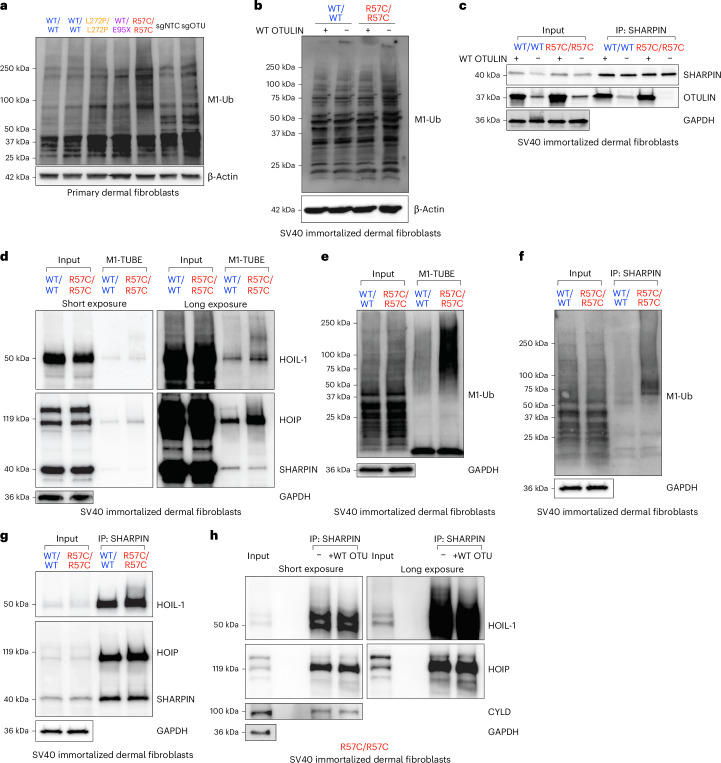
OTULIN-R57C patient dermal cells show accumulation of linear ubiquitin and aberrant LUBAC autoubiquitination. **a**, Linear ubiquitin (M1-Ub) levels in whole-cell lysates of primary dermal fibroblasts from two HCs (WT/WT), a patient with ORAS (L272P/L272P), an OTULIN haploinsufficient patient (WT/E95X) and P3 (R57C/R57C), in conjunction with the M1-Ub levels in whole-cell lysates of OTULIN-KO primary dermal fibroblasts (sgOTU) generated from an HC (sgNTC). Data are representative of one experiment from three independent replicates. **b**, M1-Ub levels in whole-cell lysates of SV40-immortalized dermal fibroblasts from an HC (WT/WT) and P3 (R57C/R57C) after rescue with WT OTULIN, as compared to cells rescued with an empty virus. Data are representative of one experiment from three independent replicates. **c**, Assessment of OTULIN binding to LUBAC by means of co-immunoprecipitation for SHARPIN in whole-cell lysates of SV40-immortalized dermal fibroblasts from an HC (WT/WT) and P3 (R57C/R57C) after rescue with WT OTULIN or EV. Data are representative of one experiment from three independent replicates. **d**,**e**, M1-Ub-selective TUBE analysis of LUBAC components (**d**) and M1-Ub (**e**) in whole-cell lysates of SV40-immortalized dermal fibroblasts from an HC (WT/WT) and P3 (R57C/R57C). Data are based on a single experiment. **f**,**g**, Assessment of M1-Ub (**f**) and LUBAC components (**g**) by means of co-immunoprecipitation for SHARPIN in whole-cell lysates of SV40-immortalized dermal fibroblasts from an HC (WT/WT) and P3 (R57C/R57C). Data are representative of one experiment from three independent replicates. **h**, Analysis of HOIL and HOIP following treatment with WT OTULIN of SHARPIN-co-immunoprecipitates of SV40-immortalized dermal fibroblast whole-cell lysates from P3. Data are representative of one experiment from three independent replicates.

**Extended Data Fig. 7:**
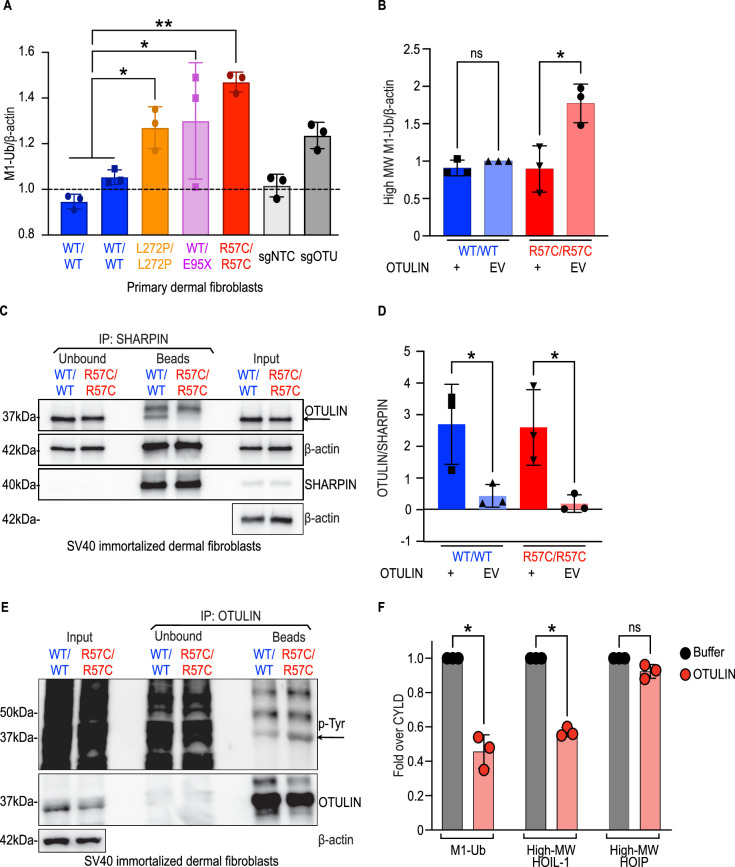
OTULIN-R57C patient dermal cells show accumulation of linear ubiquitin and aberrant LUBAC autoubiquitination. **A**) M1-Ub levels, semi-quantified over β-actin levels and subsequently normalized to those in whole cell lysates of OTULIN-KO dermal fibroblasts. Bars indicate standard deviations, n = 3 replicate experiments, *p < 0.05, **p < 0.005 one-way Anova with Dunnett’s multiple comparison test comparing indicated patients with healthy controls. Data are shown as mean ± SD. **B**) High-molecular weight (MW) M1-Ub over β-actin levels in whole cell lysates of SV40-immortalized dermal fibroblasts from a healthy control (WT/WT) and P3 (R57C/R57C) after rescue with WT OTULIN or empty virus (EV), normalized against cells from a healthy control transduced with an empty virus relative to cells rescued with an empty virus. Data from three independent replicates, mean and SD per group. *p < 0.05, statistical analysis by one-way ANOVA with Sidak multiple comparison correction. **C**) Co-immunoprecipitation of endogenous SHARPIN and OTULIN in whole cell lysates of SV40-immortalized dermal fibroblasts from one healthy control (WT/WT) and P3 (R57C/R57C). A representative image is shown of a biological duplicate experiment. **D**) Levels of OTULIN relative to SHARPIN as assessed by means of co-immunoprecipitation for SHARPIN in whole cell lysates of SV40-immortalized dermal fibroblasts from a healthy control (WT/WT) and P3 (R57C/R57C) after rescue with WT OTULIN or empty virus (EV). Data from three independent replicates, mean and SD per group. *p < 0.05, statistical analysis by One-way ANOVA with Sidak multiple comparison correction. **E**) Endogenous OTULIN immunoprecipitated from SV40-immortalized dermal fibroblasts of a healthy control (WT/WT) and P3 (R57C/R57C) and subjected to immunoblot against phosphorylated tyrosines. Arrow indicates signal corresponding to the apparent molecular weight of OTULIN. Data are based on a single experiment, and immunoprecipitates were split over two immunoblots for subsequent probing of phosphorylated tyrosines (p-Tyr) or OTULIN, respectively. **F**) M1-Ub, high-molecular weight (MW) HOIL1, and HOIP over CYLD levels following treatment with WT OTULIN of SHARPIN-co-immunoprecipitates of SV40-immortalized dermal fibroblast whole cell lysates from P3. Data from three independent replicates, mean and SD per group. *p < 0.05, one-way ANOVA with Sidak multiple comparison correction. Source data

Given the differences in LUBAC expression, TNFR-SC modification with M1-Ub, and RIPK1-and NF-κB-dependent signaling that distinguish ORAS and OTULIN-R57C skin cells, we hypothesized that intact LUBAC expression but deficient OTULIN–LUBAC binding in OTULIN-R57C cells may perturb M1-Ub homeostasis. We tested primary dermal fibroblasts for the accumulation of M1-Ub and compared cells from P3 with those from a patient with ORAS, an OTULIN haploinsufficient patient and OTULIN-KO cells derived from a healthy control^50^. As expected, overall levels of M1-Ub (in particular in the high-MW range) were increased in cells from the patient with ORAS in OTULIN-KO cells (Fig. 6a and Extended Data Fig. 7A). M1-Ub also accumulated in cells from P3 (Fig. 6a and Extended Data Fig. 7A), a phenotype that was rescued by genetic complementation with WT OTULIN (Fig. 6b and Extended Data Fig. 7B). Thus, OTULIN-R57C fails to regulate M1-Ub homeostasis. The essentiality of a direct interaction between OTULIN and LUBAC for maintaining M1-Ub homeostasis is controversial. Several reports argue that the PIM-PUB interaction, which is modulated by the phosphorylation of OTULIN at residue Y56, is critical for OTULIN to function as a DUB^37,39,42,44,90^. In contrast, other reports suggest that the phosphorylation event on OTULIN at residue Y56 has no impact on OTULIN’s catalytic activity but instead modulates RIPK1-dependent cell death^44^. Consistent with our ectopic expression data (Fig. 2d,e), OTULIN did not co-immunoprecipitate with LUBAC in dermal fibroblasts from P3, while it did in the cells from a healthy control (Fig. 6c and Extended Data Fig. 7C). This phenotype was rescued by genetic complementation of cells from P3 with WT OTULIN (Fig. 6c and Extended Data Fig. 7D). As in ectopic expression experiments (Fig. 2f), immunoprecipitation of OTULIN in dermal fibroblasts from P3 yielded an increased signal for phosphorylated tyrosines at an apparent MW corresponding with OTULIN, when compared to a healthy control (Extended Data Fig. 7E). Taken together, these data indicate that the deficient binding of OTULIN-R57C to LUBAC causes heightened overall levels of M1-Ub in the patient’s cells.

Autoubiquitination is one mechanism for regulation of LUBAC function. Autoubiquitination of LUBAC subunits, including HOIL1 and HOIP, underlies suppression of LUBAC-dependent protection from TNF-driven cell death^53,54,91^. Given the susceptibility of the patient’s cells to TNF-induced cell death, we tested levels of M1-Ub on LUBAC in dermal fibroblasts from P3 and a healthy control. First, we used an M1-Ub-selective tandem Ub-binding entity (TUBE) to probe for HOIP, HOIL1 and SHARPIN (Fig. 6d). In cells from P3, HOIL1, and to a lesser extent HOIP, appeared as high-MW species, suggesting decoration with M1-Ub (Fig. 6d). Given that analysis of LUBAC in the TUBE pulldown was biased by the overall abundance of M1-Ub in cells from P3 (Fig. 6e), we also co-immunoprecipitated LUBAC to probe for M1-Ub. The abundance of M1-Ub was greater in the LUBAC co-immunoprecipitate of cells from P3, when compared to cells from a healthy control (Fig. 6f). Again, a fraction of HOIL1 appeared as high-MW species (Fig. 6g). The high-MW HOIL1-species in cells from P3 were abolished by treatment with recombinant OTULIN (Fig. 6h and Extended Data Fig. 7F), indicating that HOIL1 is aberrantly decorated with M1-Ub in the patient cells. The autoubiquitination of LUBAC by means of HOIL1 may impair LUBAC function and underlie susceptibility of the patient’s skin cells to TNF-induced cell death. Collectively, these data identify OTULIN-R57C as a deficient regulator of M1-Ub homeostasis in the patient’s dermal fibroblasts, despite intact OTULIN-expression and DUB activity.

## Discussion

Here, we identify a monogenic etiology of severe, isolated PG of childhood, caused by homozygosity for the R57C allele in the PIM of OTULIN. In humans, the clinical consequences of OTULIN-R57C homozygosity are distinct from the phenotypes reported in previous studies of OTULIN-related IEI, all of which arise from OTU-domain mutations^44^. Clinical manifestations of OTULIN-R57C homozygosity are apparently restricted to the skin and are not accompanied by clinically overt systemic inflammation or heightened susceptibility to infections. Accordingly, the molecular and cellular mechanisms of disease we identified in OTULIN-R57C patients with PG differ from those in ORAS^45–49^, dominant-negative OTULIN deficiency^52,53^ and OTULIN haploinsufficiency^50^. We propose to refer to this disease as OTULIN-related PG, or ORP.

By comparing the whole-blood transcriptional profiles of patients with ORAS and ORP, we identified both overlapping and distinct patterns of gene expression. While patients with ORAS showed broadly aberrant transcriptional profiles, patients with ORP showed a more limited spectrum of genes that, nonetheless, included elevated expression of *IL1B* and *TNF*. The heightened production of these cytokines was confirmed in the plasma of patients with ORP. The increased IL-1β production was localized to patients’ monocytes and may reflect a failure of OTULIN-R57C to suppress inflammasome activation. ASC, a substrate for LUBAC^76^, is an adaptor protein for NLRP3- and pyrin-containing inflammasomes. Some mutant forms of *PSTPIP1* or *MEFV* can drive pyrin inflammasome hyperactivation and clinical PG^24–29^. Given that patients with ORAS also showed elevated *IL1B* in whole blood and that OTULIN-L272P also failed to suppress NLRP3 and pyrin inflammasome activity, we speculate that excessive IL-1β production via inflammasomes may be present in ORAS, but its clinical impact not overtly apparent due to the overwhelming NF-κB-driven systemic inflammation that typifies that condition. A favorable response to IL-1β-inhibition was reported in one patient with ORAS with prominent skin lesions^46^. Even so, our data do not demonstrate that excess inflammasome activation alone causes PG pathogenesis and instead suggests a multifactorial disease etiology with a critical role for TNF-dependent cell death. Elevated TNF, combined with OTULIN mutation-dependent impacts on the control of TNF-driven dermal cell death may also underlie the inflammatory and ulcerative PG lesions in patients with ORP. Genotype-dependent susceptibility to TNF-induced cell death in dermal cells from patients with ORAS and ORP is likely mediated by RIPK1, involving apoptosis and necroptosis in ORAS but resulting in a skewing toward apoptosis in ORP. Collectively, we propose a multifactorial mechanism of ORP caused by OTULIN-R57C dysfunction, including an interplay between myeloid cell inflammatory cytokine release and TNF-driven death of skin-resident cells. Additional studies are needed to develop a complete understanding of the molecular and cellular drivers of PG, including the identification and in-depth functional study of any additional human genetic etiologies of this disease.

Although normally expressed and catalytically intact, OTULIN-R57C fails to bind HOIP. In contrast to ORAS, and in agreement with the absence of systemic inflammatory disease in our patients, we could detect no marked impact of OTULIN-R57C homozygosity on the peripheral blood expression of several ISG and NF-κB-driven genes that are markedly dysregulated in ORAS. Similarly, we could not detect defects of inflammatory NF-κB signaling in ORP patients’ hematopoietic and nonhematopoietic cells following stimulation with TNF. These observations suggest that, while ORAS-causal mutations impair or ablate multiple OTULIN-dependent functions including DUB activity, OTULIN-R57C selectively perturbs pathways dependent on OTULIN-HOIP binding. We propose that the interaction between OTULIN and LUBAC may be required in certain cellular environments and conditions but not others, and that the R57C mutation uncouples OTULIN’s ability to bind HOIP from its DUB catalytic activity. Despite its catalytic integrity, OTULIN-R57C permits accumulation of M1-Ub in dermal cells indicating it fails to regulate M1-Ub homeostasis. While OTULIN is classically defined as a negative regulator of LUBAC, emerging evidence suggests this view is incomplete. In human fibroblasts, the principal role of OTULIN is not to counterbalance LUBAC but instead to sustain cell survival^54^. These findings highlight a broader principle that the physiological role of OTULIN is shaped by the cellular environment, the relative expression levels of LUBAC and OTULIN, and the signaling landscape of the cell. The combined autoinflammatory and immunodeficiency phenotypes in HOIL1 and HOIP deficiencies demonstrate the complex role of LUBAC in governing a cell type-specific balance between inflammation and immunity^51,92^. Our data in ORP further underscore this complexity.

In ORP, aberrant autoubiquitination of LUBAC and subsequent regulation of LUBAC function may underly susceptibility of the patient’s skin cells to TNF-induced cell death^53,54,91^. The mechanisms that govern LUBAC activity are incompletely understood. HOIP mutants containing the C-terminal RING-IBR-RING and linear ubiquitin chain determining domain (RBR-LDD) fragment but not its N terminus are constitutively active in the absence of SHARPIN and HOIP, whereas full-length HOIP is not^93,94^. Biochemical studies, thus, point toward an autoinhibitory function of the HOIP N terminus, but the molecular details of this autoinhibition remain unknown. The HOIP N terminus includes the PUB domain, to which OTULIN binds via its PIM. By binding to HOIP, OTULIN restricts LUBAC-mediated accumulation of M1-Ub chains^37^. Depletion of OTULIN results in the accumulation of M1-Ub on HOIP^36,38,50^, but ectopic expression of catalytically active OTULIN-Y56A, impaired for its binding to HOIP, does not^38^. Abundant autoubiquitination of HOIL1 rather than HOIP indicates that pathogenic LUBAC activity in cells from patients with ORP is caused by a defect of another regulatory mechanism. Autoubiquitination of LUBAC, and LUBAC functions, are also controlled by the ligase activity of HOIL1 (ref. ^91^). Like in cells from patients with ORP, dominant-negative OTULIN deficiency acts at the interface of OTULIN and LUBAC^52^, and results in accumulation of M1-Ub on HOIL1 but less so on HOIP^53^. The mechanism of M1-Ub accumulation in ORP patients’ skin cells likely includes the defective removal of inhibitory M1-Ub from LUBAC, possibly via loss of HOIL1-ligase activity^91^.

**Fig. 7:**
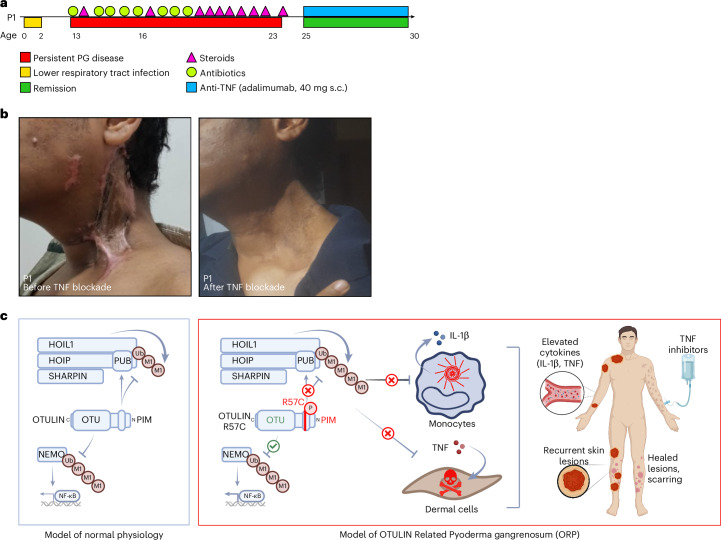
Patient 1 achieved disease remission with TNF blockade. **a**, P1 clinical timeline. Age is represented as years. Boxes below the line represent periods of lower respiratory tract infection (LRTI, yellow) or PG flares (red). Symbols above timeline represent therapies P1 has received (see legend). At the age of 25, P1 started receiving monthly subcutaneous (s.c.) injections of 40 mg adalimumab (anti-TNF) and symptoms of PG resolved. Use of adalimumab and PG-free clinical status have been maintained for nearly 5 years. **b**, PG lesion on P1’s neck, showing complete resolution after TNF blockade. **c**, Pathophysiological model of ORP. OTULIN-R57C is catalytically active, but unable to bind and regulate LUBAC. In myeloid cells, OTULIN-R57C fails to suppress inflammasome activation. Patient dermal fibroblasts accumulate linear ubiquitin, show aberrant LUBAC autoubiquitination and are sensitized to TNF-dependent cell death. Patients with ORP clinically respond to TNF blockade. Figure created in BioRender; Spaan, A. https://BioRender.com/hqdpmjh (2026).

This study underscores the scientific and translational opportunities presented through direct interrogation of patients with rare diseases. As identifying the molecular cause of a disease is essential for selecting targeted therapies, our findings have direct therapeutic implications. P1 was treated empirically for several years with antibiotics and steroids with limited success (Fig. 7a). At 25 years of age, he developed a large ulcerative lesion on his neck (Fig. 7b) and began treatment with monthly subcutaneous injections of the anti-TNF monoclonal adalimumab. He has since achieved complete remission with continued anti-TNF therapy. Anti-TNF therapies have been used to treat PG in patients without a known genetic basis, with variable success^11–13,17,18,95,96^. Similarly, IL-1β blockade has been attempted in PG with variable outcomes^19,20,81,84^. Anti-TNF therapies have also been efficacious in some patients with ORAS and dominant-negative OTULIN deficiency^45–47,49,53^. Larger studies are needed to determine the broader clinical utility of blocking IL-1β or TNF in PG^13,20,97^. Taken together, our findings establish ORP as a genetic cause of severe, isolated PG of childhood and reveal the immune mechanisms contributing to disease pathogenesis. Based on these findings, we propose a model of ORP pathophysiology (Fig. 7c). By dissecting the molecular foundation of this extremely rare and severe disease, our work highlights the broader relevance of OTULIN in immune regulation and provides a foundation for developing targeted interventions for patients with PG.

The identification of OTULIN-R57C homozygosity as a genetic basis for pediatric PG would not have been anticipated based on current knowledge from animal models. Murine models have defined skin cell-specific roles for OTULIN in the control of TNF-driven cell death^87,88^ and myeloid cell NF-κB activity^45^; however, there are also important species-specific aspects of LUBAC and OTULIN biology that may preclude direct comparisons of molecular and cellular mechanisms of diseases (including PG) across species. For example, patients with HOIL1 deficiency have elevated levels of circulating TNF^92^, whereas Hoil1-KO mice do not^98^. Sharpin-deficient mice develop keratinocyte-driven dermatitis^56,99–102^, yet SHARPIN-deficient patients are free of dermatological disease^103^. Similarly, the mechanisms of skin cell-intrinsic immunity underlying disease in human OTULIN haploinsufficiency are not recapitulated in mice^50^. Mice with keratinocyte-specific Otulin deficiency or with the Otulin-L272P allele specifically expressed in keratinocytes develop dermatitis^87,88^ and express elevated ISGs^87^, similar to patients with ORAS^46,47,53,72^ and unlike patients with ORP. Inactivation of Ripk1 can rescue dermatitis in these mice^87,88^. We showed that RIPK1 inhibition rescued TNF-CHX-induced susceptibility to cell death in dermal fibroblasts from patients with ORAS, but not ORP. Thus, keratinocyte-specific Otulin-KO mice phenocopy the skin manifestations of ORAS, but not of ORP caused by OTULIN-R57C homozygosity. A recent study showed that mice homozygous for Otulin-Y56A, which impairs Otulin–LUBAC binding, are hypersensitive to TNF-induced toxicity^104^. Mirroring patients with ORP, homozygosity for Otulin-Y56A in mice is characterized by accumulation of M1-Ub at the TNFR-SC, heightened autoubiquitination of Hoil1 and susceptibility to Ripk1 inhibition-resistant cell death^104^. In contrast to patients with ORP, Otulin^Y56A/Y56A^ mice do not show spontaneous inflammatory phenotypes. Instead, TNF administration to Otulin^Y56A/Y56A^ mice triggers pathology in liver and intestine rather than the skin^104^. These findings highlight striking interspecies differences in the cell-type-specific inflammatory consequences of LUBAC and OTULIN activity and support the scientific utility of patient-based studies.

## Methods

### Whole-exome sequencing

Blood samples from all four members of Pedigree 1 were subjected to genomic DNA extraction using GeneJET Genomic DNA Purification kit (Thermo Scientific K0721) followed by WES. Exome capture (Twist Biosciences Exon) was performed followed by paired-end sequencing on a NovaSeq (Illumina) generating >2 × 10^7^ 150-base pair reads per sample. Variants that were not in coding or splicing regions, and those present at >1% MAF using exome data from Genome Aggregation Database (gnomAD)^105^ were filtered out. Combined Annotation-Dependent Depletion^33^ and PolyPhen-2 (ref. ^34^) scores on the remaining three candidate genes were calculated. Genomic DNA from the two members of Pedigree 2 was extracted from whole blood. WES of P3 was performed as described elsewhere^50^.

### Sanger sequencing

Genomic DNA from all four members of Pedigree 1 and two members of Pedigree 2 were used for Sanger sequencing confirmation of the *OTULIN* mutation. Polymerase chain reaction (PCR) was performed for exon 2 of the *OTULIN* gene on chromosome 5 using primers OTULIN-gDNA-F1 and OTULIN-gDNA-R1. The resulting PCR products were purified using QIAquick PCR Purification kit (QIAGEN 28104). Sanger sequencing reactions were carried out using the BigDye Direct Cycle Sequencing kit (Thermo Fisher 4458687). Samples were filtered through Sephadex G-50 Superfine (GE Healthcare 17-0041-01) into 96-well, semi-skirted plates (Thermo Fisher AB1400L). ABI3130xL Genetic Analyzer (Thermo Fisher 313001R) was used to sequence the resulting products. Refer to Supplementary Data Table 1 for primer sequences.

### Preparation of PBMCs and plasma samples

Whole blood was centrifuged for 15 min at 2,000*g* at room temperature to separate plasma. The remaining samples used to prepare PBMCs using SepMate tubes (STEMCELL Technologies 85450) and Ficoll-Paque (Cytiva 17144002).

### B-lymphoblastoid cell lines

Epstein–Barr Virus (EBV)-transformed B-lymphoblastoid cell lines (B-LCL) were generated as previously reported^106^. In brief, 1 × 10^6^ PBMCs were collected and resuspended in 1 ml complete RPMI medium. Then, 500 μl EBV-containing supernatant and 1 μg ml^−1^ of cyclosporine A were added to PBMCs and incubated at 37 °C with 5% CO_2_.

### Preparation of dermal fibroblasts

Primary dermal fibroblasts were obtained from skin biopsy specimens as described elsewhere^50^. The characterization of OTULIN-knockout primary dermal fibroblasts was described elsewhere^50^. Immortalized dermal fibroblasts were generated by transformation with SV40 as previously described^107^ and were subsequently cultured under identical conditions as primary dermal fibroblasts.

### DNA cloning and site-directed mutagenesis

For experiments shown in Figs. 2a–d and 4a–c and Extended Data Fig. 1C,D, site-directed mutagenesis was used to create a stop codon directly following the Myc tag in Myc-DDK-tagged OTULIN (OriGene RC224840), or directly following the Flag tag in Flag-tagged SHARPIN (Addgene 50014) and Flag-tagged HOIL (Addgene 50016) using primers in Supplementary Data Table 1 and PfuUltra II (Agilent 600670). For experiments shown in Fig. 2f and Extended Data Fig. 1E, the human canonical *OTULIN* cDNA open-reading frame (ORF) clone was amplified from pCMV6-Entry FAM105B (OriGene RC224840) and inserted with a stop codon before the Myc-DDK tag into the pCMV6-AN-Myc-DDK–tagged vector (OriGene PS10001) as described elsewhere (pCMV6-AN-OTULIN)^50^. Site-directed mutagenesis was then used to remove the stop codon using primers in Supplementary Data Table 1 and Phusion Hot Start II High-Fidelity PCR Master Mix (Thermo Fisher F566L), resulting in an intact C-terminal Flag-tagged construct (pCMV6-AN-OTULIN-WT-Flag). The resulting plasmids were sequenced and expressed in HEK293T cells to ensure the presence or absence, respectively, of the Flag tag. Plasmids were then used for further site-directed mutagenesis to create OTULIN variants using primers in Supplementary Data Table 1. The coding sequence of human TNF (amino acid residues V77-L233) with a N-terminal Flag tag and an 8 amino acid linker was cloned into the pcDNA3.4 (Thermo Fisher Scientific A14697) vector using the Gibson Assembly Master Mix (New England Biolabs E2611L) using primers provided in Supplementary Data Table 1.

### Expression of Flag–TNF

Recombinant human Flag–TNF was brought to expression by transfecting in an Expi293F expression cells (Thermo Fisher Scientific A14527) using polyethyleneimine HCl MAX (PEI) (PolySciences 24765). TNF was collected from the supernatant on day 5 by clarifying the cell suspension. The supernatant was sterile filtered and stored at 4 °C.

### Cell culture and transfections

HEK293T (ATCC CRL-3216) cells were cultured in Dulbecco’s Modified Eagle Medium (DMEM) supplemented with 10% heat-inactivated FBS and 100 U ml^−1^ each of penicillin, streptomycin and amphotericin B (Thermo Fisher 15240062). Expi293F cells were cultured in Expi293 expression medium (Thermo Fisher A1435101). N/TERT-1 keratinocytes^108,109^ were cultured in complete K-SFM (Gibco 17005042). Primary dermal fibroblasts and immortalized dermal fibroblasts were cultured in DMEM supplemented with GlutaMAX and pyruvate (Gibco 31966021) and 10% FBS. Plasmid transfections were performed using Lipofectamine 2000 (Invitrogen 11668030) or Lipofectamine LTX (Invitrogen 15330120) and Opti-MEM (Thermo Fisher 31985062). Cells were recovered 18 h after transfection unless stated otherwise. WT OTULIN, OTULIN-R57C and mCherry (designated as EV) were cloned into a RP139 lentiviral transduction vector using the primers mentioned in Supplementary Data Table 1 and co-transfected into HEK293T cells with plasmids containing the packaging mixes of pMD2G-VSVg, pRSV-REV and pMDL/RRE using Mirius LT1 transfection reagent (MIR 2306) as described elsewhere^110^. Three days later, the supernatants containing the lentivirus preparation were recovered and 100 μl supernatant was added to semi-confluent SV40-immortalized dermal fibroblasts. Transduced cells were isolated by selection with puromycin and expanded. Refer to Supplementary Data Table 2 for further details on plasmids.

### Stimulation of primary PBMCs and ELISA

Primary PBMCs were seeded at a density of 2 × 10^5^ cells per well in a 96-well flat-bottomed plate in RPMI containing 10% FBS. The cells were stimulated with 1 μg ml^−1^ LPS (Enzo ALX-581-013-L001) for 4 h and 5 μM nigericin (Millipore Sigma 5N7143) for 1 h. Supernatants were recovered and stored at −70 °C until use. The levels of IL-1β were assessed with the BD OptEIA Human IL-1β ELISA Set II kit (BD 557953) in accordance with the manufacturer’s protocol.

### Stimulation of B-LCL

A total of 5 × 10^5^ cells per well were plated in 500 μl medium in a 48-well plate and treated with 50 ng ml^−1^ TNF and collected at 15-min increments over a total of 60 min. Cells were collected and lysed in 25 mM Tris, 0.15 M NaCl, 1 mM EDTA, 1% NP40 and 5% glycerol; pH 7.4 and protease inhibitor cocktail (Thermo Fisher A32953) on ice for 30 min and cleared by centrifugation at 16,000*g* for 15 min at 4 °C before being used for immunoblotting.

### Stimulation of primary and immortalized dermal fibroblasts

Primary dermal fibroblasts were seeded to confluency in six-well culture plates. Then, 4 h after seeding, the medium was replaced with DMEM containing 1% FBS. 24 h after seeding, cells were stimulated with 50 ng ml^−1^ TNF (R&D Biosystems 210-TA-005), in the absence or presence of 31.5 μg ml^−1^ cycloheximide (MERCK 239765), and in the absence or presence of 30 µM Z-VAD-FMK (ApexBio A1902). The cells were recovered at the relevant time points and lysed. For analyses of the TNF receptor signaling complex, SV40-immortalized dermal fibroblasts were stimulated for 10 min with Expi293 expression supernatant containing Flag–TNF, washed and lysed.

### Stimulation of HEK293T cells

HEK293T cells were stimulated with 100 ng ml^−1^ TNF (R&D Biosystems 210-TA-005) and 20 µM MG-132 (C2211 Sigma Aldrich) for 4 h. The medium was refreshed with DMEM containing 10 µM sodium orthovanadate for 2 h before recovery and immunoprecipitation.

### Whole cell lysates

The following whole-cell lysate (WCL) buffers were used. WCL buffer A: 25 mM Tris, 150 mM NaCl, 1 mM EDTA, 1% NP40 and 5% glycerol; pH 7.4; WCL buffer B: 50 mM Tris, NaCl 150 mM, 1% NP40 and 0.1% sodium dodecyl sulfate at pH 7.5; and WCL buffer C: 20 mM Tris, NaCl 150 mM, EDTA 2 mM, Triton-X-100 1% and glycerol 10% at pH 7.5. To all WCL buffers, protease inhibitor cocktail (Thermo Fisher A32953) or Complete Protease Inhibitor (Roche 11836170001) was added. PhosSTOP (Roche 04906845001), sodium fluoride 15 mM (Sigma Aldrich 201154) and sodium orthovanadate 10 mM (MedChemExpress MD2-100-208) were added to WCL buffers intended for investigation of phosphorylation status. *N*-ethylmaleimide (NEM) 7.5 mM (Sigma Aldrich 04529) and PR-619 5 µM (Tebu-Bio SI9619) were added to WCL buffers intended for investigation of M1-Ub status. Resting HEK293T cells were lysed in WCL buffer A, and stimulated HEK293T cells were lysed in WCL buffer B. Primary dermal fibroblasts and immortalized dermal fibroblasts intended for WCL analysis were lysed in WCL buffer B. Primary dermal fibroblasts and immortalized dermal fibroblasts intended for analyses of co-immunoprecipitations were lysed in WCL buffer C. Cells were lysed on ice for 30–45 min, and cleared by centrifugation at 16,000*g* for 15 min at 4 °C.

### Immunoblotting

HEK293T whole cell lysates were mixed with 1× SDS–PAGE loading buffer and boiled at 95 °C for 5 min, then resolved and analyzed by SDS–PAGE. Primary dermal fibroblast or immortalized dermal fibroblasts WCLs and immunoprecipitates were boiled at 95 °C in 1× Laemmli buffer (Bio-Rad 1610741) supplemented with 100 mM DTT before loading into PROTEAN TGX gels (Bio-Rad). The proteins were transferred onto a Trans-Blot Turbo Mini 0.2 µm PVDF Transfer Pack membranes (Bio-Rad 1704158) and blocked with phosphate-buffered saline (PBS) supplemented with 0.1% Tween20 (PBS-T) and 5% bovine serum albumin (BSA) (Serva 11930.03) or EveryBlot blocking buffer (Bio-Rad 12010020). Tris-buffered saline (TBS) was used when the phosphorylation status was investigated. Primary antibodies used for immunoblotting were mouse anti-c-Myc (clone 4F6, Vanderbilt Antibody and Protein Resource VAPR4F6), mouse anti-HA tag (Invitrogen 26183), mouse anti-Flag antibody (clone M2, Millipore Sigma F1804), mouse anti-V5 tag (Thermo Fisher R960-25), mouse anti-linear ubiquitin (clone LUB9, Millipore Sigma MABS451), rabbit anti-OTULIN (Cell Signaling Technology 14127 and Abcam 151117), rabbit anti-HOIP rabbit (Abcam Ab46322), rabbit anti-SHARPIN (Cell Signaling Technology 12541), mouse anti-HOIL1 (Santa Cruz sc-393754), rabbit anti-phospho-tyrosine (Cell Signaling Technology 8954), rabbit anti-TNFR1 (Thermo Fisher 21574-1-AP), rabbit anti-TNFR2 (Thermo Fisher MA5-32618), mouse anti-TNFR1 (Santa Cruz 8436), rabbit anti-phospho-IκBα (Cell Signaling 2859), rabbit anti-IκBα (Cell Signaling 9242), rabbit anti-phospho-p65 (Ser536) (Cell Signaling 3033), rabbit anti-p65 (Cell Signaling 8242), rabbit anti-cleaved caspase-3 (Cell Signaling 9662 and 9664), rabbit anti-phospho-RIP (Ser166) (Cell Signaling 65746), rabbit anti-RIP (Cell Signaling 3493), rabbit anti-phospho-RIP3 (Ser227) (Cell Signaling 93654), rabbit anti-RIP3 (Cell Signaling 13526), rabbit anti-phospho-MLKL (Ser358) (Cell Signaling 91689), rabbit anti-MLKL (Cell Signaling 14993), mouse anti-α-tubulin (Thermo Fisher Scientific A11126), mouse anti-β-actin (Abgent AM1829b) and mouse anti-GAPDH (clone 6C5, Invitrogen AM4300). Antibodies were diluted in TBS-T containing 5% nonfat dry milk powder (for HEK293T WCLs) and PBS-T or TBS-T (for primary and SV40-immortalized dermal fibroblast WCLs) supplemented with 1% BSA. Secondary HRP-conjugated antibodies used were goat anti-rabbit IgG (Southern Biotech 4030-05) and goat anti-mouse IgG1 (Southern Biotech 1070-05). Blots were revealed using Pierce ECL Plus Western Blotting Substrate (Thermo Fisher 32106), SuperSignal West Pico PLUS Chemiluminescent Substrate (Thermo Fisher 34578×4) or SuperSignal West Femto Maximum Sensitivity Substrate (Thermo Fisher 34095). Images were captured on an Amersham Imager 680 Blot and Gel Imager (Cytiva 29270769), ImageQuant LAS 4000 luminescent image analyzer (GE Healthcare) or ChemiDoc MP Imaging System (Bio-Rad). Intensity analyses were performed with ImageStudioLite (LI-COR).

### Linear deubiquitination assay

HEK293T cells were co-transfected with plasmids encoding WT or mutant forms of OTULIN (refer to Supplementary Data Table 2), pCMV3Flag8SHARPIN, pCMV3Fag8HOIP, and pCMV3Flag8HOIL, pcDNA3-HA-NEMO and/or EV as described above.

### Co-immunoprecipitation

HEK293T cells were co-transfected with plasmids encoding WT or mutant forms of OTULIN, SHARPIN, HOIP, HOIL1, NEMO and/or EV (Supplementary Data Table 2). Lysate was incubated with Pierce Anti-c-Myc magnetic beads (Thermo Fisher 88842), Pierce Anti-HA Tag IP/Co-IP kit (Thermo Fisher 88838), or anti-Flag magnetic beads (Thermo Fisher M8823) for 30 min at room temperature on a rotor. 1× SDS–PAGE loading buffer in lysis buffer was used to elute bound proteins, which were boiled at 95 °C for 5 min, then resolved and analyzed by SDS–PAGE. For co-immunoprecipitations in primary and immortalized dermal fibroblast cell lysates, protein A beads (Thermo Fisher 10001D) were conjugated with the capture antibodies in PBS-T or TBS-T by incubating them at room temperature for 1 h. The capture antibodies used were rabbit anti-SHARPIN (Proteintech 14626-1-AP) and rabbit anti-OTULIN (Cell Signaling Technology 74125). The antibody-conjugated beads or anti-Flag magnetic beads (Thermo Fisher M8823) were incubated with WCLs at 4 °C overnight on a rotor. The unbound fractions were stored, while the bound proteins were eluted by boiling the beads at 95 °C in sample buffer containing 1× Laemmli buffer supplemented with 100 mM dithiothreitol (DTT) before proceeding to immunoblotting.

### Linear ubiquitin-selective tandem Ub-binding entity DUB treatment of LUBAC assays

For M1-Ub pulldown, SV40-immortalized fibroblasts were lysed for 30 min on ice in WCL buffer C in the presence of biotin-conjugated M1-Ub-selective TUBE according to the manufacturer’s recommendations (LifeSensors). Supernatants were cleared by centrifugation, adjusted for total protein concentration and incubated overnight at 4 °C with Streptactin beads (Sigma Aldrich). For DUB-treatment of LUBAC, SHARPIN-co-immunoprecipitates of SV40-immortalized fibroblasts were equilibrated in UbiCREST DUB reaction buffer and incubated with recombinant UbiCREST OTULIN for 30 min at 37 °C according to the manufacturer’s recommendations (R&D Systems). For both the TUBE assay and DUB-treatment of LUBAC assay and after washing the beads, bound proteins were eluted by boiling the beads at 95 °C in sample buffer containing 1× Laemmli buffer supplemented with 100 mM DTT before proceeding to immunoblotting.

### NF-κB luciferase reporter assay

Plasmids 4xNFkB-Luc (Addgene 111216) and pRL-SV40P (Addgene 27163) were used to co-transfect in HEK293T cells along with expression plasmids encoding WT or mutant forms of OTULIN, SHARPIN, HOIP, HOIL1, NEMO and/or EV (Supplementary Data Table 2). Luciferase activity was measured at 30 h post-transfection by the DualGlo Luciferase Assay System (Promega E2920). Firefly luciferase activity was normalized to untransfected luciferase activity. Results are expressed as fold change.

### RNA extraction and mRNA expression

Blood samples were collected in PAXgene Blood RNA tubes (BD Biosciences 762165). RNA was purified using GeneJET RNA Purification kit (Thermo Fisher K0732). Targeted gene arrays were performed using an nCounter Immunology Panel with probes for 594 human genes (NanoString XT-CSO-HIM2-12, NanoString Technologies). Raw RCC files were imported into nSolver Analysis Software v.4.0. Background thresholding value was estimated from negative control probes and set to 20. Data were normalized using the geometric mean of 11 housekeeping genes (*EEF1G*, *G6PD*, *GAPDH*, *HPRT1*, *OAZ1*, *POLR2A*, *PPIA*, *RLP19*, *SDHA*, *TBP* and *TUBB*), as suggested by the manufacturer. Probes were excluded from further analysis if <75% of samples had values below the detection threshold. The counts for each probe were compared using two-tailed student’s *t*-tests, and data were plotted with GraphPad Prism 9 (GraphPad Software). Samples included in these analyses are from a first set of HCs (*n* = 9 run in the same batch as P1 and P2 samples, left-most columns of Fig. 3 and Extended Data Fig. 3A,B), two samples each from P1 and P2, drawn at different times during nonflare periods, another set of HCs (*n* = 4 run in the same batch as samples from patients with ORAS) and samples from three patients with ORAS (with OTULIN mutations Y244C/Y244C, G174Dfs*2/G174Dfs*2 and L272P/L272P, respectively).

### Multiplex electrochemiluminescent immunoassay

Concentrations of cytokines in plasma were determined using the V-PLEX Assay Platform Viral Panel 3 Human kit (Meso Scale Discovery K15347D-1) and the V-PLEX Assay Platform Cytokine Panel 1 Human kit (Meso Scale Discovery K15050D-1). Calibrator blends that come with each kit were used as standard controls for the assay. Statistical analysis was performed using unpaired *t*-tests (GraphPad Prism v.9).

### Cytometry by time of flight

Approximately 5 × 10^6^ PBMCs were stained with Cell-ID cisplatin-195Pt (Fluidigm 201194). PBMCs were then FcR blocked with Human TruStain FcX (BioLegend 422302), stained with the antibody mix as shown in Supplementary Data Table 3, fixed with 1.6% paraformaldehyde, and stained with Cell-ID intercalator-Ir (Fluidigm 201192A). Stained PBMCs were analyzed in a Helios CyTOF 3.0 (Fluidigm) following the manufacturer’s instructions. The data was exported as a Flow Cytometry Standard file (FCS) and normalized using EQ-Four calibration beads standards (Fluidigm 201078) following the manufacturer’s protocol. For multidimensional analysis, the data were pre-gated to remove dead cells, debris and selection of leukocytes using FlowJo v.10.7.2 (Becton, Dickinson & Company). The pre-gated data were exported as FCS files and imported into RStudio (v.4.4.1). The CATALYST package^111^ was used to arcsine transform marker intensities with a cofactor of 5 and performed subsequent analysis. Unsupervised clustering was performed using FlowSOM^112^ to generate 20 unsupervised clusters, and data representation of these clusters was performed using the R package ggplot2. Subsequent manual clustering generated eight clusters based on the original 20 cluster FlowSOM heat map of marker intensities, and these manual clusters were represented through *t*-SNE plots, which were standardized at the lowest cell count across all samples (9,500 cells). Manual gating was carried out using FlowJo v.10.7.2 (Becton, Dickinson & Company). Exported cell subset frequency data were graphed and statistically analyzed using GraphPad Prism v.9.

### Single-cell RNA sequencing

#### Sample preparation

PBMCs were quickly thawed at 37 °C and resuspended in DMEM + 10% heat-inactivated FBS. A total of 1 million cells were resuspended in 200 µl of 1× PBS, 2% FBS and 1 mM CaCl_2_, then the suspension was flowed through a 40-μm Flowmi cell strainer (Millipore Sigma BAH136800040), and dead cells were removed with the EasySep Dead Cell Removal kit (STEMCELL Technologies 17899). The samples were loaded onto a 10x Genomics Chromium chip. Reverse transcription and library preparation were performed with Chromium Single Cell 3ʹ Reagent kits (v.3.1). Library quality was assessed with a Bioanalyzer DNA chip, and the libraries were sequenced on one lane (S4 flowcell) of an Illumina NovaSeq 6000 sequencer.

#### Quality control

Removal of ambient RNA was carried out using SoupX (v.1.6.2) and output HDF5 files were analyzed with the Seurat package (v.5.2.1) in R (v.4.4.1). The following genes and cells meeting the described criteria were removed from downstream analyses: genes found in <3 cells; cells expressing <10 genes; cells containing >10% mitochondrial genes; genes with total read counts <5% or >95% of the quantile distribution across each sample; or doublets predicted using the scDblFinder package (v.1.18.0).

#### Cell-type annotation

Cell cycle scores were calculated using CellCycleScoring and the difference between S and G2M scores were regressed out using SCTransform. Anchors between reference and query were precomputed using an annotated PBMC Cite-seq ref. ^113^, FindTransferAnchors using 50 dimensions, and a precomputed principal-component analysis transformation. Cell-type labels and protein data from the reference to the query were transferred using MapQuery. Low-confidence cells (prediction level 1 score <0.5 and mapping scores <0.5) were removed from further analysis. UMAP cell type density plots of both supervised and unsupervised analyses were created using the kde2d function from the MASS package (v.7.3-65).

#### Pseudobulk and gene set analysis

Samples were pseudobulked by genotype using the AggregateExpression function and DEGs between patients and HCs were identified with the FindMarkers function using DESeq2 with default parameters. 985 genes passed adjusted *P* value thresholds (*P* < 0.05). Two commonly used gene set analysis methods were used to quantitatively measure gene set activity within a single cell (AUCell (v.1.26.0) and the AddModuleScore function). To measure activity of the top 15 DEGs within R57C patients and HCs, both module scores and area under the curve scores were calculated. AUCell was run to account for low gene set size and level 1 cell type number, particularly among dendritic cells (DCs).

#### Characterizing OTULIN-R57C patient monocytes

DEGs between patient and HC monocytes were identified by pseudobulking using AggregateExpression. Next, DEA was performed using the DESeq2 test method inside the FindMarkers function with default parameters. DEGs were defined as those with a fold change of gene expression ≤ 0.5 or ≥ 1.5 and adjusted *P* < 0.05.

#### Characterizing monocyte subtypes

Gene set activity of two distinct subtypes within monocytes, classical and nonclassical, was calculated using AUCell across cell type-specific genes. In brief, *CD14*, *S100A8*, *S100A9*, *S100A12* and *CD99* were used to identify classical monocytes. *FCGR3A*, *CXCR3*, *CDKN1C*, *HES4*, *CD79B* and *RHOC* were used to identify nonclassical monocytes^75^.

### ASC speck visualization by microscopy

A total of 3 × 10^4^ cells of HEK293T cells stably expressing GFP-tagged ASC were plated onto coverslips in 500 μl DMEM + 10%FBS without antibiotics. Cells were allowed to attach overnight at 37 °C, 5% CO_2_. Cells were co-transfected with plasmids encoding WT or D305N NLRP3 and WT, or WT MEFV, and WT or mutant forms of OTULIN (refer to Supplementary Data Table 2). At 18 h post-transfection, cells were fixed in 300 μl 1.6% paraformaldehyde for 20 min, washed with PBS, then permeabilized with 300 μl 0.5% Triton-X for 10 min. Cells were washed 3× in PBS. Coverslips were loaded onto microscope slides using ~10 μl DAPI-containing Prolong Gold mounting medium (Thermo Fisher P36941). After drying for ≥1 h, ASC specks and nuclei were visualized using the Olympus BX51 fluorescence microscope under a ×40 objective with GFP and DAPI filters, respectively. Fluorescent images of ASC specks were quantified using ImageJ.

### CRISPR-Cas9 KO of *OTULIN*

We designed single guide RNA (sgRNA) oligonucleotides targeting exons 4 and 6 of *OTULIN*, and nontargeting gRNAs (Supplementary Data Table 4). A DNA duplex was annealed from the oligonucleotides and inserted into pLenti-CRISPR-V2 (Addgene 52961). We then transfected the resulting plasmid, the VSV-G envelope-encoding pMD2.G (Addgene 12259), and the lentiviral packaging plasmid psPAX2 (Addgene 12260), in HEK293T cells in the presence of Lipofectamine 2000 (Invitrogen 11668030). Virus was collected after 72 h and centrifuged 300*g* for 5 min twice to remove cellular content. Then, 1 × 10^5^ target cells were combined with 1 ml of viral supernatant, 8 μg ml^−1^ Polybrene (Millipore Sigma TR-1003) and 1% PEG (Thermo Fisher J61495-AK). Cells were centrifuged for 1 h at 1,000*g*. The supernatant was removed and cells resuspended in 2 ml fresh medium. Cells were left to rest 24 h at 37 °C before selection with puromycin.

### Kinetic microscopy cell death assay

WT, nontarget and OTULIN-KO N/TERT-1 cells were and plated at 10,000 cells in 200 μl medium in appropriate wells of a flat-bottom clear, black-sided 96-well plate (Corning CLS3603). Cells were allowed to adhere overnight at 37 °C, 5% CO_2_. Immediately before imaging, the medium was replaced with 200 μl K-SFM + PI (Millipore Sigma P4170) and treated with 100 ng ml^−1^ TNF (Preprotech 300-01A). For each well, at least three beacons were defined for imaging. All wells were imaged every 2 h for a total of 72 h at 37 °C, 5% CO_2_ using the ×20 objective with phase contrast + Texas Red filter settings with the Cytation 5 (Agilent Biotek). Data analyses were performed using the Gen5 software (Agilent Biotek). Mean RFI values for each well at each time point were calculated and plotted with GraphPad Prism 9. Phase contrast + Texas Red filtered images were aligned and exported as .tif files. For analysis of genetically complemented N/TERT-1 keratinocyte lines, experiments were performed as described above and Texas Red values at 72 h were normalized to Texas Red values within the same well at time 0 h to obtain normalized values for each experimental condition. Data obtained from independent experiments (*n* = 4) are shown.

### Genetic complementation of OTULIN-KO N/TERT-1 cells

To permit re-expression of WT and mutant forms of OTULIN protein in OTULIN-KO N/TERT-1, *OTULIN* ORFs containing the appropriate missense mutations (R57C and L272P) as well as single-nucleotide variants in the gRNA-binding site and PAM that ablated Cas9 cleavage but did not alter amino acid sequence (5′-GTGATAATTACTGTGCACTGAGG-3′ to 5′-GTGATAATTATTGCGCTTTAAGA-3′) were synthesized (GenScript) and cloned into pLX303 (Addgene 25897). Lentiviruses were used to transduce OTULIN-KO N/TERT-1 keratinocytes followed by selection using Blasticidin (5 μg ml^−1^).

### IL-6 expression assays

Primary dermal fibroblasts were seeded at a density of 2 × 10^5^ cells per well in a 48-well flat-bottomed plate in DMEM containing 10% FBS. At 4 h after seeding, the medium was replaced with DMEM containing 1% FBS. At 24 h after seeding the cells were stimulated with 20 ng ml^−1^ TNF (R&D Biosystems 210-TA-005). Then, 24 h after stimulation, the supernatants were recovered in a 96-well deep-dish plates stored at −80 °C until use. The levels of IL-6 were assessed with IL-6 Human ELISA kits (Invitrogen 88706688) in accordance with the manufacturer’s protocol and as described elsewhere^51,105^.

### Fibroblast viability assays

Primary dermal fibroblasts were seeded at a density of 2 × 10^4^ cells per well in a 96-well flat-bottomed plate in DMEM containing 10% FBS. 4 h after seeding, the medium was replaced with DMEM containing 1% FBS. Then, 24 h after seeding, the cells were stimulated with 20 ng ml^−1^ TNF (R&D Biosystems 210-TA-005) and 12.5 μg ml^−1^ cycloheximide, plus 30 μM Z-VAD-FMK (ApexBio A1902), 25 μM Necrostatin-1 (Merck n9037) or 30 μM GSK872 (Merck 5.30389) in respective combinations. Then, 24 h after stimulation, cell viability was measured using CellTiter-Glo Luminescent Cell Viability Assay (Promega G7571) in accordance with the manufacturer’s protocol. Viability was expressed relative to cells not incubated with TNF and cycloheximide.

### Statistics

Data are expressed as the mean of ≥3 biological replicates ± s.d. or are representative of ≥3 independent experiments, unless otherwise indicated. For most data, linear mixed models were used for log-transformed relative values to account for repeated measurements. Where indicated, two-tailed unpaired *t*-tests were performed to generate *P* values, and data were plotted with GraphPad Prism v.9 (GraphPad Software).

### Study approval

This study was approved by and performed in accordance with the institutional ethics committees at Vanderbilt University Medical Center (protocol 200412) and the Rockefeller University (protocols JCA-0698 and JCA-0695). Written informed consent was obtained for the patients, family members and HCs enrolled in this study.

### Reporting summary

Further information on research design is available in the Nature Portfolio Reporting Summary linked to this article.

## Online content

Any methods, additional references, Nature Portfolio reporting summaries, source data, extended data, supplementary information, acknowledgements, peer review information; details of author contributions and competing interests; and statements of data and code availability are available at 10.1038/s41590-026-02568-6.

## Supplementary information

Reporting Summary

Supplementary Table 1 Primers.

Supplementary Table 2 Plasmids.

Supplementary Table 3 CyTOF antibodies.

Supplementary Table 4 CPRISP Cas9 gRNA.

## Source data

Source Data Extended Data Fig. 1f Extended Data Fig. 1f statistical source data.

Source Data Extended Data Fig. 1g Extended Data Fig. 1g statistical source data.

Source Data Extended Data Fig. 2c Extended Data Fig. 2c statistical source data.

Source Data Extended Data Fig. 2f Extended Data Fig. 2f statistical source data.

Source Data Extended Data Fig. 5b Extended Data Fig. 5b statistical source data.

Source Data Extended Data Fig. 6b Extended Data Fig. 6b statistical source data.

Source Data Extended Data Fig. 6f Extended Data Fig. 6f statistical source data.

Source Data Extended Data Fig. 6g Extended Data Fig. 6g statistical source data.

Source Data Extended Data Fig. 6h Extended Data Fig. 6h statistical source data.

Source Data Extended Data Fig. 6i Extended Data Fig. 6i statistical source data.

Source Data Extended Data Fig. 7a Extended Data Fig. 7a statistical source data.

Source Data Extended Data Fig. 7b Extended Data Fig. 7b statistical source data.

Source Data Extended Data Fig. 7d Extended Data Fig. 7d statistical source data.

Source Data Extended Data Fig. 7f Extended Data Fig. 7f statistical source data.

Source Data Fig. 2c Fig. 2c statistical source data.

Source Data Fig. 2e Fig. 2e statistical source data.

Source Data Fig. 3d Fig. 3d statistical source data.

Source Data Fig. 3e Fig. 3e statistical source data.

Source Data Fig. 3f Fig. 3f statistical source data.

Source Data Fig. 4b Fig. 4b statistical source data.

Source Data Fig. 4d Fig. 4d statistical source data.

Source Data Fig. 5a Fig. 5a statistical source data.

Source Data Fig. 5b Fig. 5b statistical source data.

Source Data Fig. 5d Fig. 5d statistical source data.

Source Data Extended Data Fig. 1c Uncropped immunoblot Extended Data Fig. 1c.

Source Data Extended Data Fig. 1d Uncropped immunoblot Extended Data Fig. 1d.

Source Data Extended Data Fig. 1e Uncropped immunoblot Extended Data Fig. 1e.

Source Data Extended Data Fig. 5c Uncropped immunoblot Extended Data Fig. 5c.

Source Data Extended Data Fig. 6a Uncropped immunoblot Extended Data Fig. 6a.

Source Data Extended Data Fig. 6c Uncropped immunoblot Extended Data Fig. 6c.

Source Data Extended Data Fig. 6e Uncropped immunoblot Extended Data Fig. 6e.

Source Data Extended Data Fig. 7c Uncropped immunoblot Extended Data Fig. 7c.

Source Data Extended Data Fig. 7e Uncropped immunoblot Extended Data Fig. 7e.

Source Data Fig. 2a Uncropped immunoblot Fig. 2a.

Source Data Fig. 2b Uncropped immunoblot Fig. 2b.

Source Data Fig. 2d Uncropped immunoblot Fig. 2d.

Source Data Fig. 2f Uncropped immunoblot Fig. 2f.

Source Data Fig. 2g Uncropped immunoblot Fig. 2g.

Source Data Fig. 2h Uncropped immunoblot Fig. 2h.

Source Data Fig. 2i Uncropped immunoblot Fig. 2i.

Source Data Fig. 4c Uncropped immunoblot Fig. 4c.

Source Data Fig. 5c Uncropped immunoblot Fig. 5c.

Source Data Fig. 5e Uncropped immunoblot Fig. 5e.

Source Data Fig. 5f Uncropped immunoblot Fig. 5f.

Source Data Fig. 5g Uncropped immunoblot Fig. 5g.

Source Data Fig. 5h Uncropped immunoblot Fig. 5h.

Source Data Fig. 5i Uncropped immunoblot Fig. 5i.

Source Data Fig. 6a Uncropped immunoblot Fig. 6a.

Source Data Fig. 6b Uncropped immunoblot Fig. 6b.

Source Data Fig. 6c Uncropped immunoblot Fig. 6c.

Source Data Fig. 6d Uncropped immunoblot Fig. 6d.

Source Data Fig. 6e Uncropped immunoblot Fig. 6e.

Source Data Fig. 6f Uncropped immunoblot Fig. 6f.

Source Data Fig. 6g Uncropped immunoblot Fig. 6g.

Source Data Fig. 6h Uncropped immunoblot Fig. 6h.

## Data Availability

Deposition of raw and processed data from the scRNA-seq experiment are deposited in the NIH repository dbGaP with accession code phs004114.v1. Exome sequencing data will not be made publicly available as they contain information that might compromise research participant privacy/consent. Source data generated or analyzed during this study are included in the published article and its online supplementary files or are available from the corresponding authors A.S. (A.N.Spaan@umcutrecht.nl) and J.M. (janet.markle@vumc.org) within 4–8 weeks and upon reasonable request. Source data are provided with this paper.

## Extended data

**Extended Data Table 1:** Clinical laboratory findings, Patients 1 and 2 (P1 and P2, respectively) at indicated ages

	P1, age 23y	P2, age 26y	P2, age 28y	Reference range
CBC				
Hemoglobin	9.0 g dl^−1^ (L)	11.1 g/dL(L)	8.7 g/dL (L)	11.5 – 16.5 g/dL
Hematocrit			30.0 % (L)	37 – 47 %
Mean corpuscular Hb			28.9 g/dl (L)	30 – 35 g/dL
Total leukocyte count	37,000 /mm^3^ (H)	5,300 /mm^3^	7,520 /mm^3^	4,000 – 11,000 /mm^3^
Differential count				
Neutrophils	85.0 % (H)	47.0 %	69.3 %	40 – 70 %
Lymphocytes	4.0 % (L)	42.0 %	20.7 %	20 – 45 %
Monocytes		8.0 %	8.1 %	2–10 %
Eosinophils		3 %	1.8 %	1–6 %
Basophils			0.2 %	0–1 %
Other				
C-reactive protein		11.0 mg/L (H)	14.5 mg/L (H)	0–3 mg/L
Erythrocyte sed. rate			27 mm (H)	0 - 20 mm
DHR	normal			normal
Immunoglobulins				
IgA	3.44 g/L		2.03 g/L	0.7 – 4.0 g/L
IgG	11.30 g/L		14.20 g/L	5.49 – 15.84 g/L
IgM	1.12 g/L		1.18 g/L	0.4–2.3 g/L
IgE, serum	259.9 IU/mL		69.3 IU/mL	<378 IU/mL
Leukocyte subsets				
CD3^+^ T cells	1242 /uL		1139 /uL	780 - 3000 /uL
CD19^+^ B cells	264 /uL		128 /uL	64 - 820 /uL
CD16^+^CD56^+^ NK cells	164 /uL		62 /uL (L)	100 - 1200 /uL

**Extended Data Table 2:** Gene expression for several NF-kB targets and signaling components are similar in healthy donor and patient samples

	Gene	Fold change (OTULIN-R57C vs HC; Nanostring))	p-value (OTULIN-R57C vs HC; Nanostring)	p_val_adj PBMC (OTULIN-R57C vs HC, scRNA-seq)	p_val_adj monocytes (OTULIN-R57C vs HC; scRNA-seq)	NF-κB target literature ref. PMID
NF-κB target genes	*CXCL10*	1.556951329	0.268347059	1	0.117575456	19901067
	*ETS1*	1.126322299	0.518454014	1	0.932383721	20368503
	*ICAM1*	1.160638329	0.601115937	1	0.462901597	1347304
	*IRF1*	0.74273376	0.577588851	1	0.381018106	33761354
	*TNFAIP3 (A20)*	1.152234393	0.386633723	1	0.289171257	29940800
	*IL6R*	0.981533844	0.922044222	1	0.629310936	2809509
	*CCL3*	1.04332144	0.948794101	0.681143992	0.388448185	32006659
	*CDKN1A*	1.042202003	0.874611047	1	0.986405691	37969056
	*BTK*	1.043696816	0.729290487	1	0.679507141	15117762
	*CCR7*	1.011046605	0.945936751	1	0.76520929	23219392
	*HIF1A*	n/a	n/a	1	0.95141258	30386909
NF-κB signal transducers	*IKBKG (NEMO)*	1.058929429	0.772530696	1	0.899331015	
	*NFKBIA (IκBα)*	0.8549984	0.264164926	1	0.126454032	
	*IKBKB (IKKβ)*	1.152397803	0.211916047	1	0.943685351	
	*CHUK (IKKα)*	1.37875539	0.10290128	1	0.912983574	
	*NFIA (p65)*	n/a	n/a	1	0.126454032	

Fold change and p-values for NF-κB target genes (upper panel) and NF-κB signal transducers (lower panel) in OTULIN-R57C patients versus healthy controls (HC), as assessed by two independent platforms are shown. Left-most column lists NF-κB-related genes. Second and third columns show results from probe-based mRNA assay (Nanostring) assessment of whole blood samples from R57C OTULIN patients vs. healthy controls, as described in the Methods, p-values from two-tailed student’s t-tests. Fourth and fifth columns show output from scRNA-seq differential expression analysis output comparing PBMCs and monocytes, respectively, from R57C OTULIN patients vs. healthy controls, as described in the Methods, and p_val_adj values indicate adjusted p-values for multiple testing and based on Bonferroni correction using total features in the dataset.

**Extended Data Table 3:** Top 15 DEGs identified comparing healthy controls and OTULIN-R57C patients’ PBMCs

Gene	Fold Change	p_val_adj
*RPS26*	7.35607693	1.16E-215
*PIK3CA*	2.2218683	3.69E-18
*CFL2*	2.19834876	0.002215524
*CASZ1*	2.13179935	1.21E-12
*MARCKS*	2.12440375	1.58E-05
*IL8*	2.1074303	8.22E-08
*AC010275.1*	2.01963697	1.44E-13
*TCF7*	2.01856246	9.54E-13
*IRF2BP2*	2.01079219	5.64E-13
*JAK3*	1.99873672	8.61E-19
*IL1B*	1.964142	0.000467313
*AQP9*	1.93114147	7.32E-07
*DOT1L*	1.89795409	2.67E-08
*UBASH3B*	1.89781408	8.95E-31
*BATF*	1.88983015	4.48E-05

The top 15 DEGs from scRNA-seq differential expression analysis output comparing R57C OTULIN patients’ vs. three healthy controls’ PBMCs. Genes were ranked by fold change values. P_val_adj values indicate adjusted p-values for multiple testing and based on Bonferroni correction using total features in the dataset.

## Code availability

Processed Cell Ranger matrix files as well as R scripts for all analyses presented are available at https://github.com/JanetMarkleLaboratory/phs004114.git.
