# Supplementary material for: Pyoderma gangrenosum caused by the molecular uncoupling of OTULIN catalytic activity and LUBAC binding
Source: Nat Immunol. 2026 Jun 15;27(8):1619–32. doi: 10.1038/s41590-026-02568-6 (PMC13414579; doi:10.1038/s41590-026-02568-6)
Supplement: Supplementary file 1 — Reporting Summary [file 41590_2026_2568_MOESM1_ESM.pdf]

Corresponding author(s): Andras N. Spaan, Janet G. Markle

Last updated by author(s): Apr 16, 2026

Reporting Summary

Nature Portfolio wishes to improve the reproducibility of the work that we publish. This form provides structure for consistency and transparency in reporting. For further information on Nature Portfolio policies, see our Editorial Policies and the Editorial Policy Checklist.

Statistics

For all statistical analyses, confirm that the following items are present in the figure legend, table legend, main text, or Methods section.

n/a	Confirmed	
☐	☒	The exact sample size (*n*) for each experimental group/condition, given as a discrete number and unit of measurement
☐	☒	A statement on whether measurements were taken from distinct samples or whether the same sample was measured repeatedly
☐	☒	The statistical test(s) used AND whether they are one- or two-sided<br>*Only common tests should be described solely by name; describe more complex techniques in the Methods section.*
☒	☐	A description of all covariates tested
☐	☒	A description of any assumptions or corrections, such as tests of normality and adjustment for multiple comparisons
☐	☒	A full description of the statistical parameters including central tendency (e.g. means) or other basic estimates (e.g. regression coefficient) AND variation (e.g. standard deviation) or associated estimates of uncertainty (e.g. confidence intervals)
☐	☒	For null hypothesis testing, the test statistic (e.g. *F*, *t*, *r*) with confidence intervals, effect sizes, degrees of freedom and *P* value noted<br>*Give P values as exact values whenever suitable.*
☒	☐	For Bayesian analysis, information on the choice of priors and Markov chain Monte Carlo settings
☒	☐	For hierarchical and complex designs, identification of the appropriate level for tests and full reporting of outcomes
☒	☐	Estimates of effect sizes (e.g. Cohen's *d*, Pearson's *r*), indicating how they were calculated

Our web collection on statistics for biologists contains articles on many of the points above.

Software and code

Policy information about availability of computer code

Data collection	Data were collected using standard, commercially-available software. Details are provided in the Materials and Methods section of the manuscript.
Data analysis	Data from single cell RNA sequencing were analyzed in R using open-source, published R packages as described in Material and Methods. Processed cell ranger matrix files as well as R scripts for all analyses presented are available at https://github.com/JanetMarkleLaboratory/phs004114.git

For manuscripts utilizing custom algorithms or software that are central to the research but not yet described in published literature, software must be made available to editors and reviewers. We strongly encourage code deposition in a community repository (e.g. GitHub). See the Nature Portfolio guidelines for submitting code & software for further information.

Data

Policy information about availability of data

All manuscripts must include a data availability statement. This statement should provide the following information, where applicable:

- Accession codes, unique identifiers, or web links for publicly available datasets
- A description of any restrictions on data availability
- For clinical datasets or third party data, please ensure that the statement adheres to our policy

Raw and processed data from the scRNA-seq experiment are deposited in the NIH repository dbGaP with accession #phs004114.v1

Research involving human participants, their data, or biological material

Policy information about studies with human participants or human data. See also policy information about sex, gender (identity/presentation), and sexual orientation and race, ethnicity and racism.

Reporting on sex and gender	Sex of all three patients reported in this manuscript are described in Figure 1 (pedigrees) and in the Supplemental Material (case reports).
Reporting on race, ethnicity, or other socially relevant groupings	The ethnicity of all three patients reported in this manuscript are described in the Results and Supplemental Material (case reports).
Population characteristics	Covariate-relevant population statistics of the patients (such as age, genotype, ancestry) are described in the Results and Supplemental Material (case reports).
Recruitment	Patients were recruited under approved IRB protocols at the Rockefeller University and Vanderbilt University Medical Center. This is described in the Materials and Methods.
Ethics oversight	Patients were recruited under approved IRB protocols at the Rockefeller University (protocols JCA-0698 and JCA-0695) and Vanderbilt University Medical Center (protocol 200412).

Note that full information on the approval of the study protocol must also be provided in the manuscript.

Field-specific reporting

Please select the one below that is the best fit for your research. If you are not sure, read the appropriate sections before making your selection.

☒ Life sciences ☐ Behavioural & social sciences ☐ Ecological, evolutionary & environmental sciences

For a reference copy of the document with all sections, see nature.com/documents/nr-reporting-summary-flat.pdf

Life sciences study design

All studies must disclose on these points even when the disclosure is negative.

Sample size	This study centers on the identification of a new, very rare genetic disease in 3 affected patients. As such, sample size was not pre-determined. The group of patients reported (n=3) were identified as sharing a novel rare genetic mutation.
Data exclusions	No data were excluded.
Replication	Whenever possible, biological and technical replicates were performed and the data resulting from these replicates are presented in the figures and described in the figure legends.
Randomization	No randomization was applied for this study
Blinding	No blinding was applied for this study

Reporting for specific materials, systems and methods

We require information from authors about some types of materials, experimental systems and methods used in many studies. Here, indicate whether each material, system or method listed is relevant to your study. If you are not sure if a list item applies to your research, read the appropriate section before selecting a response.

Materials & experimental systems

n/a	Involved in the study	
☐	☒	Antibodies
☐	☒	Eukaryotic cell lines
☒	☐	Palaeontology and archaeology
☒	☐	Animals and other organisms
☐	☒	Clinical data
☒	☐	Dual use research of concern
☒	☐	Plants

Methods

n/a	Involved in the study	
☒	☐	ChIP-seq
☐	☒	Flow cytometry
☒	☐	MRI-based neuroimaging

Antibodies

Antibodies used	All antibodies used are described in the Materials and Methods and Supplementary Tables.
Validation	All antibodies used are commercially available, validated as specific by manufacturers, and published in multiple prior studies. In addition, biological controls were used in our experiments to validate specificity whenever possible.

Eukaryotic cell lines

Policy information about cell lines and Sex and Gender in Research

Cell line source(s)	HEK293T (ATCC, female), HEK293 (ATCC, female), N/TERT-1 keratinocytes (generated by Jim Rheinwald PMC85304, shared by Ellen H van den Bogaard PMC5605545)
Authentication	Independent authentication was not performed as cells were obtained from a trusted commercial supplier and/or have been published in reputable peer-reviewed journals (e.g. PMC85304 and PMC5605545 for N/TERT-1 keratinocytes). As part of this study we created CRISPR-Cas9 knockout cell lines. The protocols and gRNAs used for CRISPR-Cas9 knockout are described in the Materials and Methods and sequence data is provided in Supplemental Tables.
Mycoplasma contamination	All cell lines tested negative for mycoplasma contamination
Commonly misidentified lines (See ICLAC register)	No commonly misidentified cell lines were used in this study

Clinical data

Policy information about clinical studies

All manuscripts should comply with the ICMJE guidelines for publication of clinical research and a completed CONSORT checklist must be included with all submissions.

Clinical trial registration	This is not a clinical trial
Study protocol	This is not a clinical trial
Data collection	This is not a clinical trial
Outcomes	This is not a clinical trial

Plants

Seed stocks	This is not a plant study
Novel plant genotypes	This is not a plant study
Authentication	This is not a plant study

Flow Cytometry

Plots

Confirm that:

- [ ] The axis labels state the marker and fluorochrome used (e.g. CD4-FITC).
- [ ] The axis scales are clearly visible. Include numbers along axes only for bottom left plot of group (a 'group' is an analysis of identical markers).
- [ ] All plots are contour plots with outliers or pseudocolor plots.
- [ ] A numerical value for number of cells or percentage (with statistics) is provided.

Methodology

Sample preparation	Sample preparation for cytometry by time-of-flight is described in detail in the Materials and Methods
Instrument	Helios CyTOF 3.0 (Fluidigm)
Software	Software used for multidimensional analyses of CyTOF data is described in the Materials and Methods
Cell population abundance	No sorting was applied
Gating strategy	Unsupervised clustering was performed as described in the Materials and Methods

☐ Tick this box to confirm that a figure exemplifying the gating strategy is provided in the Supplementary Information.
