# Supplementary figures and images for: Pyoderma gangrenosum caused by the molecular uncoupling of OTULIN catalytic activity and LUBAC binding
Source: Nat Immunol. 2026 Jun 15;27(8):1619–32. doi: 10.1038/s41590-026-02568-6 (PMC13414579; doi:10.1038/s41590-026-02568-6)

Extended Data Figure 1C

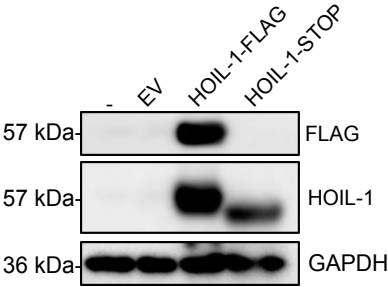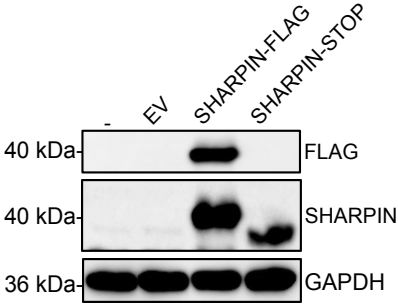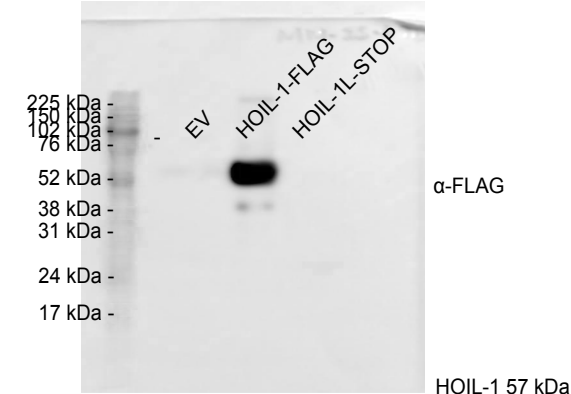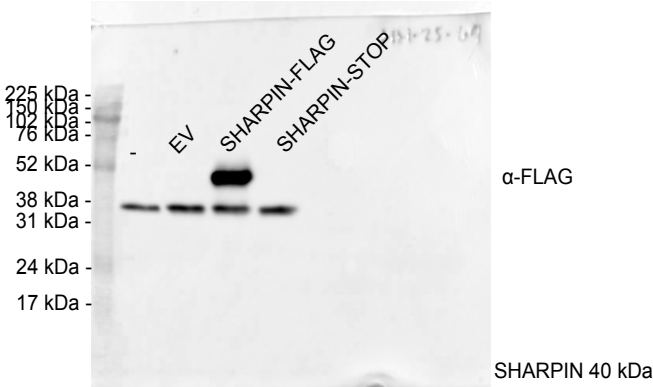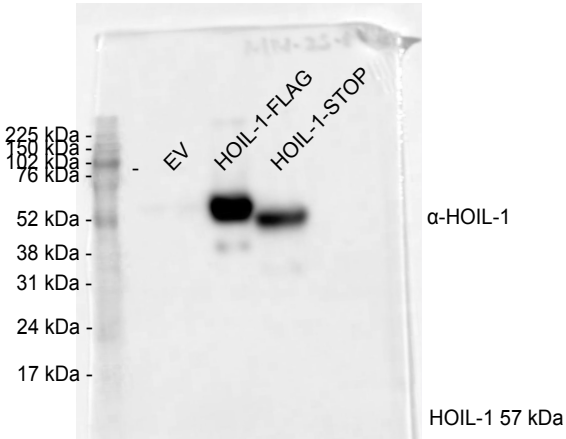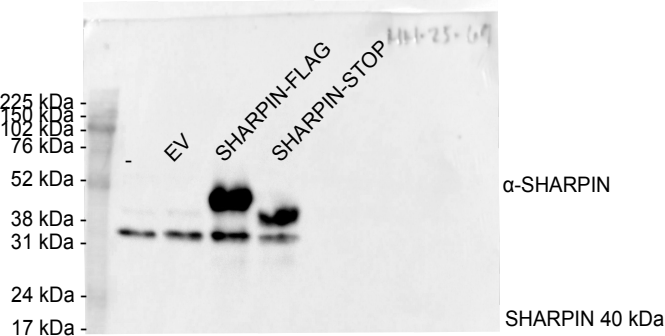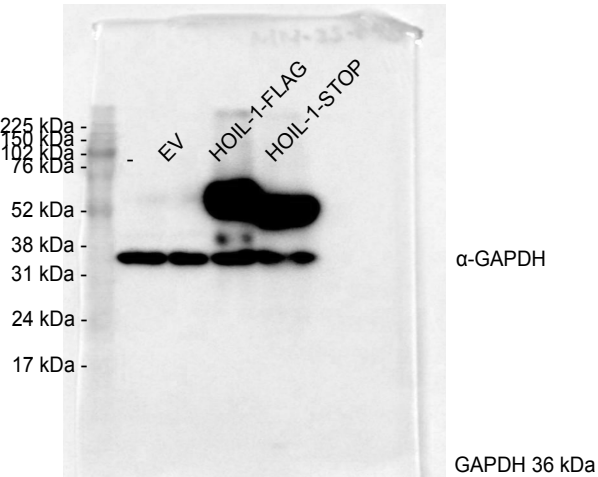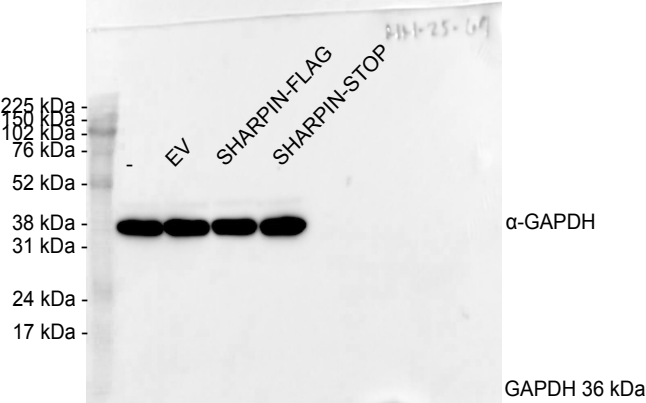

Supplement: Supplementary file 30 — Uncropped immunoblot Extended Data Fig. 1c. [file 41590_2026_2568_MOESM30_ESM.pdf]

Extended Data Figure 1E

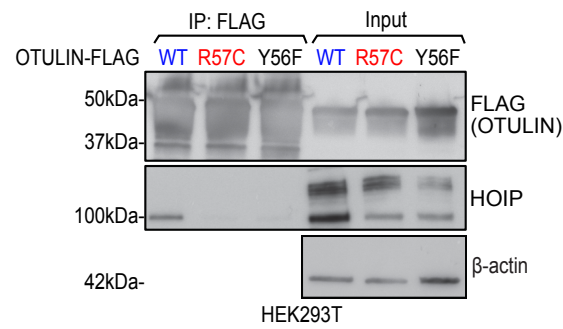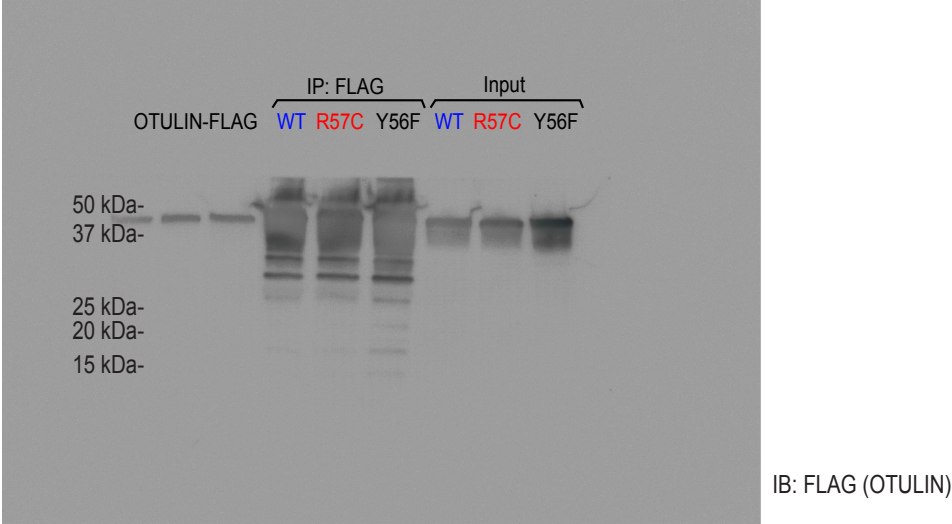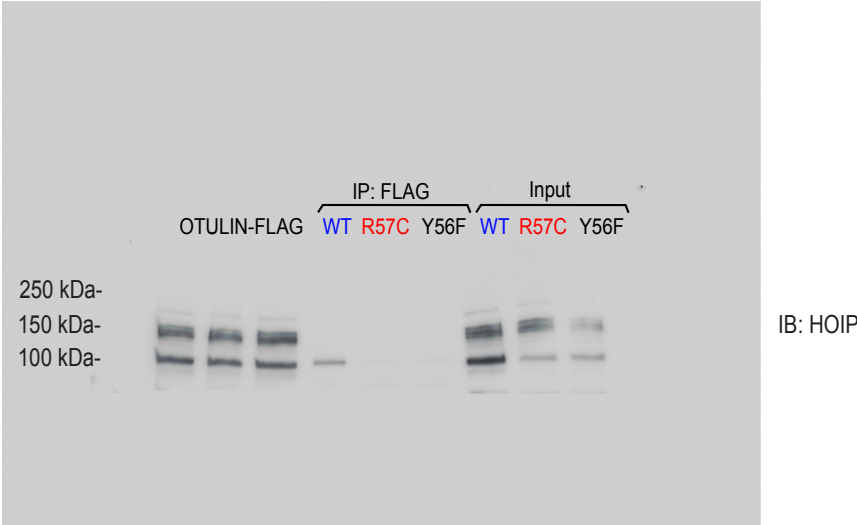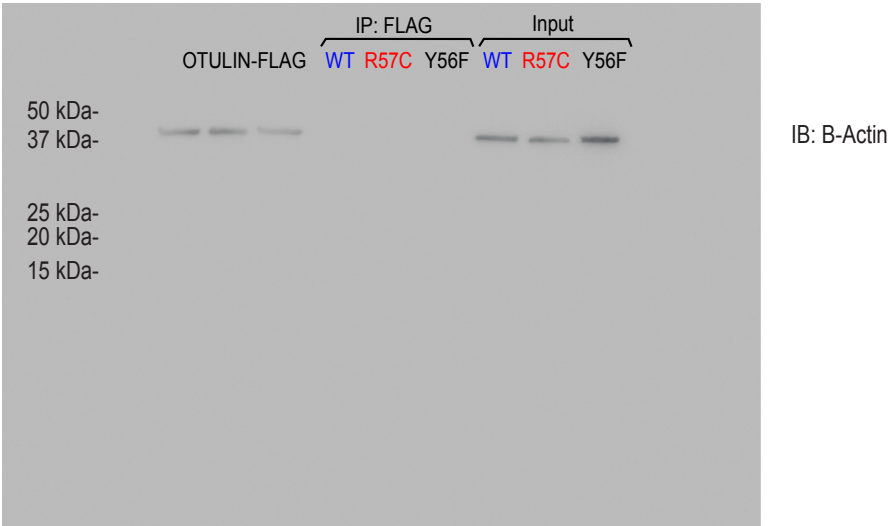

Supplement: Supplementary file 32 — Uncropped immunoblot Extended Data Fig. 1e. [file 41590_2026_2568_MOESM32_ESM.pdf]

Extended Data Figure 5C

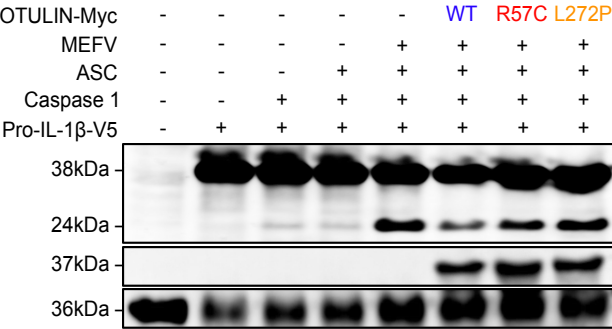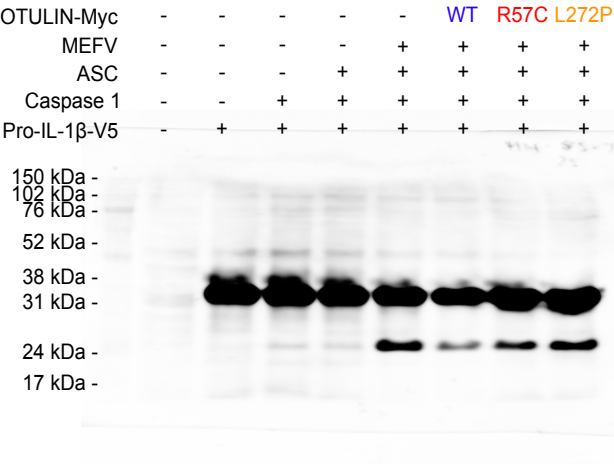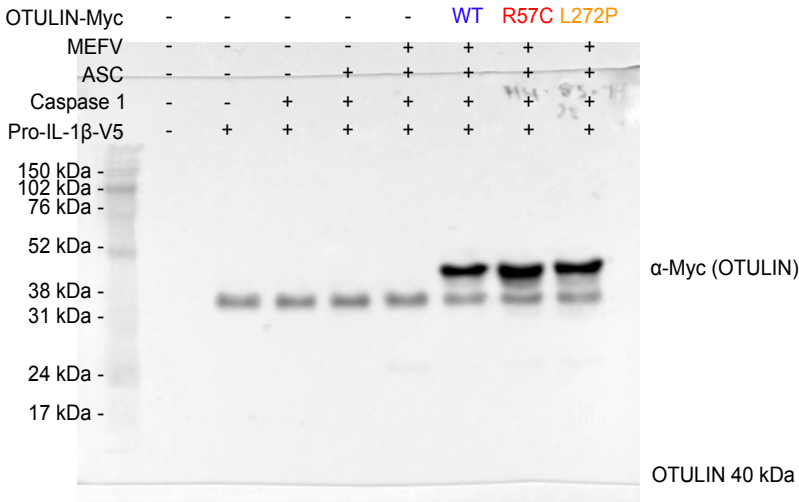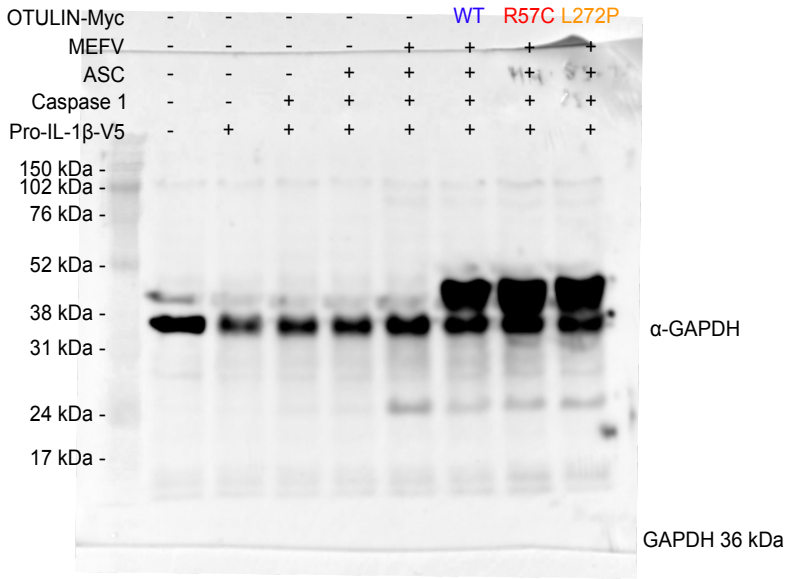

Supplement: Supplementary file 33 — Uncropped immunoblot Extended Data Fig. 5c. [file 41590_2026_2568_MOESM33_ESM.pdf]

Extended Data Figure 6A

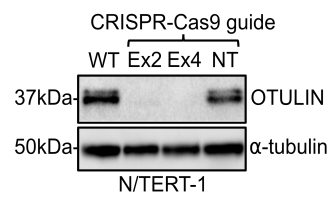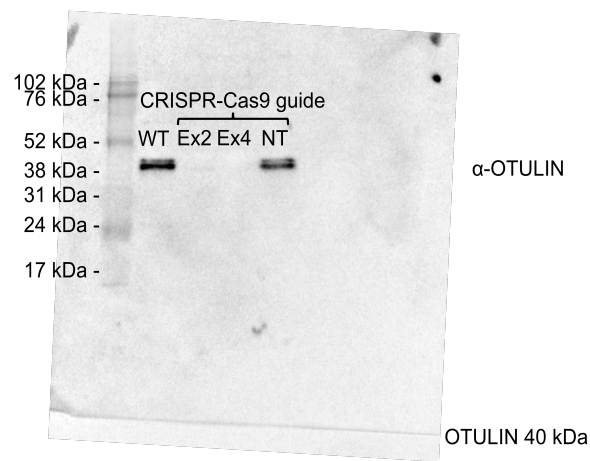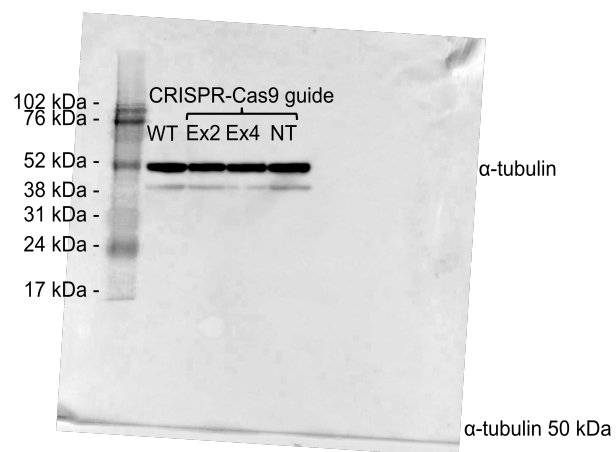

Supplement: Supplementary file 34 — Uncropped immunoblot Extended Data Fig. 6a. [file 41590_2026_2568_MOESM34_ESM.pdf]

Extended Data Figure 6C

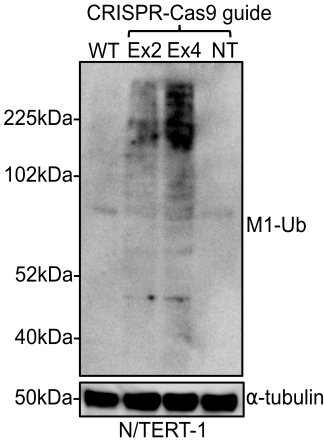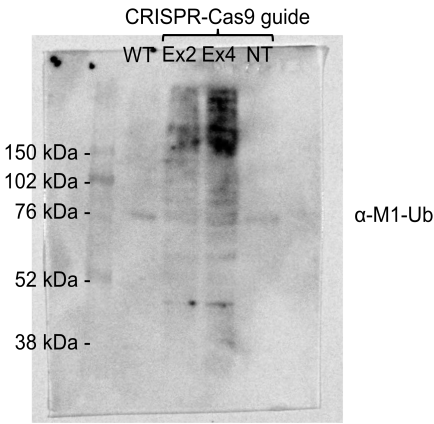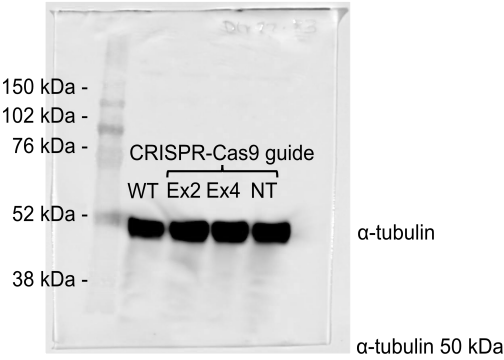

Supplement: Supplementary file 35 — Uncropped immunoblot Extended Data Fig. 6c. [file 41590_2026_2568_MOESM35_ESM.pdf]

Extended Data Figure 6E

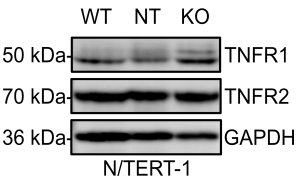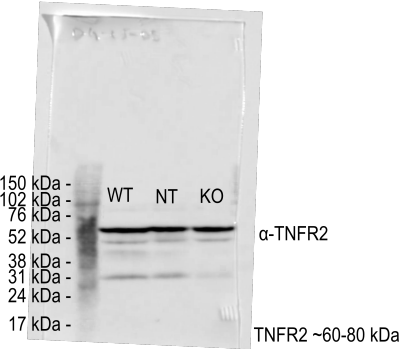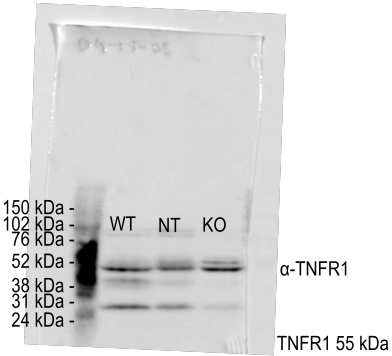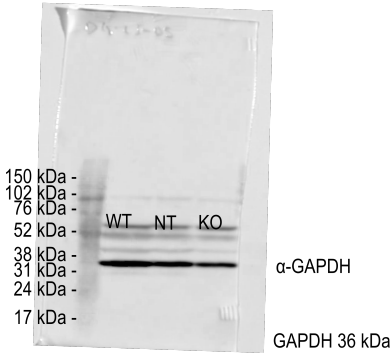

Supplement: Supplementary file 36 — Uncropped immunoblot Extended Data Fig. 6e. [file 41590_2026_2568_MOESM36_ESM.pdf]

Extended Data Figure 7C

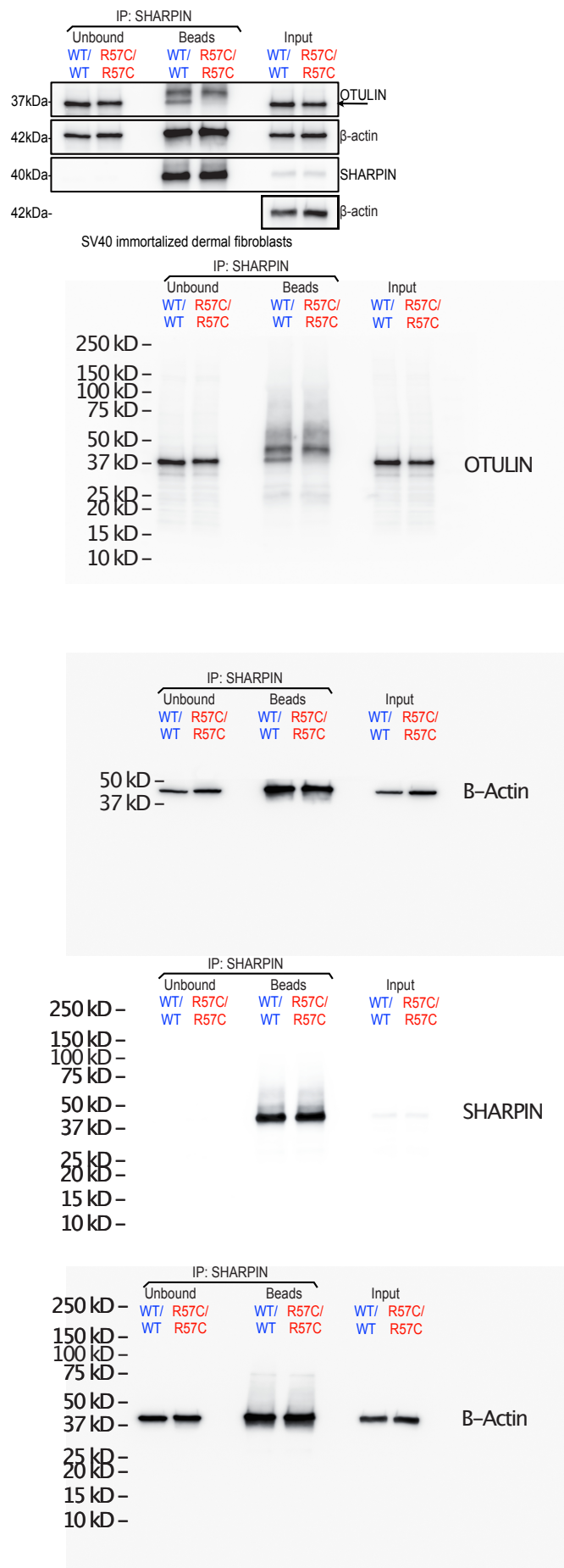

Supplement: Supplementary file 37 — Uncropped immunoblot Extended Data Fig. 7c. [file 41590_2026_2568_MOESM37_ESM.pdf]

Extended Data Figure 7E

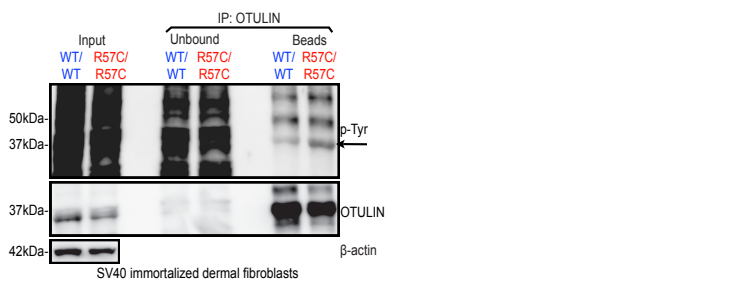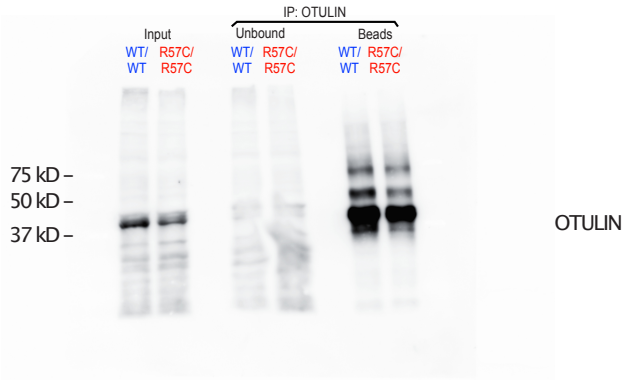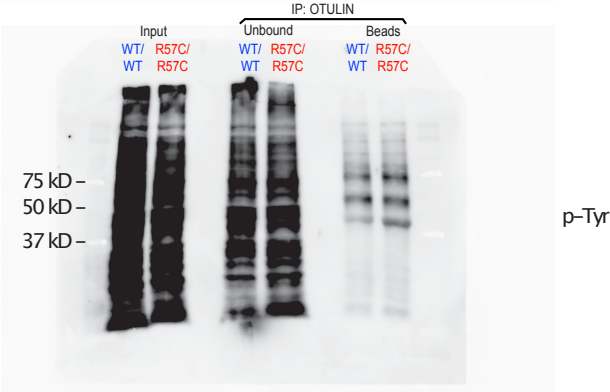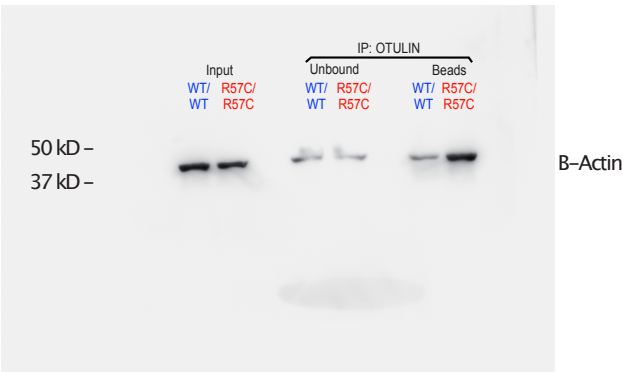

Supplement: Supplementary file 38 — Uncropped immunoblot Extended Data Fig. 7e. [file 41590_2026_2568_MOESM38_ESM.pdf]

Figure 2A

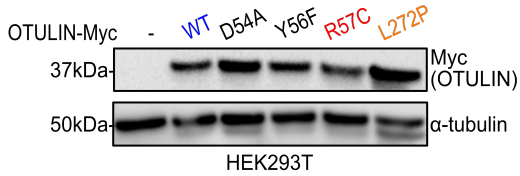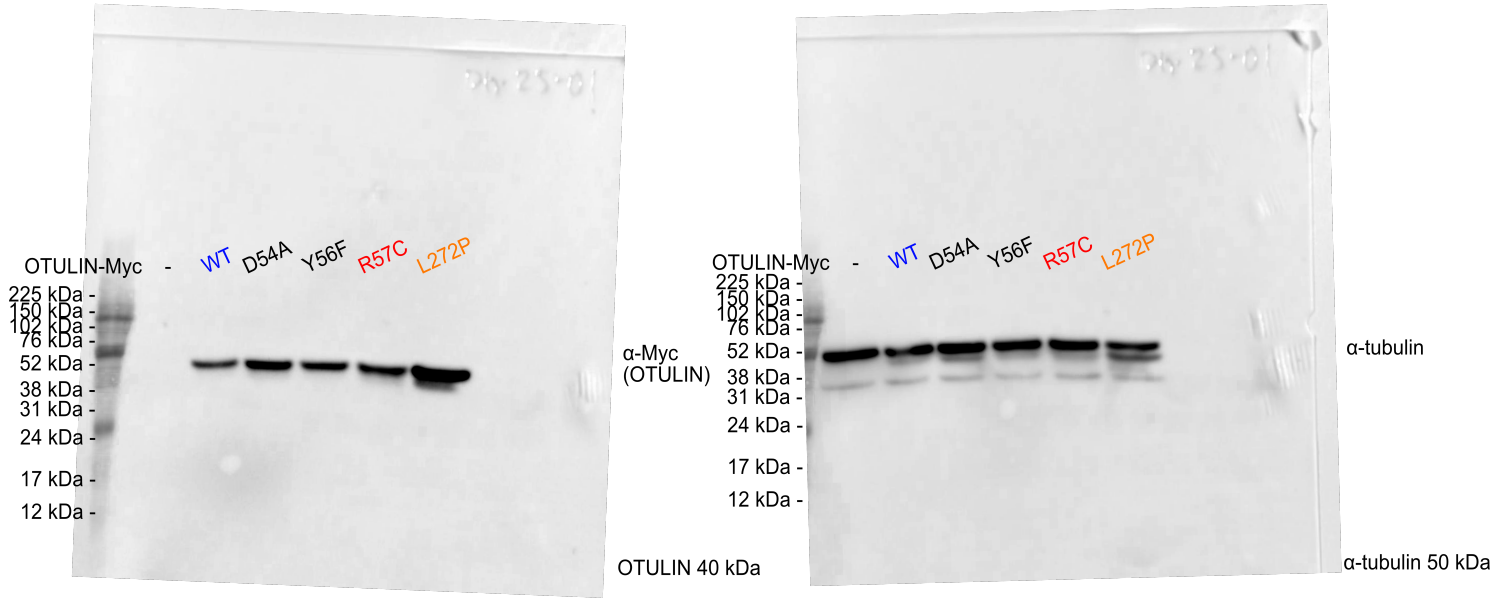

Supplement: Supplementary file 39 — Uncropped immunoblot Fig. 2a. [file 41590_2026_2568_MOESM39_ESM.pdf]

Figure 2B

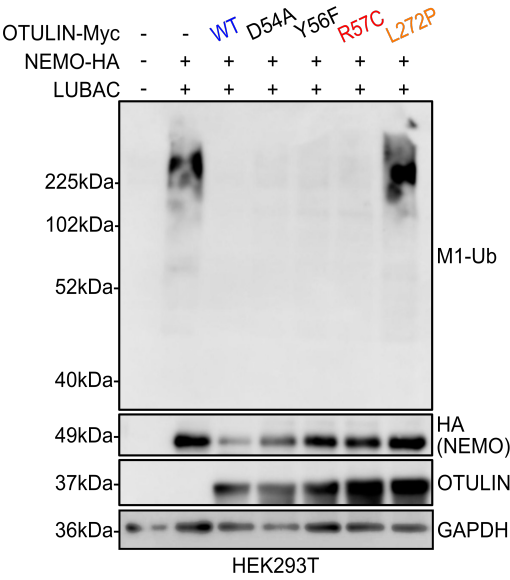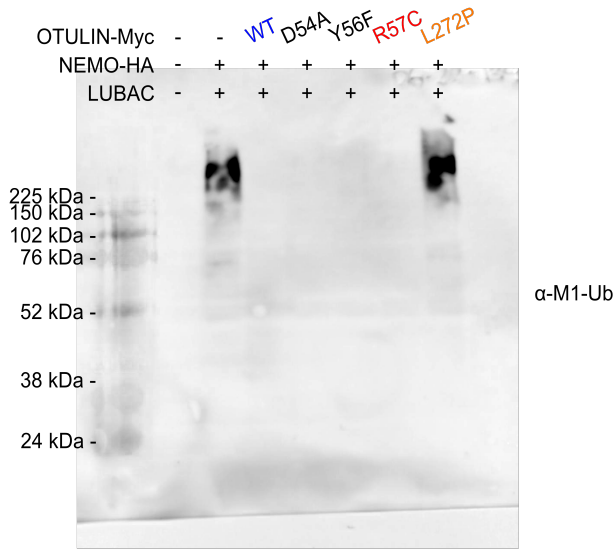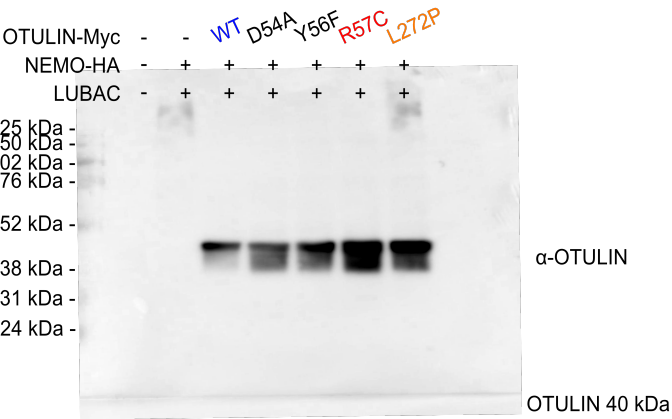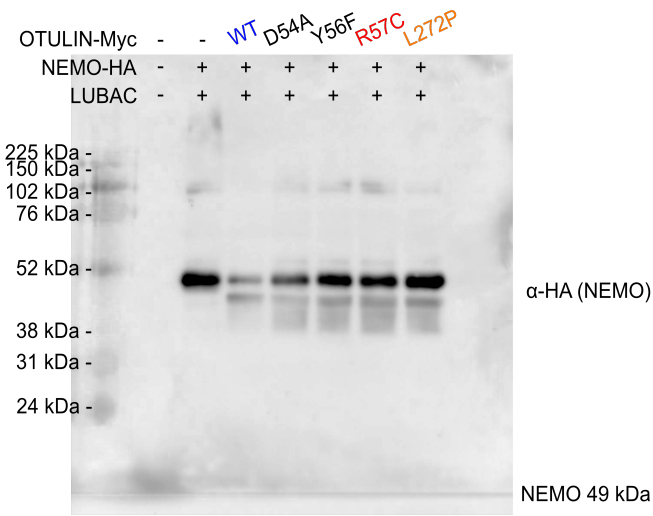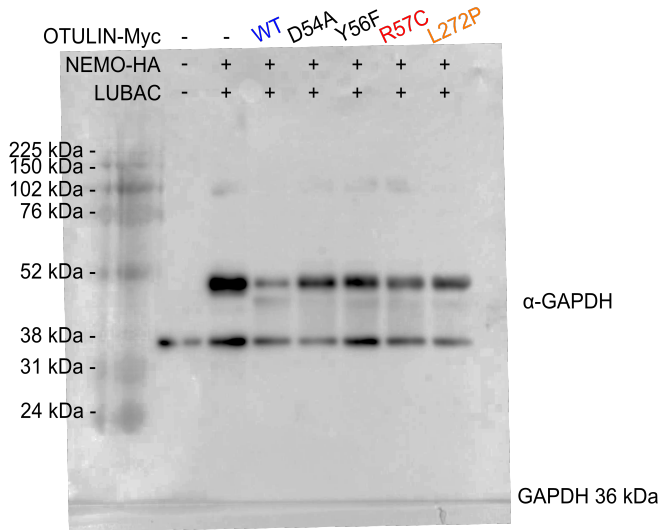

Supplement: Supplementary file 40 — Uncropped immunoblot Fig. 2b. [file 41590_2026_2568_MOESM40_ESM.pdf]

Figure 2D

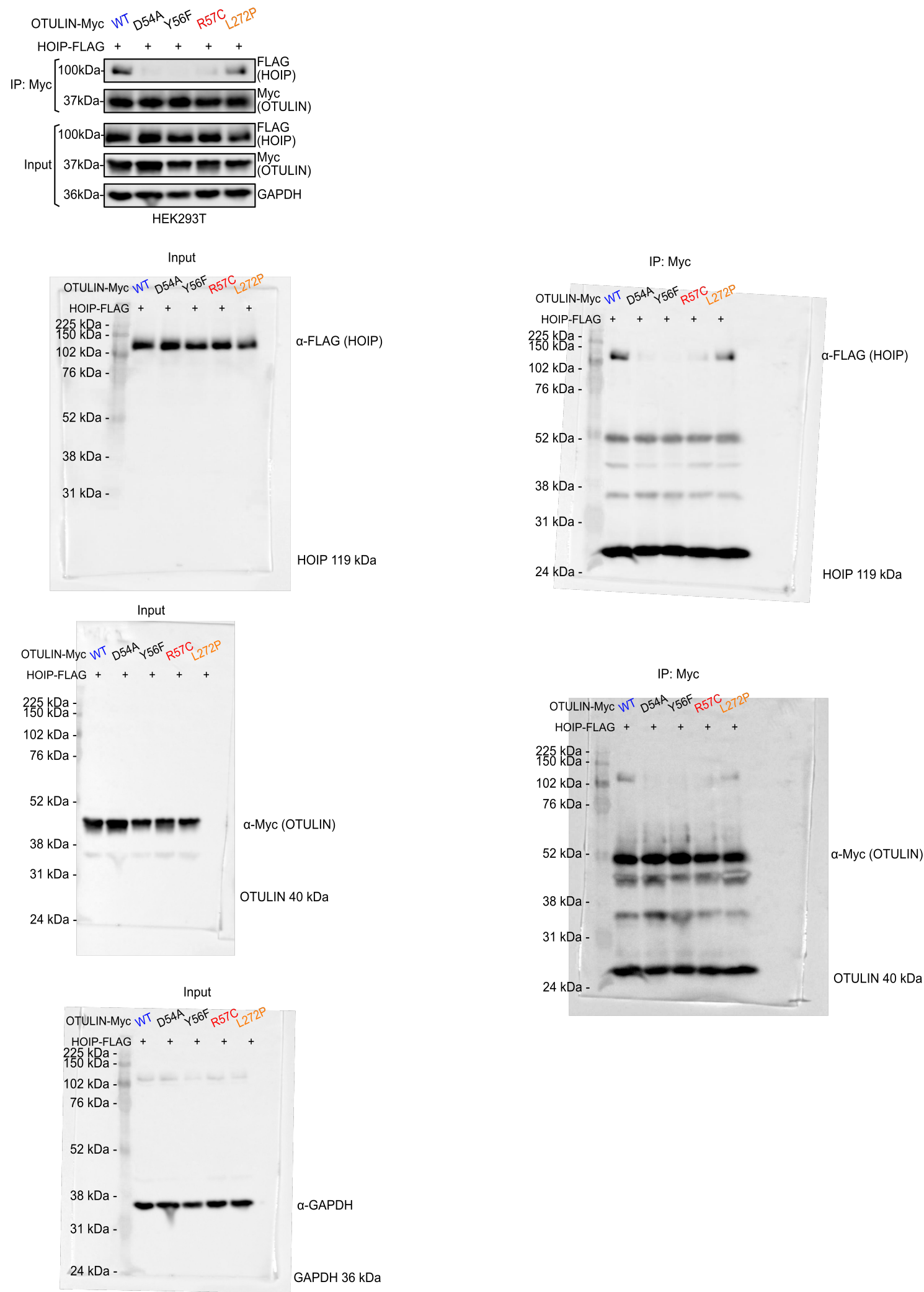

Supplement: Supplementary file 41 — Uncropped immunoblot Fig. 2d. [file 41590_2026_2568_MOESM41_ESM.pdf]

Figure 2F

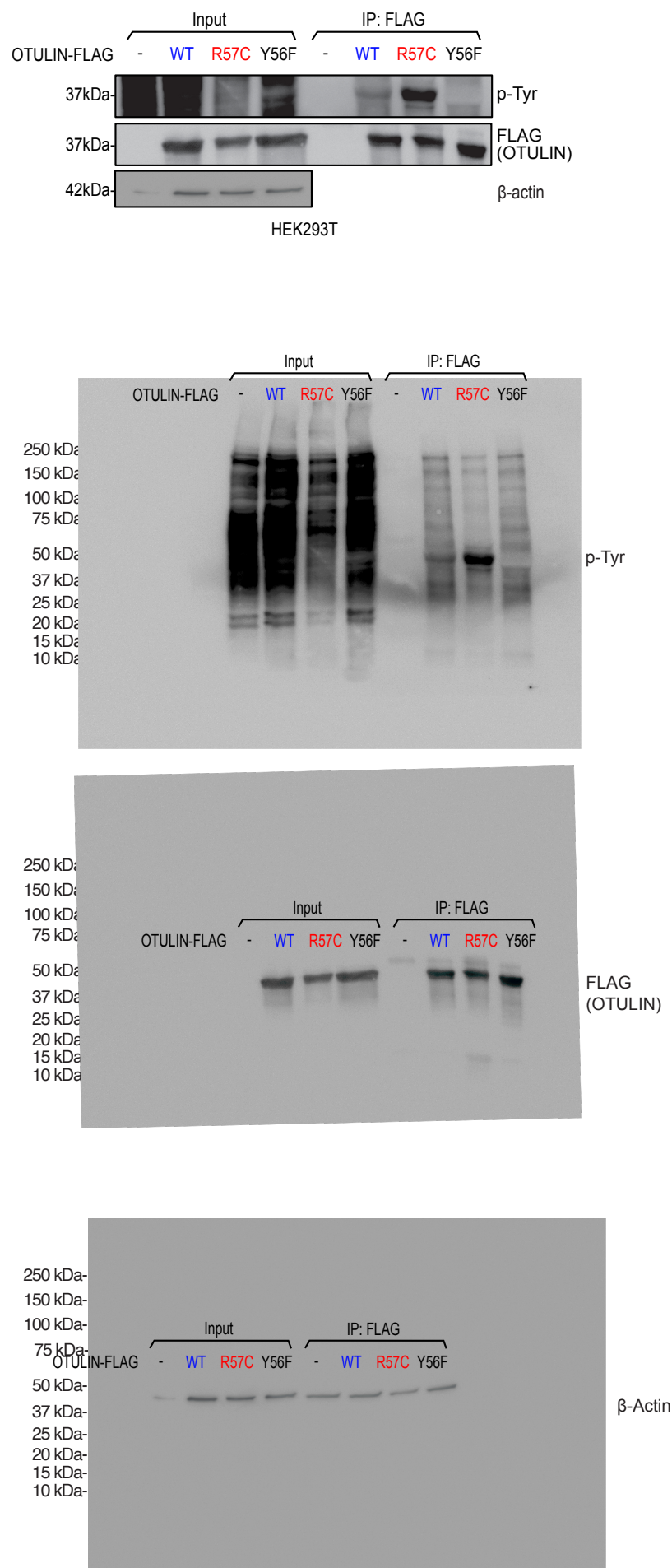

Supplement: Supplementary file 42 — Uncropped immunoblot Fig. 2f. [file 41590_2026_2568_MOESM42_ESM.pdf]

Figure 2G

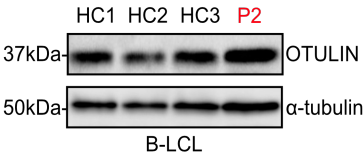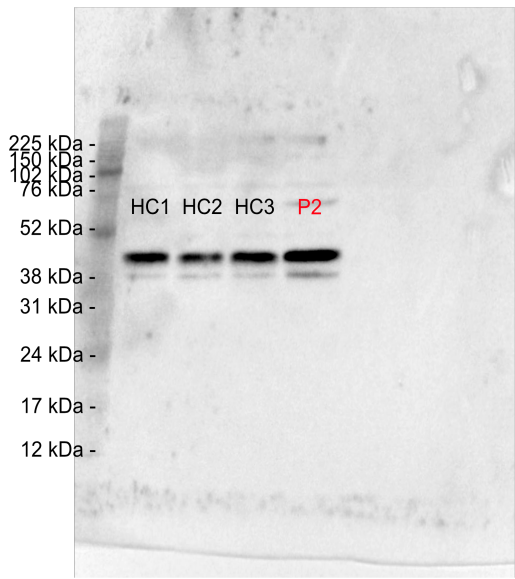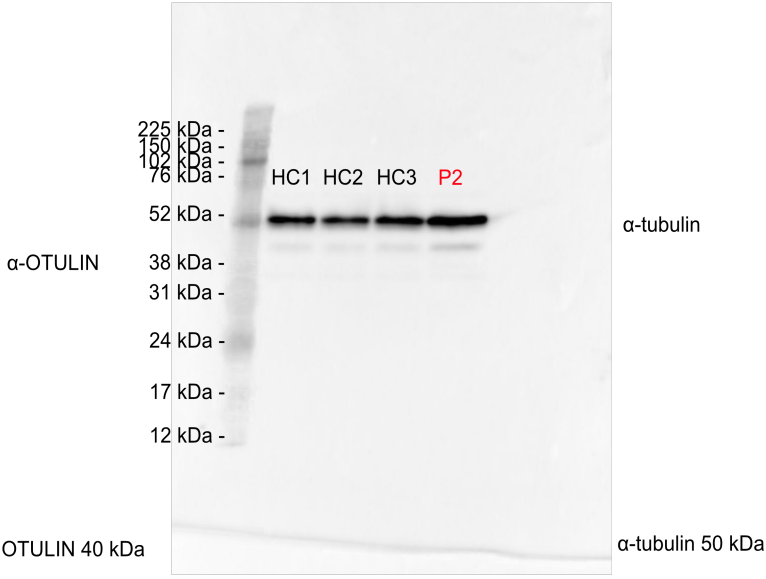

Supplement: Supplementary file 43 — Uncropped immunoblot Fig. 2g. [file 41590_2026_2568_MOESM43_ESM.pdf]

**Figure 2H**

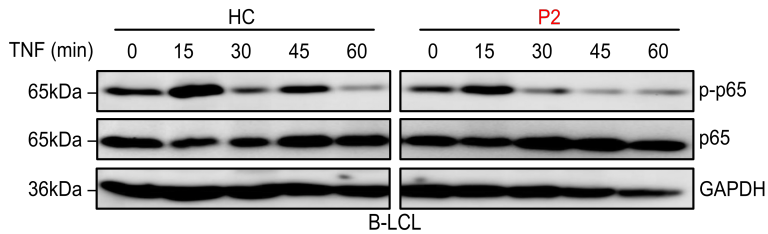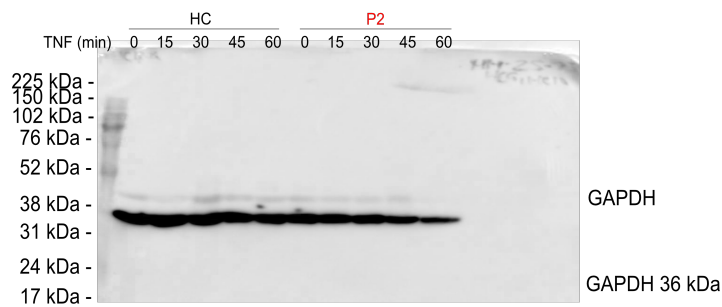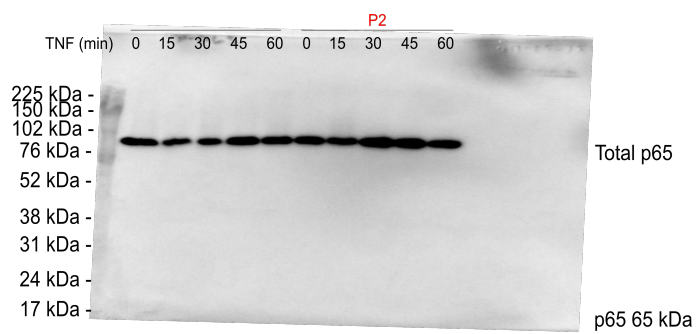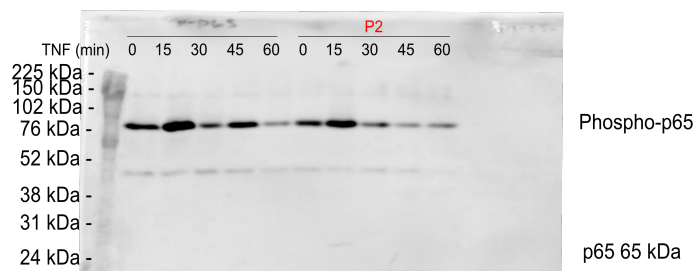

Supplement: Supplementary file 44 — Uncropped immunoblot Fig. 2h. [file 41590_2026_2568_MOESM44_ESM.pdf]

Figure 2I

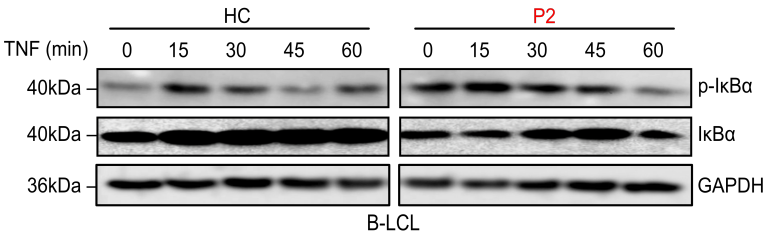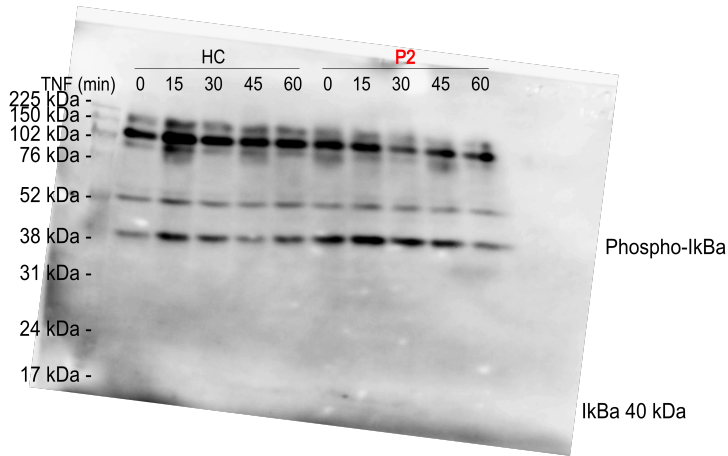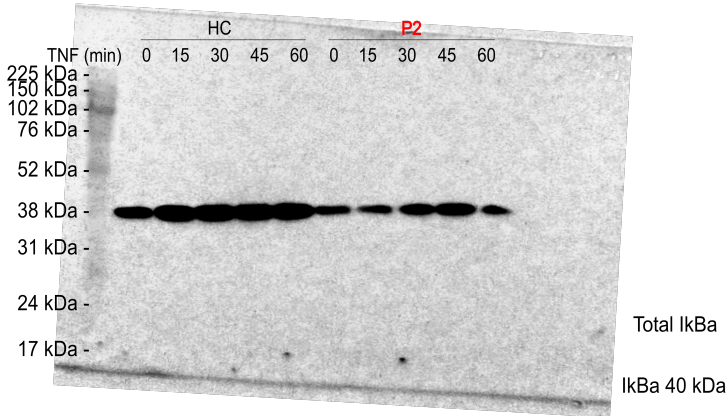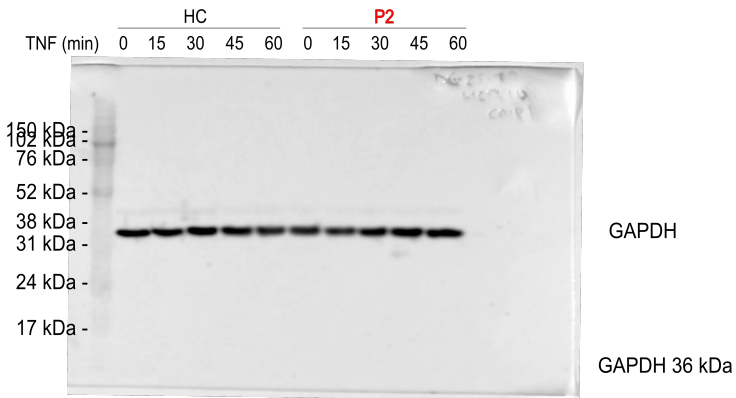

Supplement: Supplementary file 45 — Uncropped immunoblot Fig. 2i. [file 41590_2026_2568_MOESM45_ESM.pdf]

Figure 4C

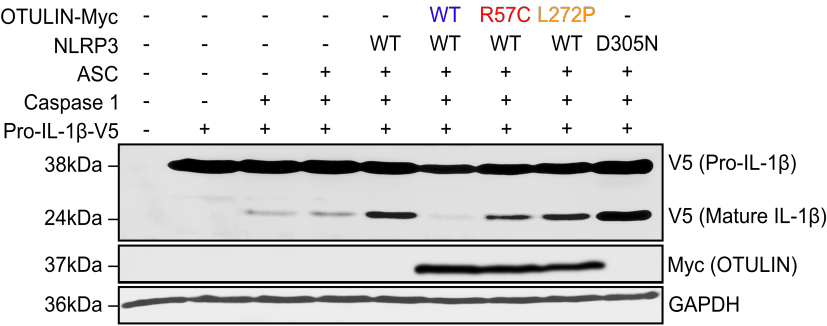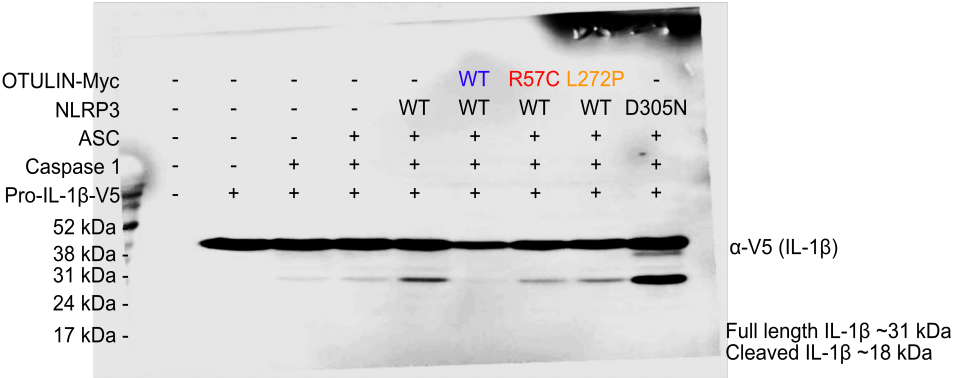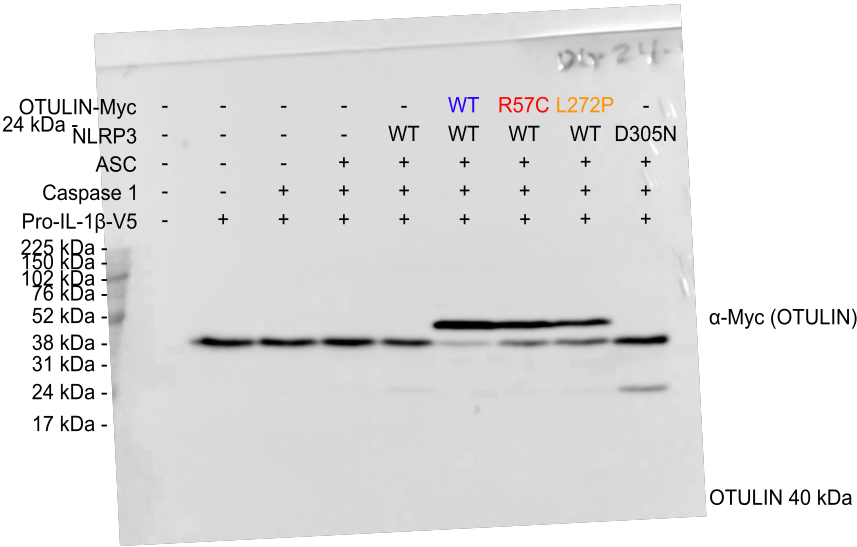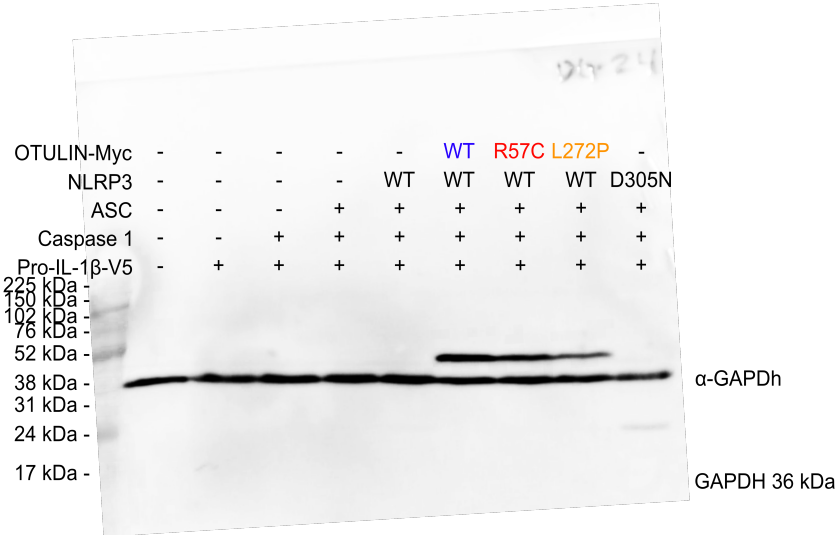

Supplement: Supplementary file 46 — Uncropped immunoblot Fig. 4c. [file 41590_2026_2568_MOESM46_ESM.pdf]

Figure 5C

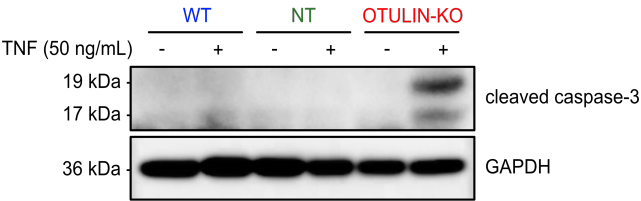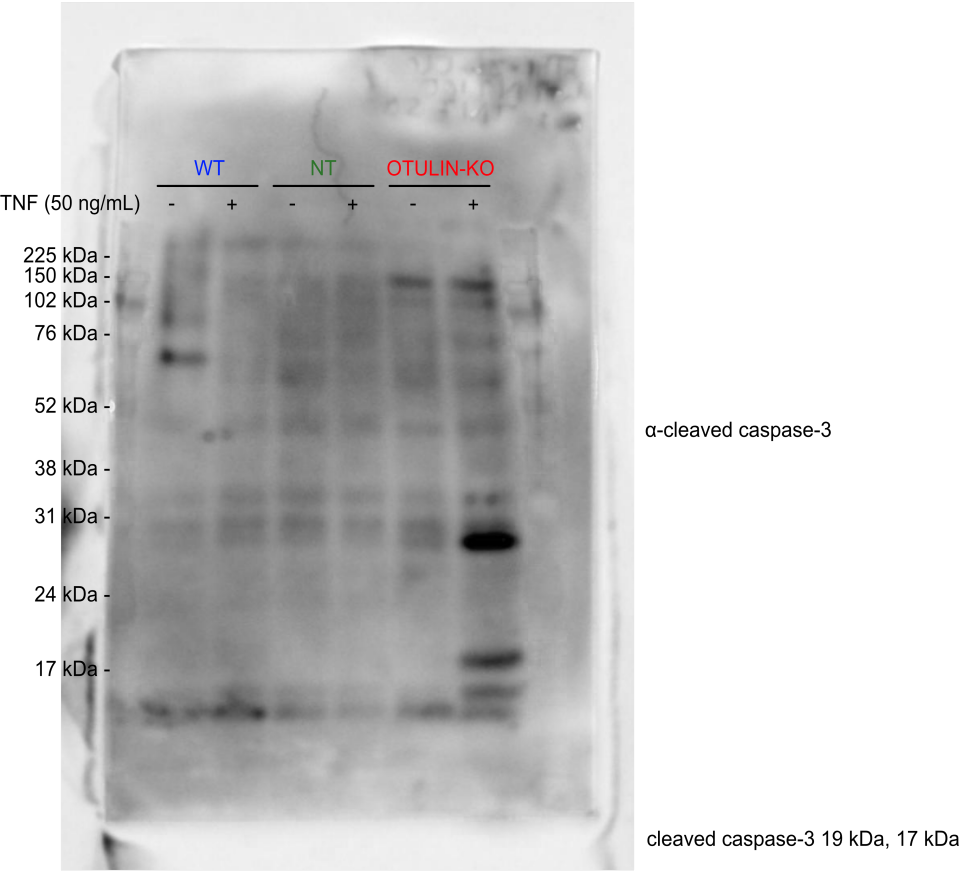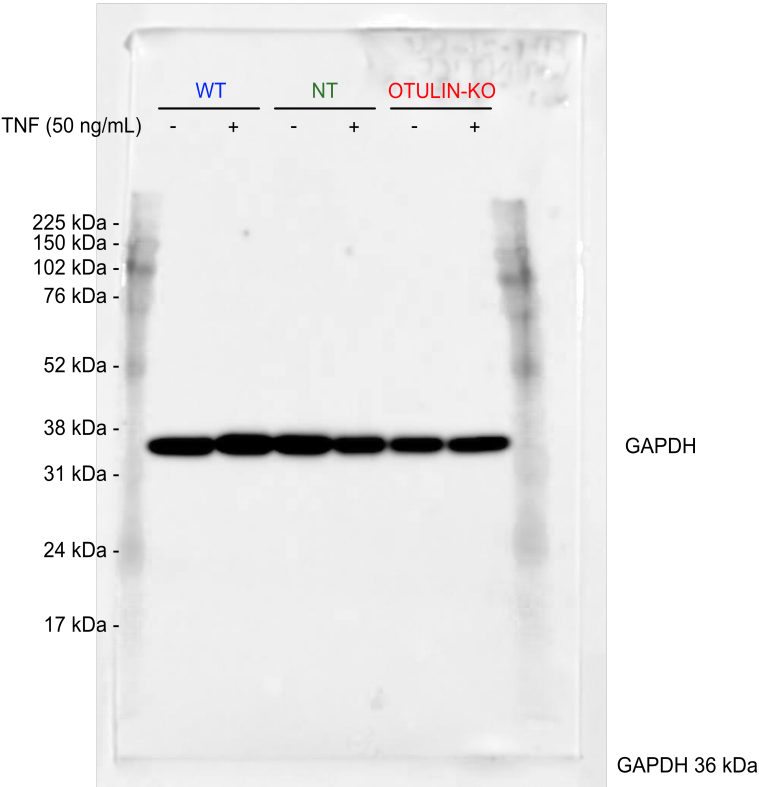

Supplement: Supplementary file 47 — Uncropped immunoblot Fig. 5c. [file 41590_2026_2568_MOESM47_ESM.pdf]

Figure 5E

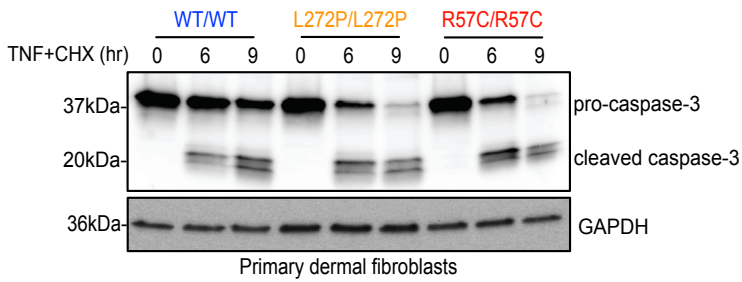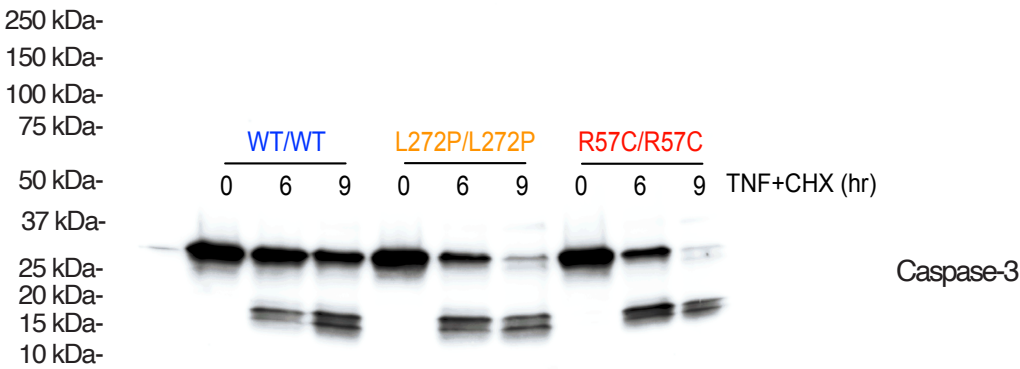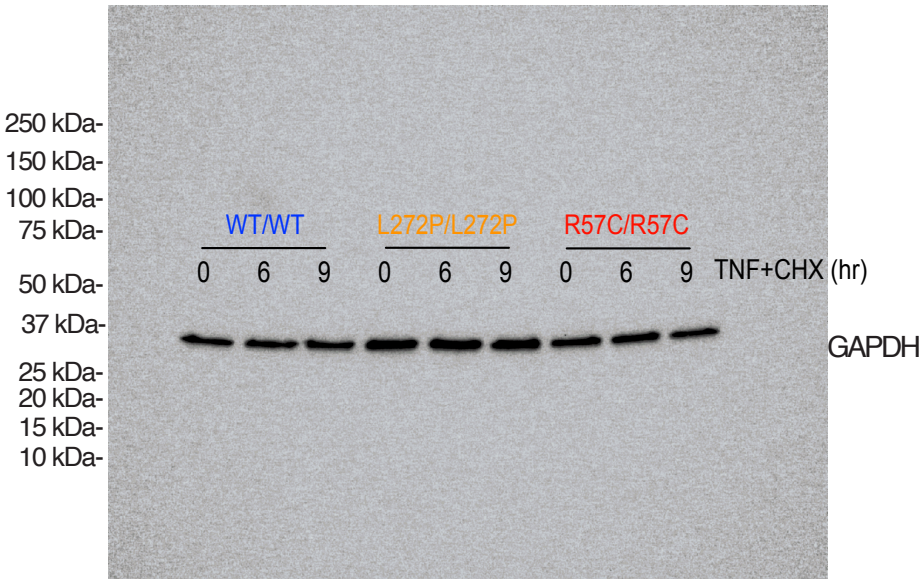

Supplement: Supplementary file 48 — Uncropped immunoblot Fig. 5e. [file 41590_2026_2568_MOESM48_ESM.pdf]

Figure 5F

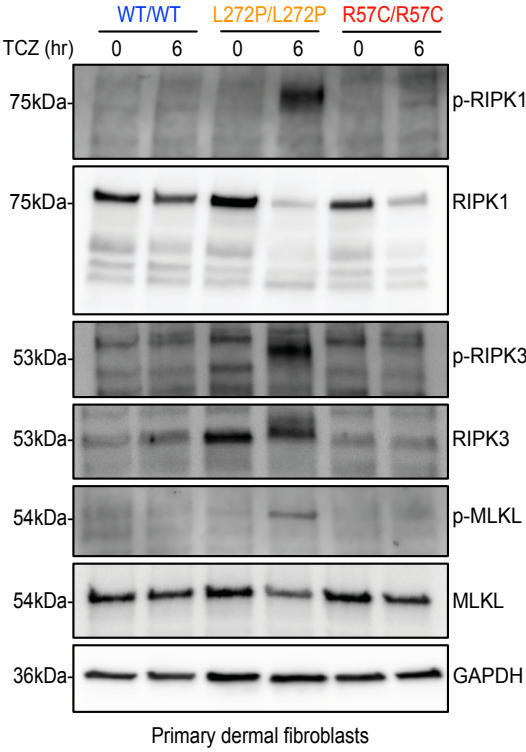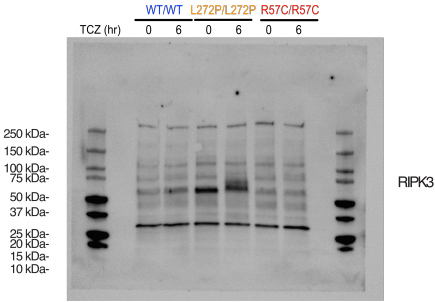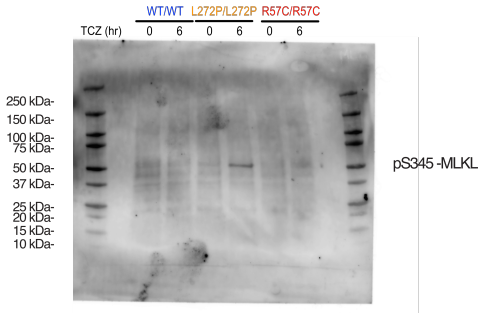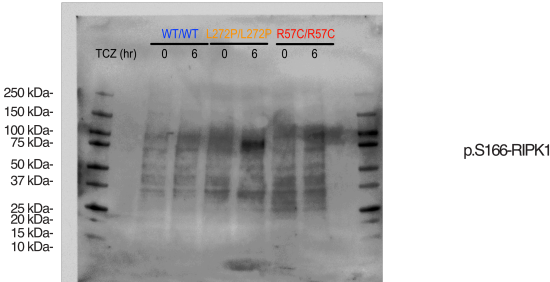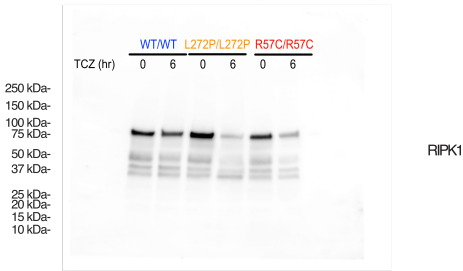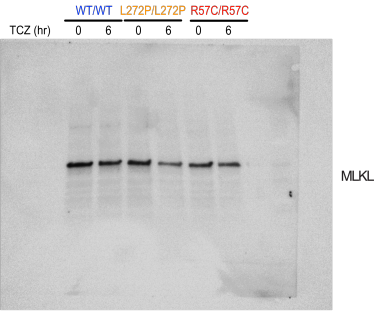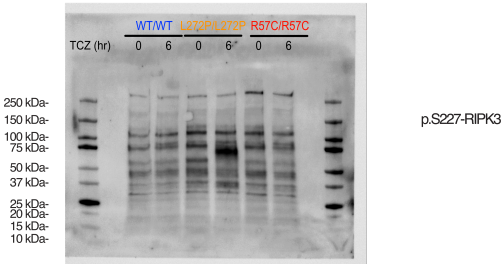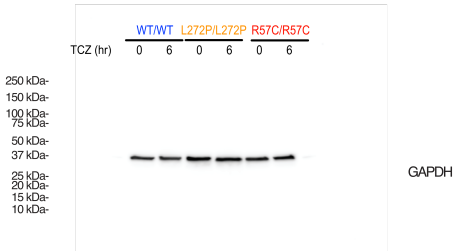

Supplement: Supplementary file 49 — Uncropped immunoblot Fig. 5f. [file 41590_2026_2568_MOESM49_ESM.pdf]

Figure 5G

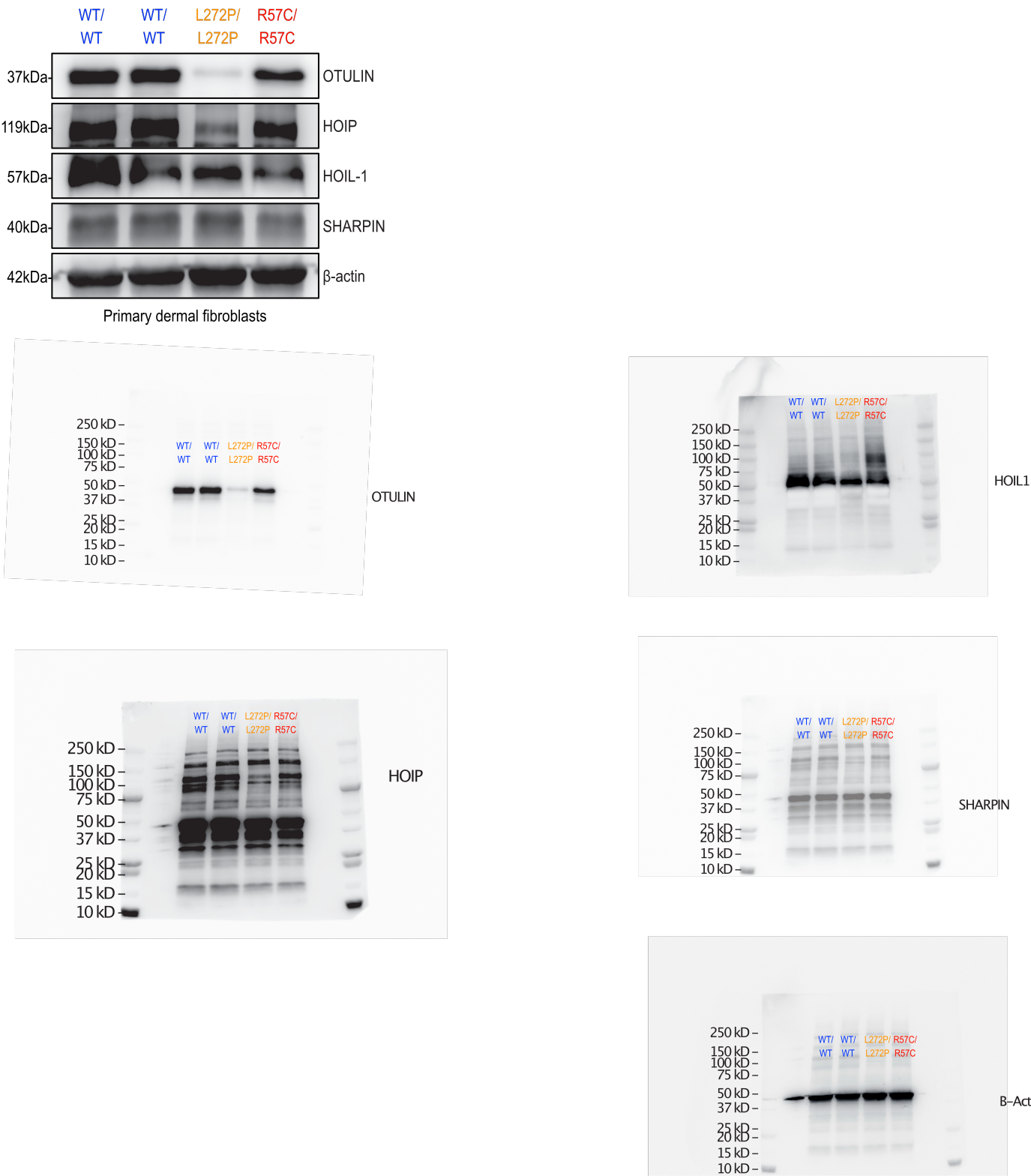

Supplement: Supplementary file 50 — Uncropped immunoblot Fig. 5g. [file 41590_2026_2568_MOESM50_ESM.pdf]

Figure 5H

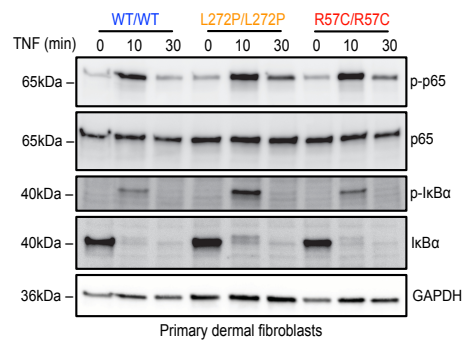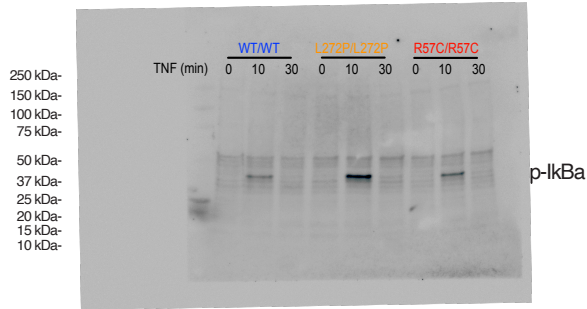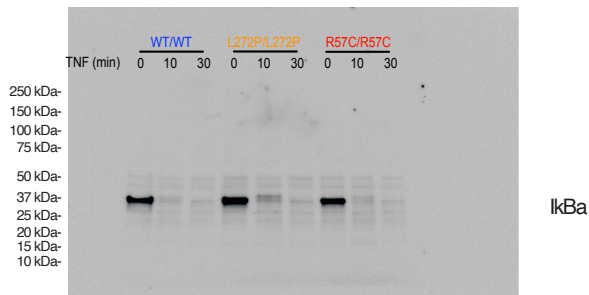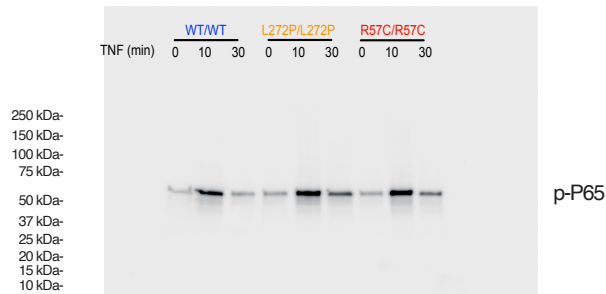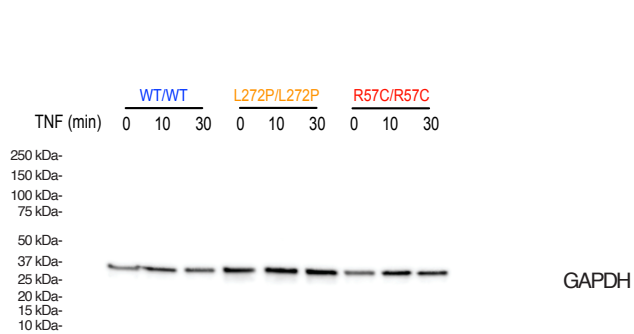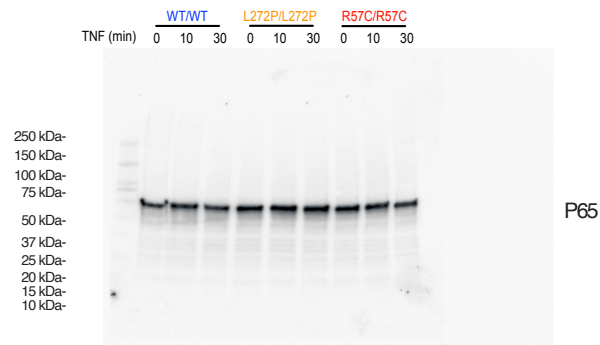

Supplement: Supplementary file 51 — Uncropped immunoblot Fig. 5h. [file 41590_2026_2568_MOESM51_ESM.pdf]

**Figure 5l**

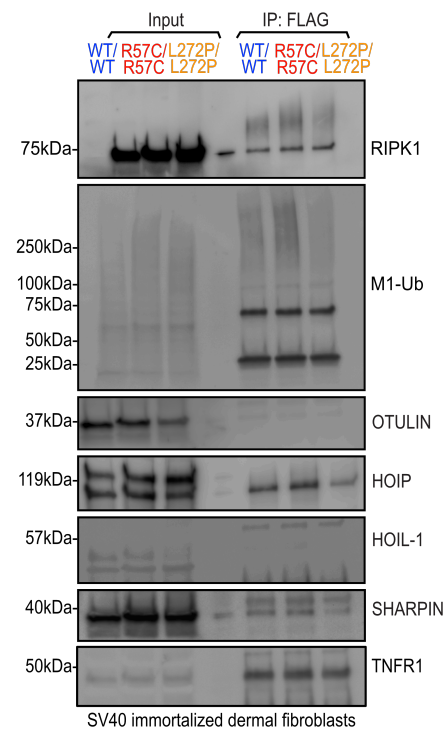

SV40 immortalized dermal fibroblasts

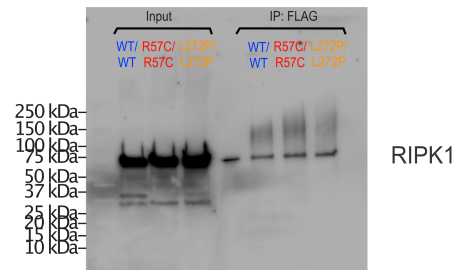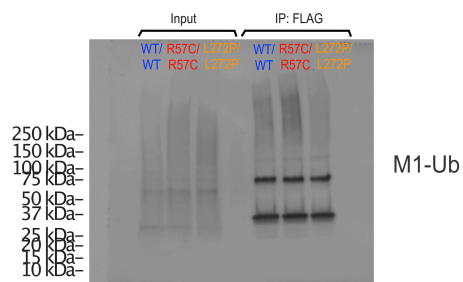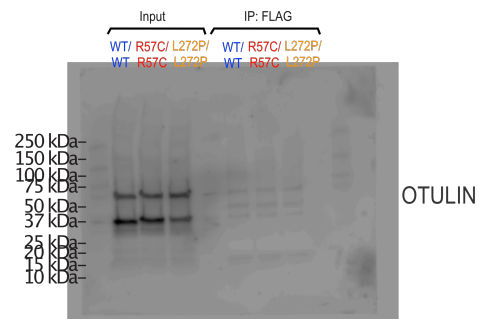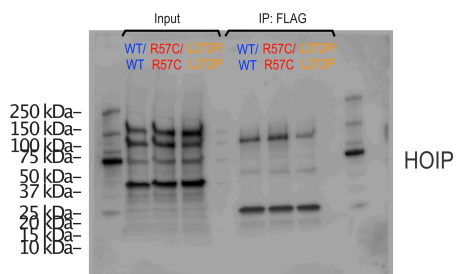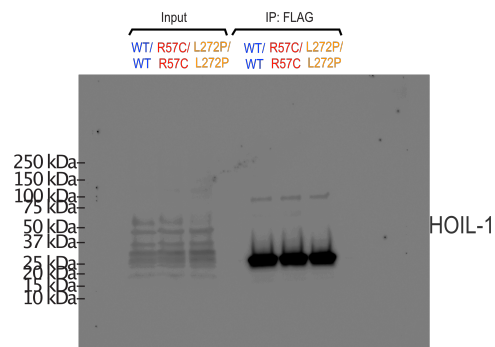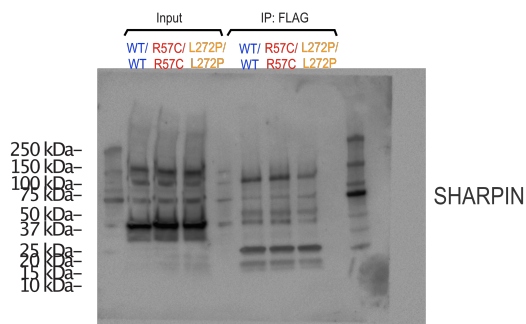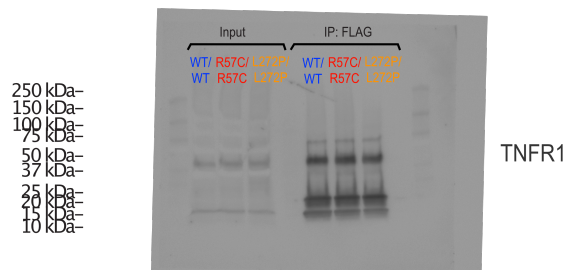

Supplement: Supplementary file 52 — Uncropped immunoblot Fig. 5i. [file 41590_2026_2568_MOESM52_ESM.pdf]

Figure 6A

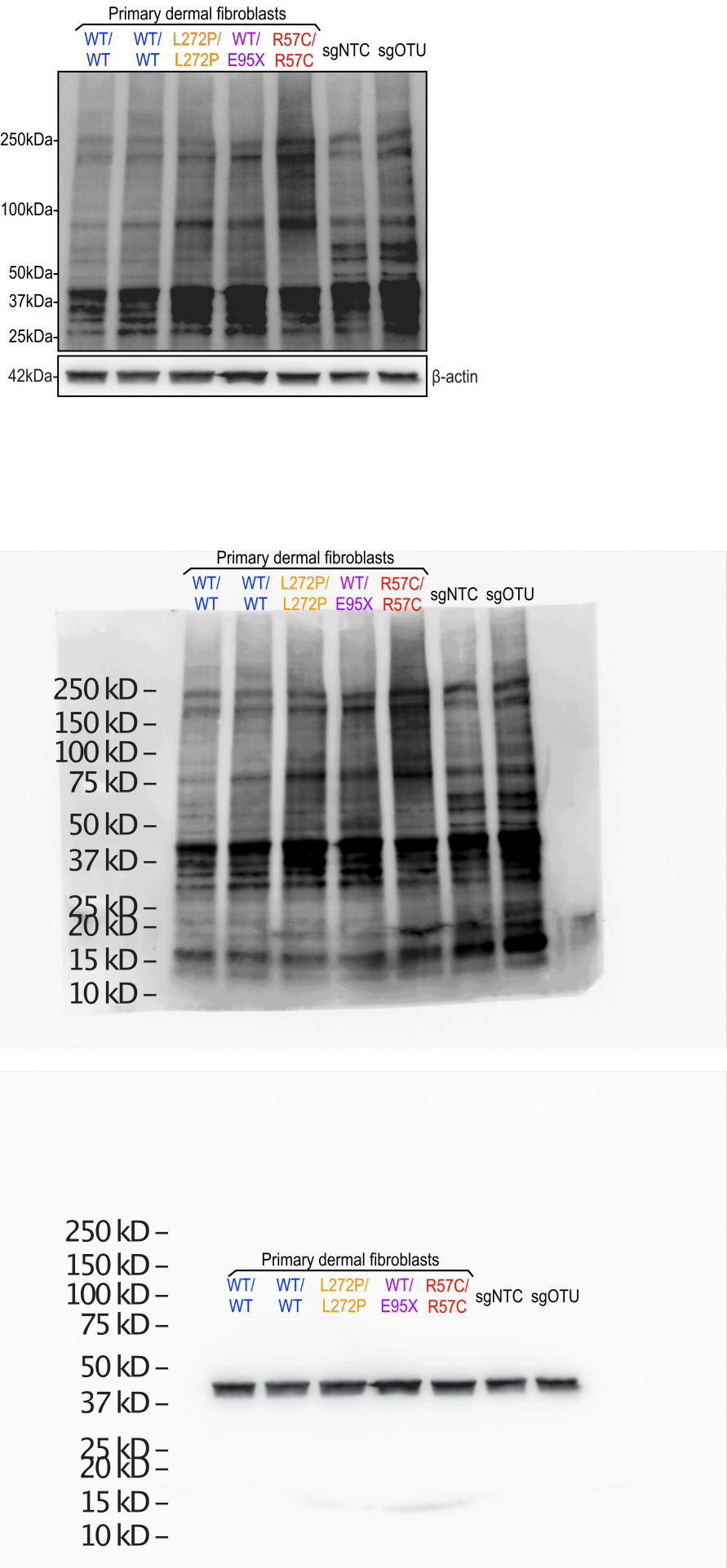

Supplement: Supplementary file 53 — Uncropped immunoblot Fig. 6a. [file 41590_2026_2568_MOESM53_ESM.pdf]

Figure 6B

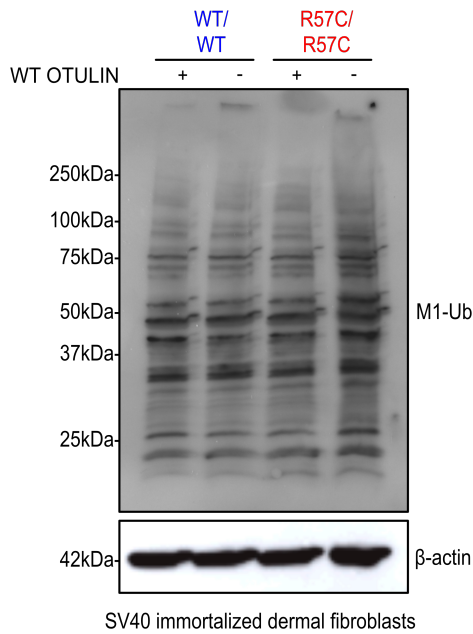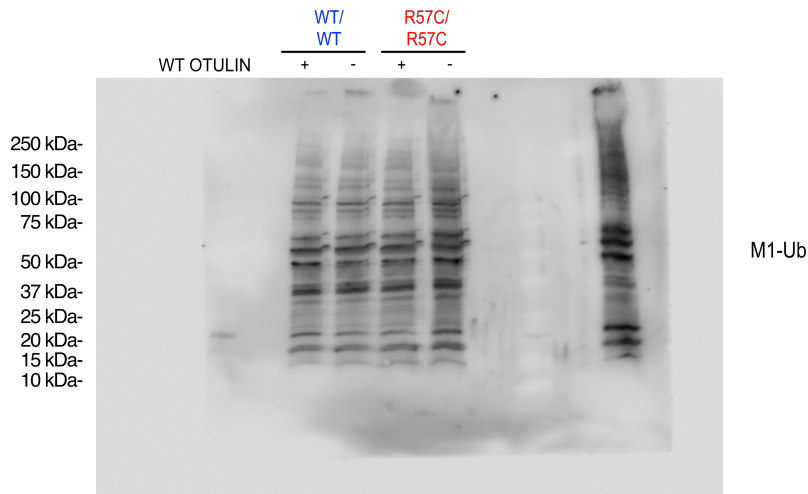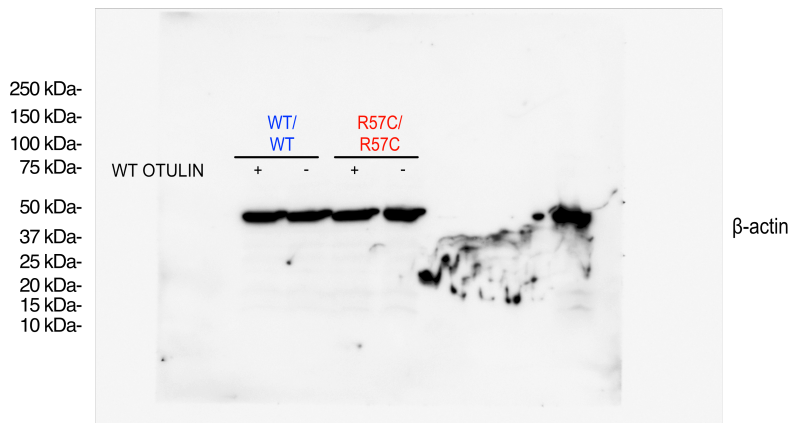

Supplement: Supplementary file 54 — Uncropped immunoblot Fig. 6b. [file 41590_2026_2568_MOESM54_ESM.pdf]

**Figure 6C**

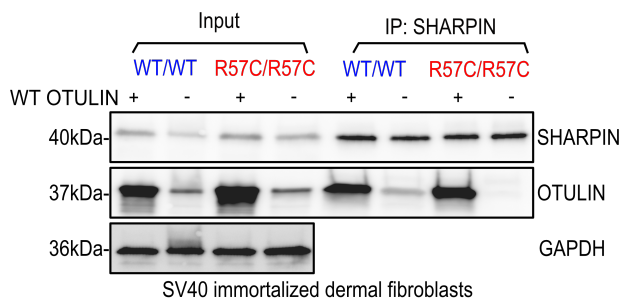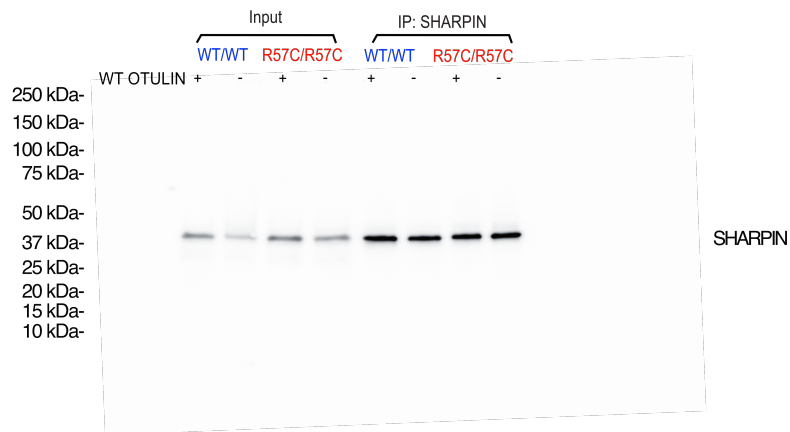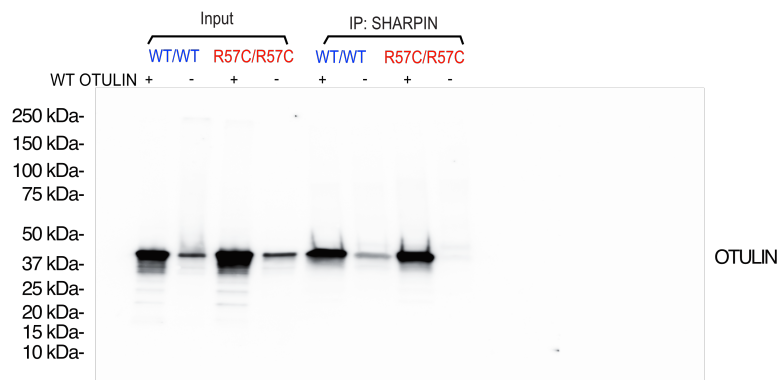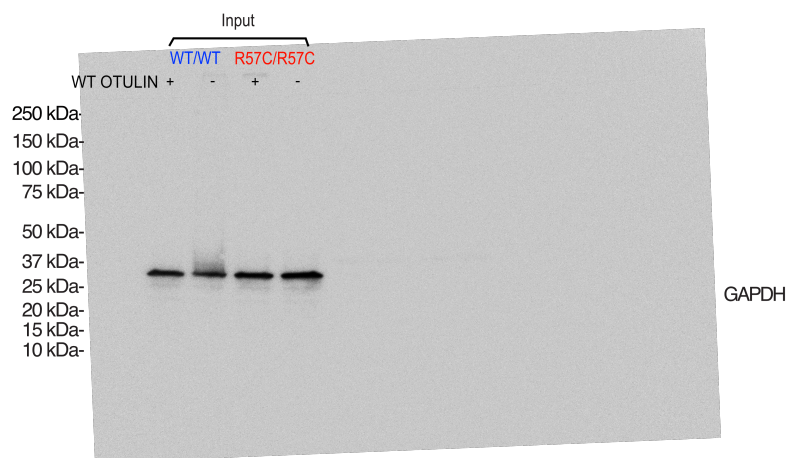

Supplement: Supplementary file 55 — Uncropped immunoblot Fig. 6c. [file 41590_2026_2568_MOESM55_ESM.pdf]

Figure 6D

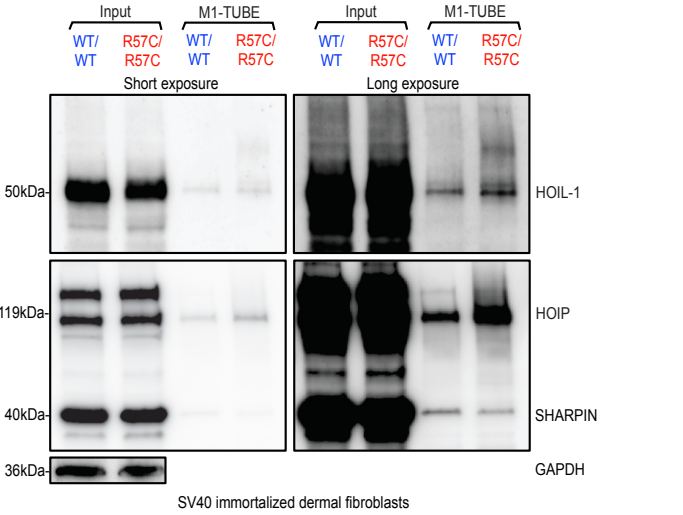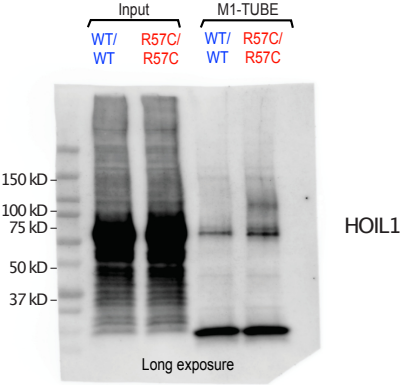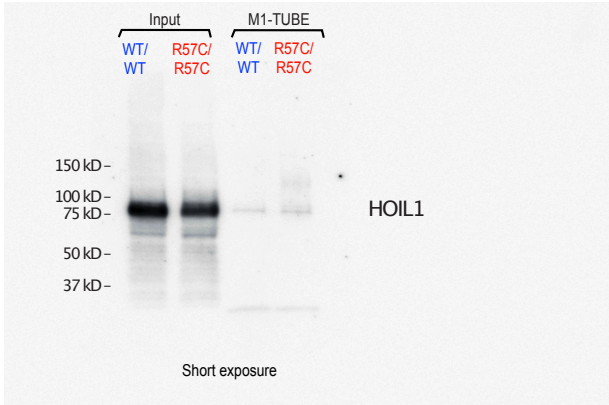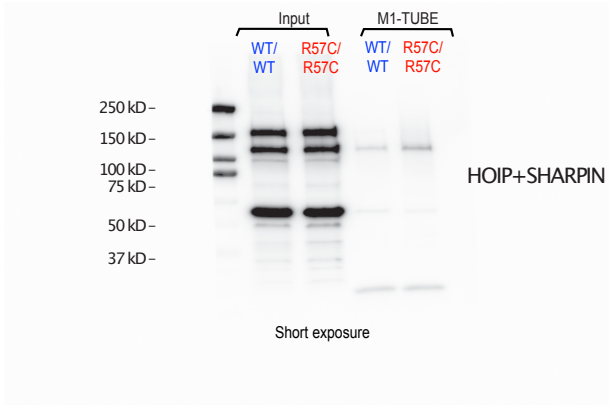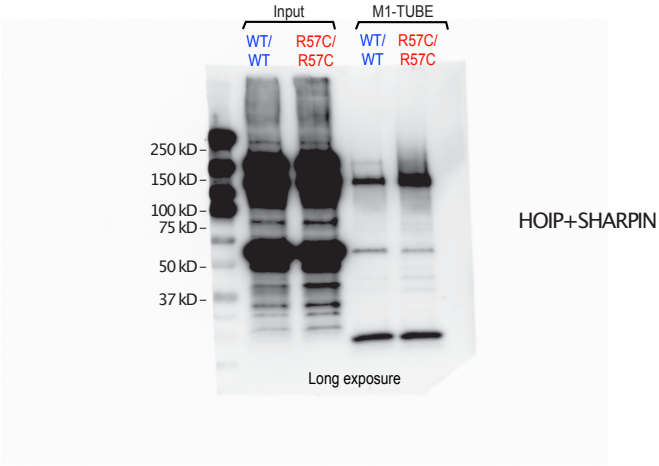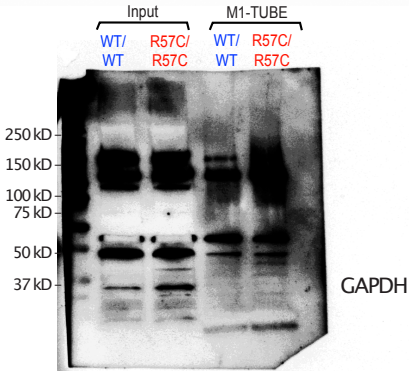

Supplement: Supplementary file 56 — Uncropped immunoblot Fig. 6d. [file 41590_2026_2568_MOESM56_ESM.pdf]

Figure 6E

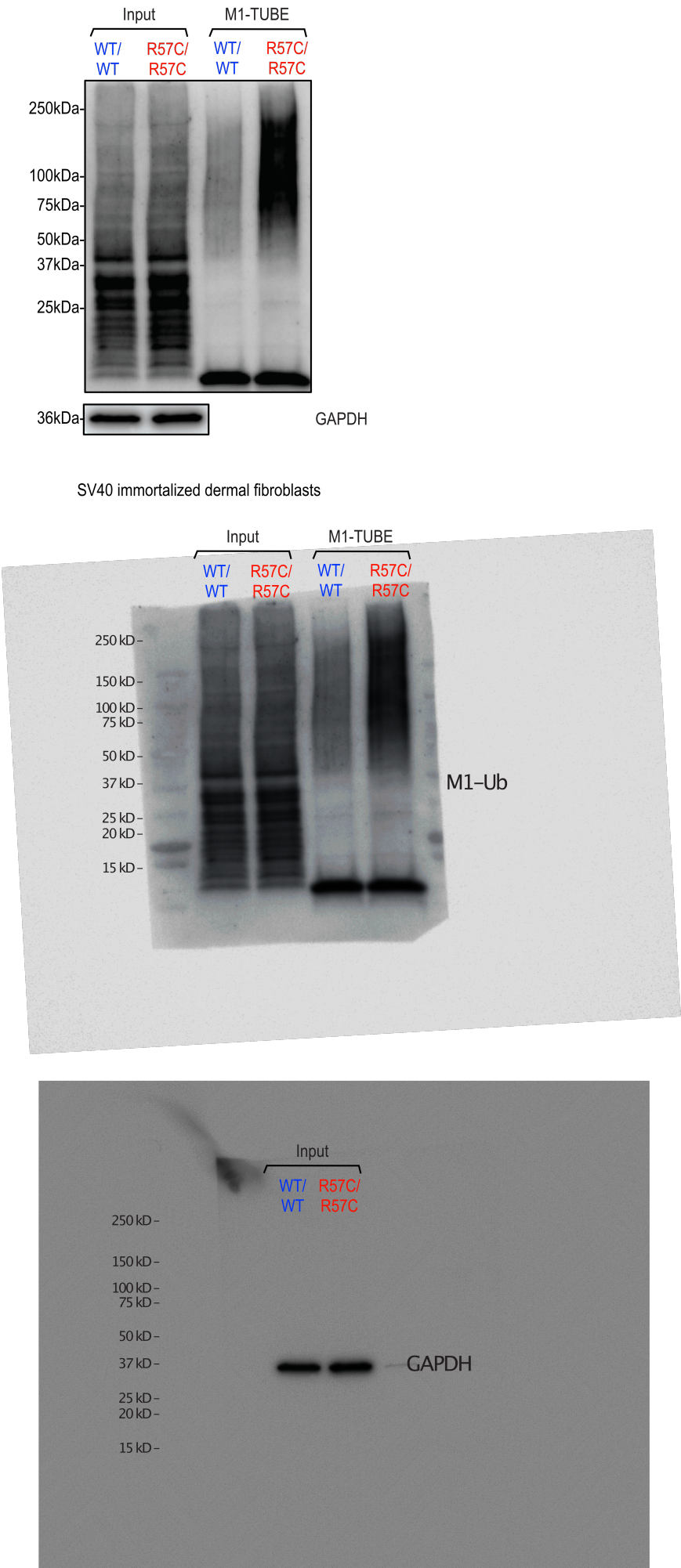

Supplement: Supplementary file 57 — Uncropped immunoblot Fig. 6e. [file 41590_2026_2568_MOESM57_ESM.pdf]

Figure 6F

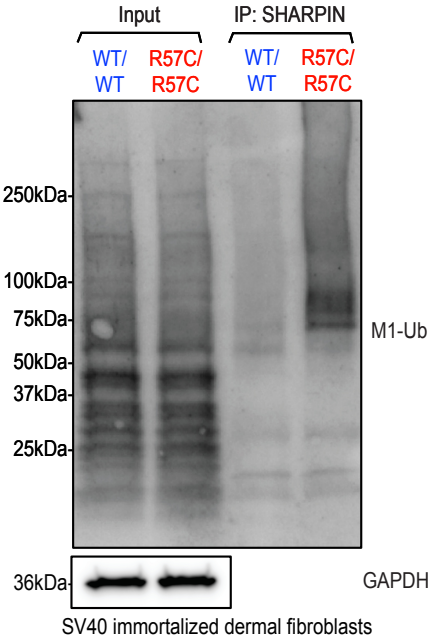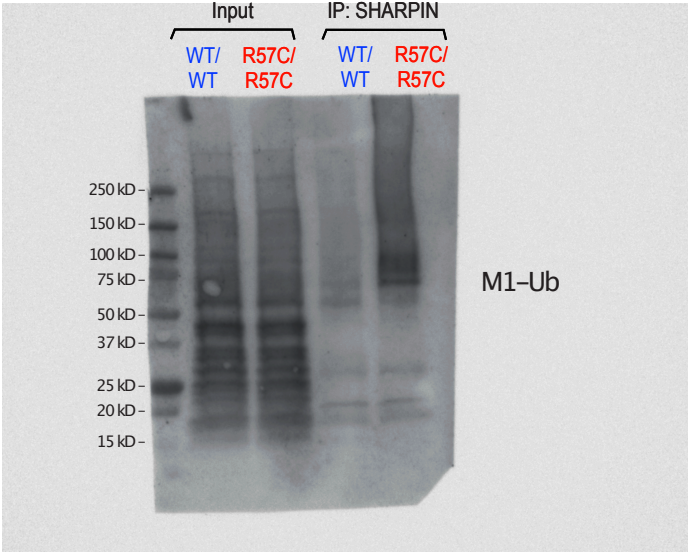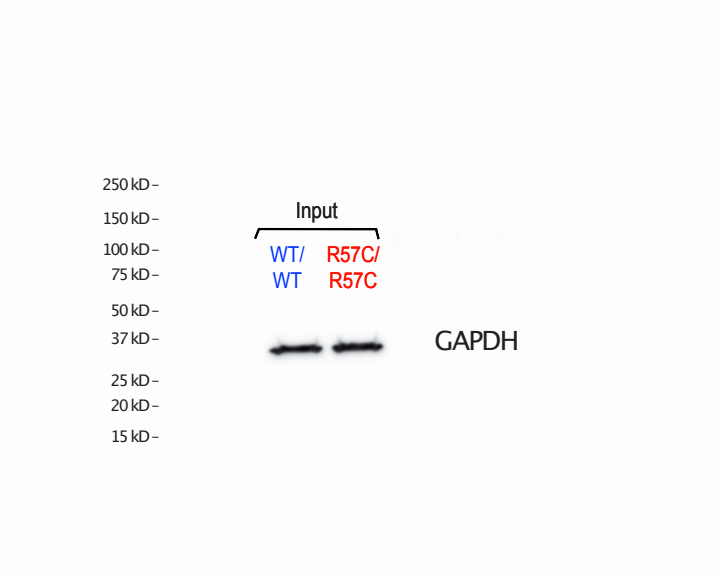

Supplement: Supplementary file 58 — Uncropped immunoblot Fig. 6f. [file 41590_2026_2568_MOESM58_ESM.pdf]

Figure 6G

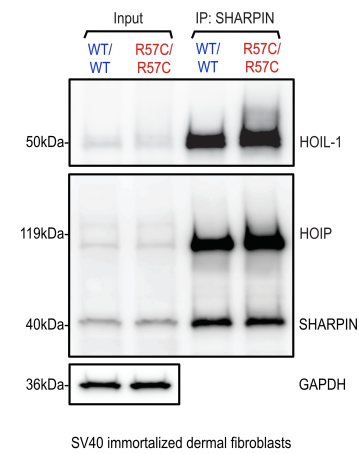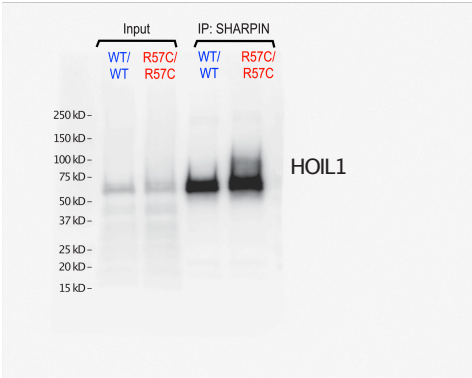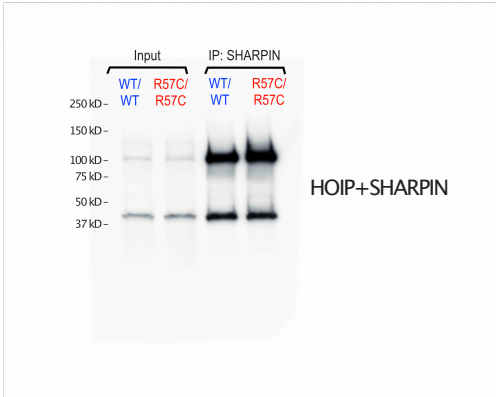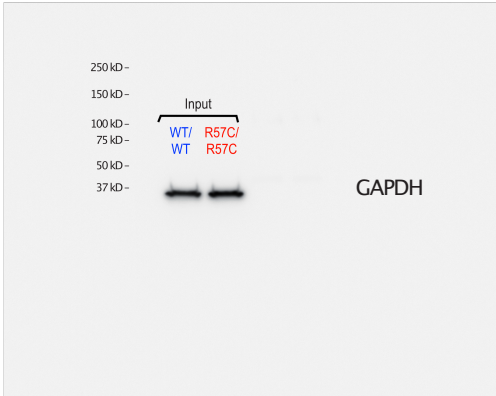

Supplement: Supplementary file 59 — Uncropped immunoblot Fig. 6g. [file 41590_2026_2568_MOESM59_ESM.pdf]

Figure 6H

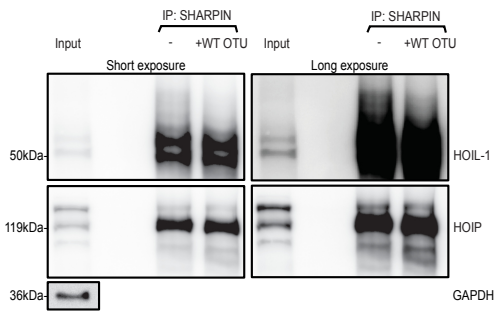

P3 (R57C/R57C)  
SV40 immortalized dermal fibroblasts

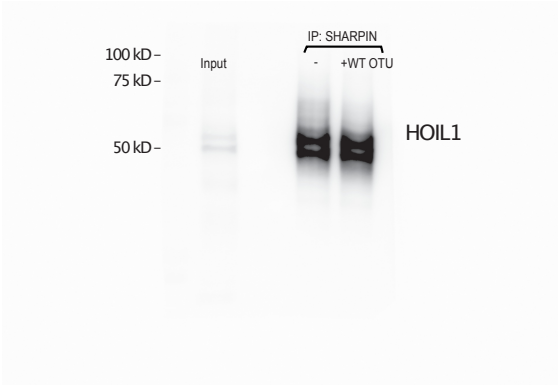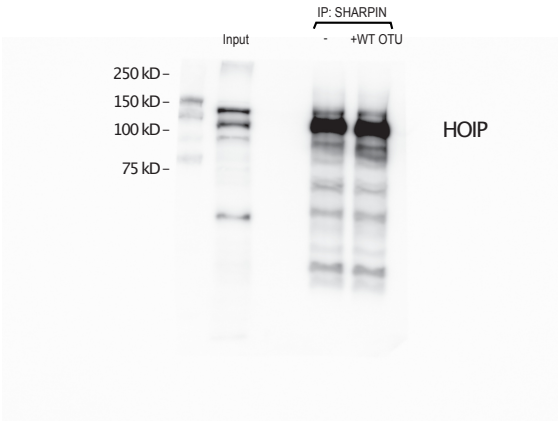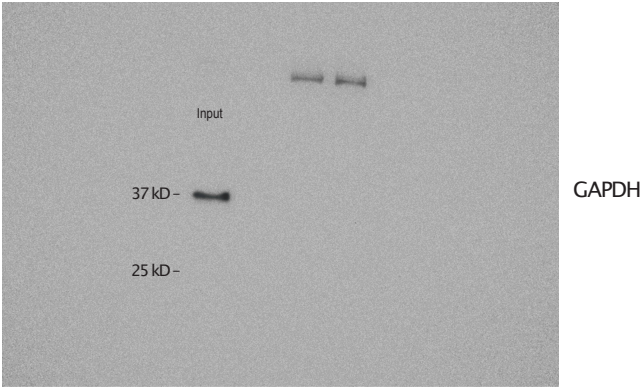

Supplement: Supplementary file 60 — Uncropped immunoblot Fig. 6h. [file 41590_2026_2568_MOESM60_ESM.pdf]
