# Supplementary material for: Pyoderma gangrenosum caused by the molecular uncoupling of OTULIN catalytic activity and LUBAC binding
Source: Nat Immunol. 2026 Jun 15;27(8):1619–32. doi: 10.1038/s41590-026-02568-6 (PMC13414579; doi:10.1038/s41590-026-02568-6)

Extended Data Figure 1D (input blots only)

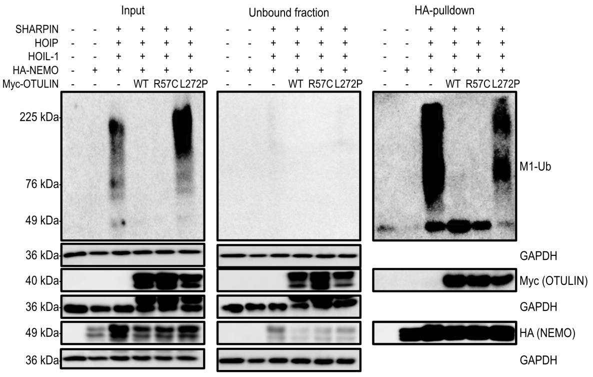

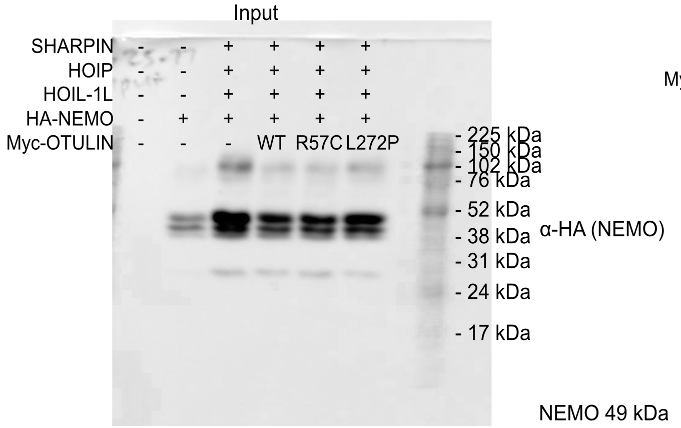

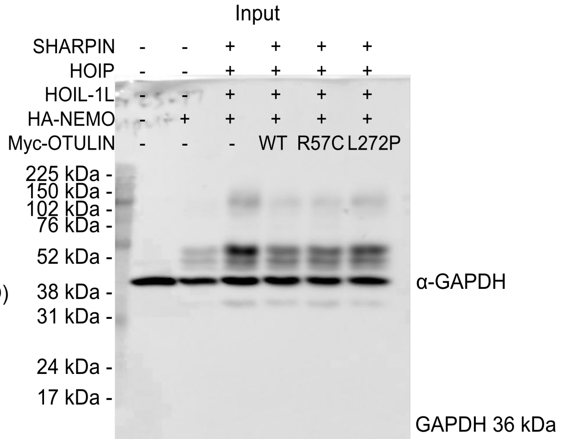

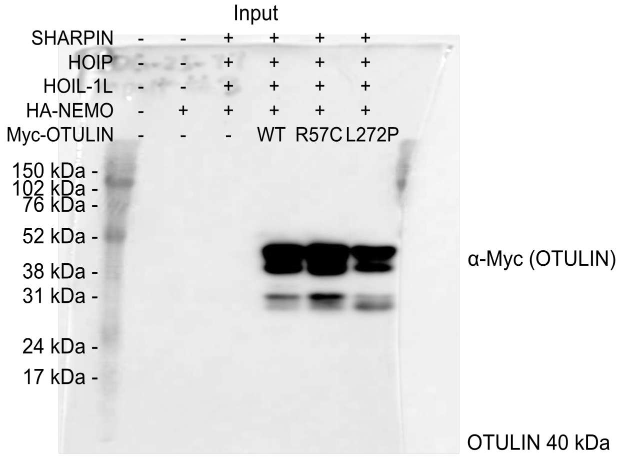

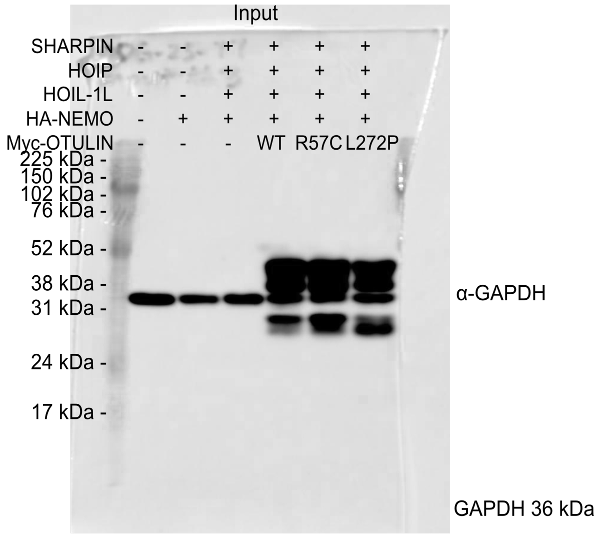

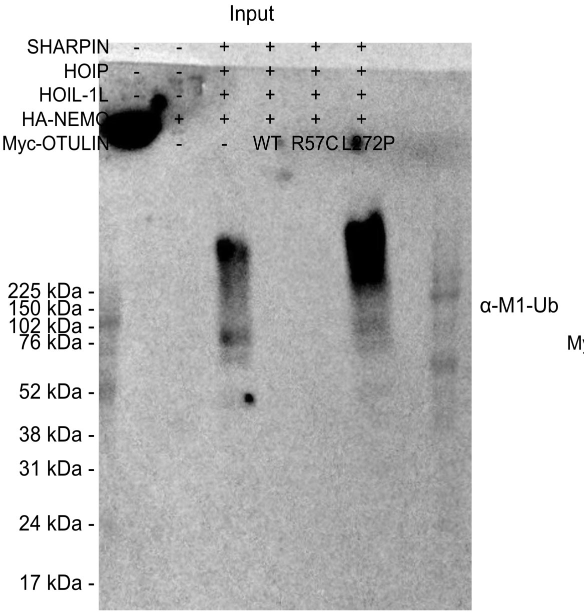

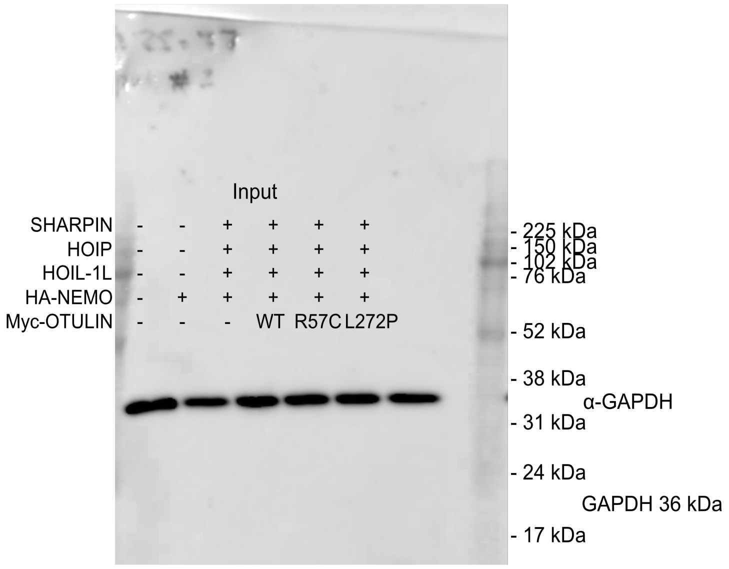

Extended Data Figure 1D (unbound fraction blots only)

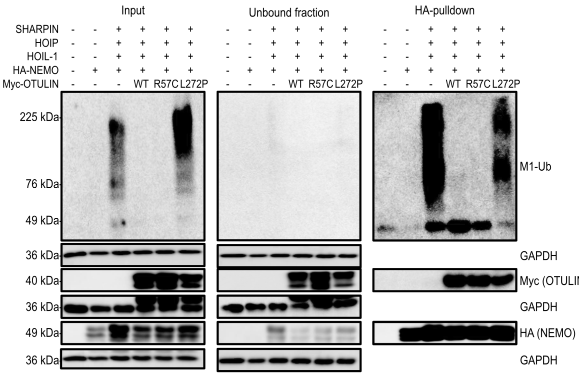

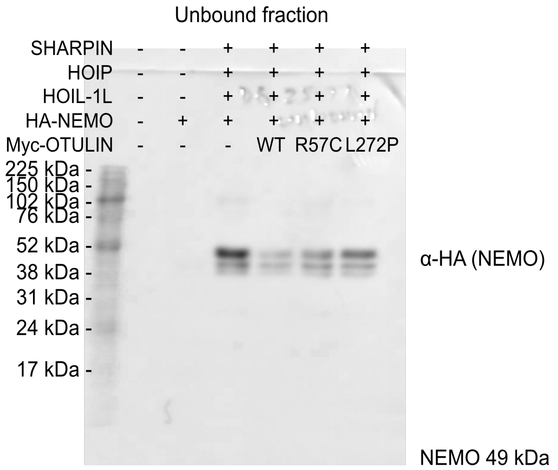

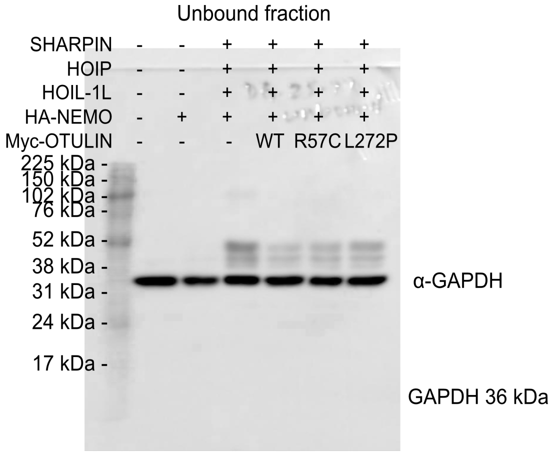

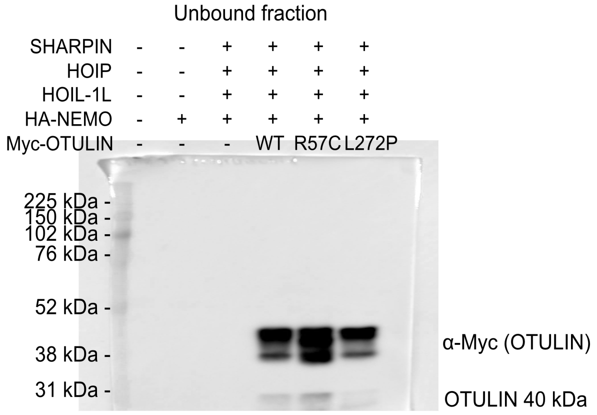

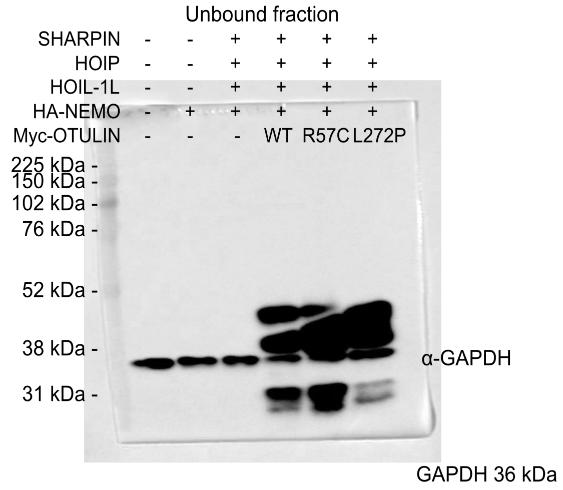

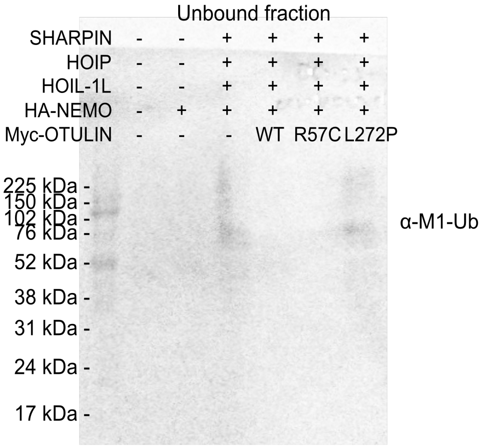

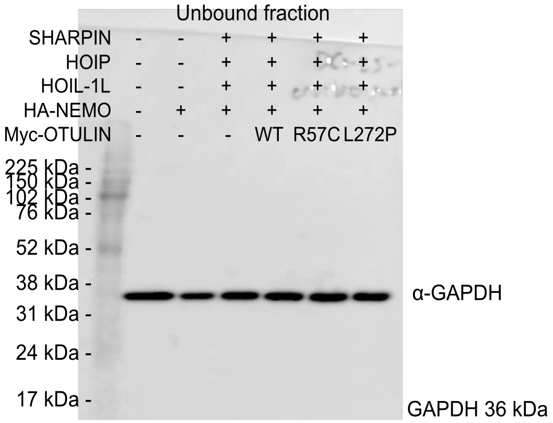

Extended Data Figure 1D (IP blots only)

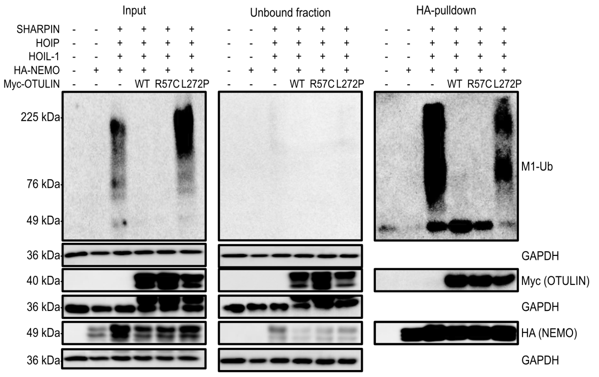

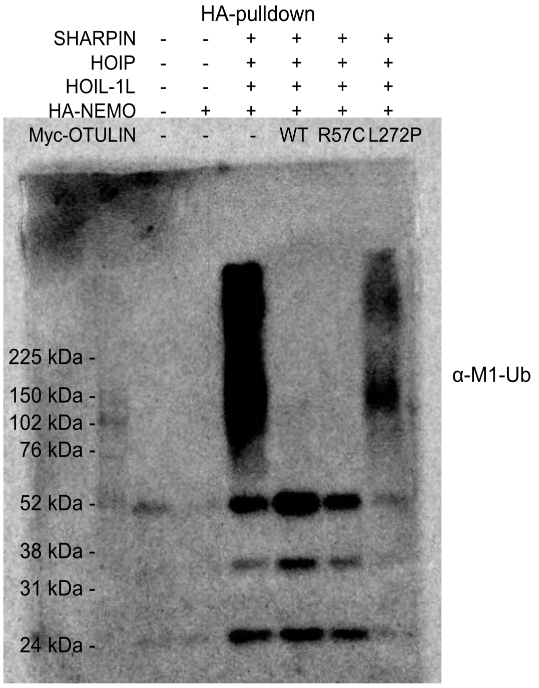

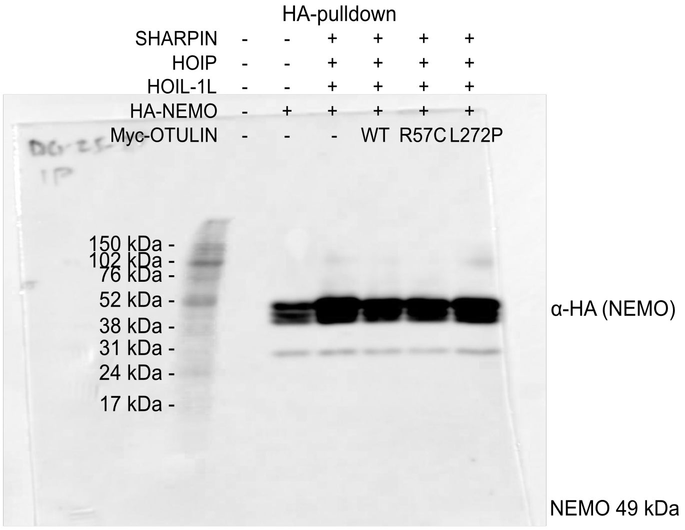

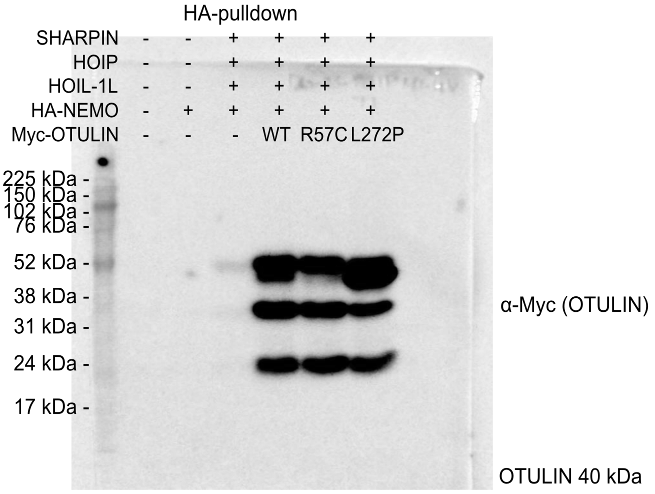

Supplement: Supplementary file 31 — Uncropped immunoblot Extended Data Fig. 1d. [file 41590_2026_2568_MOESM31_ESM.pdf]
